# Autoacetylation of the *Ralstonia solanacearum* Effector PopP2 Targets a Lysine Residue Essential for RRS1-R-Mediated Immunity in Arabidopsis

**DOI:** 10.1371/journal.ppat.1001202

**Published:** 2010-11-18

**Authors:** Céline Tasset, Maud Bernoux, Alain Jauneau, Cécile Pouzet, Christian Brière, Sylvie Kieffer-Jacquinod, Susana Rivas, Yves Marco, Laurent Deslandes

**Affiliations:** 1 Laboratoire des Interactions Plantes Microorganismes (LIPM), UMR CNRS-INRA 2594/441, Castanet-Tolosan, France; 2 Institut Fédératif de Recherche 40, Plateforme Imagerie, Pôle de Biotechnologie Végétale, Castanet-Tolosan, France; 3 Surfaces Cellulaires et Signalisation chez les Végétaux, Université de Toulouse, UMR CNRS-Université Paul Sabatier 5546, Castanet-Tolosan, France; 4 CEA, DSV/IRTSV Laboratoire EdyP, Grenoble, France; The University of North Carolina at Chapel Hill, United States of America

## Abstract

Type III effector proteins from bacterial pathogens manipulate components of host immunity to suppress defence responses and promote pathogen development. In plants, host proteins targeted by some effectors called avirulence proteins are surveyed by plant disease resistance proteins referred to as “guards”. The *Ralstonia solanacearum* effector protein PopP2 triggers immunity in Arabidopsis following its perception by the RRS1-R resistance protein. Here, we show that PopP2 interacts with RRS1-R in the nucleus of living plant cells. PopP2 belongs to the YopJ-like family of cysteine proteases, which share a conserved catalytic triad that includes a highly conserved cysteine residue. The catalytic cysteine mutant PopP2-C321A is impaired in its avirulence activity although it is still able to interact with RRS1-R. In addition, PopP2 prevents proteasomal degradation of RRS1-R, independent of the presence of an integral PopP2 catalytic core. A liquid chromatography/tandem mass spectrometry analysis showed that PopP2 displays acetyl-transferase activity leading to its autoacetylation on a particular lysine residue, which is well conserved among all members of the YopJ family. These data suggest that this lysine residue may correspond to a key binding site for acetyl-coenzyme A required for protein activity. Indeed, mutation of this lysine in PopP2 abolishes RRS1-R-mediated immunity. In agreement with the guard hypothesis, our results favour the idea that activation of the plant immune response by RRS1-R depends not only on the physical interaction between the two proteins but also on its perception of PopP2 enzymatic activity.

## Author Summary

Plant and animal bacterial pathogens have evolved to produce virulence factors, called type III effectors, which are injected into host cells to suppress host defences and provide an environment beneficial for pathogen growth. Type III effectors from pathogenic bacteria display enzymatic activities, often mimicking an endogenous eukaryotic activity, to target host signalling pathways. Elucidation of strategies used by pathogens to manipulate host protein activities is a subject of fundamental interest in pathology. PopP2 is a YopJ-like effector from the soil borne root pathogen *Ralstonia solanacearum.* Here, in addition to demonstrating PopP2 ability to stabilize the expression of its cognate Arabidopsis RRS1-R resistance protein and physically interact with it, we investigated the enzymatic activity of PopP2. Bacterial YopJ-like effectors are predicted to act as acetyl-transferases on host components. However, only two YopJ-like proteins from animal pathogens have been shown to be active acetyl-transferases. We show that PopP2 displays autoacetyl-transferase activity targeting a lysine residue well-conserved among YopJ-like family members. This lysine is a critical residue since its mutation prevents autoacetylation of PopP2 and abolishes its recognition by the host. This study provides new clues on the multiple properties displayed by bacterial type III effectors that may be used to target defense-related host components.

## Introduction

To defend themselves against pathogen infection, plants have evolved a bipartite, inducible innate immune system that efficiently recognizes and wards off pathogens [1]–[3]. Perception of conserved molecules, essential to many pathogens and called pathogen-associated molecular patterns (PAMPs), triggers the so-called PTI (PAMP-Triggered Immunity), which represents the first layer of host defence [4]. The recognition of different PAMPs occurs by specific pattern recognition receptors (PRRs) acting at the plant cell surface to activate various defence responses in the host [1], [5], [6]. Plants can also perceive specific effectors [previously referred to as avirulence (Avr) proteins] through additional receptors –typically nucleotide binding site-leucine rich repeat (NB-LRR) resistance (R) proteins. This second layer of defence is called effector-triggered immunity (ETI). The simplest model to explain perception of a given Avr protein by the corresponding R protein is that they physically interact as a ligand-receptor couple, leading to activation of immunity. Nevertheless, direct interaction between Avr and R proteins has been only described in a few cases indicating that this type of recognition is an exception rather than the rule [7]–[10]. To explain the lack of evidence for direct interaction for most of known Avr-R pairs, the guard model was proposed [11], [12]. This model, validated in the case of several R-Avr interactions, postulates that R proteins guard effector targets or “guardees” from effector-triggered manipulation in host cells [13]–[16]. Several effector targets have been identified and shown to play crucial a role in the establishment of plant resistance [17], [18].

The elucidation of the function of type III effectors (T3Es), and more specifically of their enzymatic activities perceived by guard proteins, remains a major challenge in phytopathology. One of the bacterial strategies to suppress host innate immunity consists in the manipulation or inactivation of plant components playing a role in defence-related signalling pathways [19]. Bacterial effectors exhibit diverse activities that mimic eukaryotic functions involved in defence against infection. Known biochemical activities of T3Es include manipulation of host protein turnover, either through protease activity [14], [20], [21] or protein degradation *via* the 26S proteasome [22]–[24], modification of host transcription or RNA stability [25]–[28] and alteration of the phosphorylation state of plant proteins [29]–[34].

The *RRS1-R* resistance gene, present in *Arabidopsis thaliana* plants of the Nd-1 ecotype, confers broad-spectrum resistance to several strains of *Ralstonia solanacearum. R. solanacearum* is the causal agent of bacterial wilt in more than 200 plant species, including agronomically important crop plants of the Solanaceous family [35]. *RRS1-R* encodes an R protein with original structure since it belongs to the Toll/Interleukin1 receptor (TIR)-NBS-LRR subclass of R proteins and presents a C-terminal WRKY motif that is characteristic of the zinc-finger class of WKRY plant transcription factors [36]. Although genetically defined as recessive, *RRS1-R* behaves as a dominant gene in transgenic Arabidopsis plants. The dominant *RRS1-S* allele from the Col-0 susceptible accession encodes a highly similar TIR-NBS-LRR-WRKY protein lacking the last 96 amino acids of the RRS1-R protein [37], [38].

**Figure 1:**
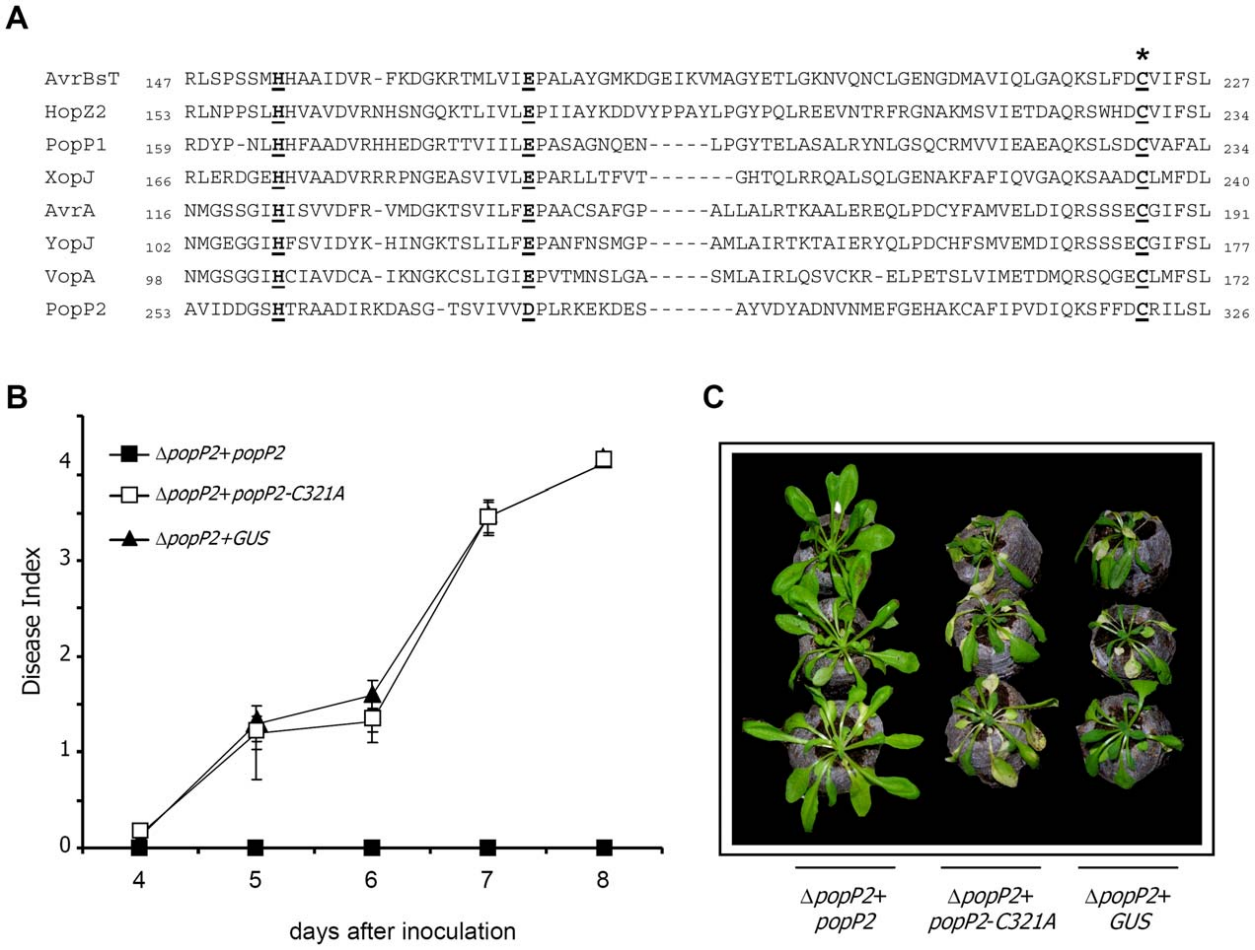
Mutation of cysteine 321 in PopP2 leads to loss of PopP2-triggered immunity on RRS1-R expressing plants. **A**: Sequence alignment of representative members of the YopJ-like effector family from plant and animal bacterial pathogens showing the conserved catalytic triad. Conserved residues in the catalytic core (H, D/E, C) are underlined and shown in bold. The star indicates the position of the main cysteine catalytic residue. Accession numbers for the proteins are: *Xanthomonas campestris* pv. *vesicatoria* AvrBsT (AAD39255); *Pseudomonas syringae pv. syringae* HopZ2 (ABK13722); *R. solanacearum* PopP1 (CAF32331) and PopP2 (CAD14570); *Xanthomonas campestris* pv. *vesicatoria* XopJ (YP_363887); *Salmonella enterica* AvrA (AAB83970); *Yersinia pestis* YopJ (NP_395205); and *Vibrio parahaemolyticus* VopA (AAT08443). **B**: Disease symptom development curves. Individual Nd-1 (RRS1-R) Arabidopsis plants were scored 4, 5, 6, 7 and 8 days after inoculation *R. solanacearum* Δ*popP2* strain expressing wild-type PopP2 (black squares), PopP2-C321A (white squares) or a GUS control (black triangles), using the following scale: 0  =  no wilting, 1 = 25%, 2 = 50%, 3 = 75%, and 4  = 100% of wilted leaves. Mean and SD values were calculated from scores of a total of 40 plants (from three independent experiments). **C**: Phenotypic responses of Nd-1 (RRS1-R) Arabidopsis plants 8 days after inoculation with the indicated bacterial strains.

Among the more than 80 putative type three effectors (T3Es) encoded by *R. solanacearum*, PopP2 elicits RRS1-R-mediated specific disease resistance in Arabidopsis [9]. PopP2 belongs to the YopJ-like family of effectors. YopJ-like proteins are found in mammalian and plant pathogens, suggesting that they play important roles in the interaction with the host. Based on their structural characteristics, YopJ-like effectors have been assigned to the C55 peptidase family of the clan CE of cysteine proteases, which share a nucleophile cysteine and a predicted catalytic core composed of three conserved amino acid residues (Figure 1A) [39]–[41]. YopJ-like family members, such as AvrRxv and AvrXv4 from *Xanthomonas campestris* pv. *vesicatoria,* are SUMO (Small Ubiquitin-like MOdifier) isopeptidases [42]. It was proposed that removal of SUMO from host plant proteins would allow binding of ubiquitin leading to protein degradation. The YopJ protein from *Yersinia* spp displays de-ubiquitinating, de-SUMOylating and acetylating activities. YopJ-mediated acetylation of critical serine and threonine residues in target MAPKs, which are essential for the host cell inflammatory response, blocks their activation by phosphorylation [43]–[45].

Co-expression of PopP2 and either RRS1-R or RRS1-S in Arabidopsis protoplasts previously revealed that RRS1 proteins are specifically targeted to the plant cell nucleus. The interaction between PopP2 and RRS1-R/S was also demonstrated in yeast [9]. However, the enzymatic activity of PopP2 and its mode of action remain unknown. In this study, using the FRET-FLIM technique, the physical interaction between PopP2 and RRS1-R was shown to occur in the nucleus of *N. benthamiana* and Arabidopsis epidermal cells. In addition, we show that PopP2 stabilizes the expression of RRS1-R *in planta*. Similar results were obtained with the dominant *RRS1-S* gene product. Conservation of the YopJ catalytic core within the PopP2 sequence prompted us to test the hypothesis that PopP2 displays acetyl-transferase activity. We show that PopP2 autoacetylates on a conserved lysine residue essential for RRS1-R-mediated immunity in Arabidopsis.

## Results

### Conserved catalytic cysteine 321 in PopP2 is required to trigger RRS1-R-mediated resistance

A conserved catalytic triad is present in representatives of the clan CE of cysteine proteases, including the T3Es YopJ from *Y. pestis* and PopP2 from *R. solanacearum* (Figure 1A). If PopP2 avirulence function was dependent on its enzymatic activity, mutation of cysteine 321 in the predicted catalytic core to a non-reactive residue such as alanine (PopP2-C321A) should abolish PopP2 ability to trigger RRS1-R-mediated resistance in Arabidopsis. To test this hypothesis, a PopP2-C321A mutant version was engineered. PopP2 and PopP2-C321A were next tagged at their C-terminus with a triple hemagglutinin (3x-HA) epitope, and expressed under the control of the native *popP2* promoter. These constructs were introduced in a previously described strain of *R. solanacearum* (Δ*popP2*) from which the *popP2* gene had been deleted, and therefore no longer recognized by Arabidopsis Nd-1 plants carrying the *RRS1-R* gene [9]. Nd-1 Arabidopsis plants were inoculated with the Δ*popP2* strain expressing either PopP2, PopP2-C321A, or a GUS control. As a consequence of the recognition of PopP2 by RRS1-R, Nd-1 plants remained symptomless 8 days after inoculation with Δ*popP2* carrying wild-type *popP2*, whereas complete wilting was observed after inoculation with the two Δ*popP2* strains expressing either mutant PopP2-C321A or the GUS control (Figure 1B,C). These data demonstrate that mutation of the conserved putative catalytic C321 in PopP2 abolishes its ability to trigger an RRS1-R-specific resistance response in Arabidopsis.

### PopP2 interacts with RRS1-R in living plant cells independent of the presence of its integral catalytic core

**Figure 2:**
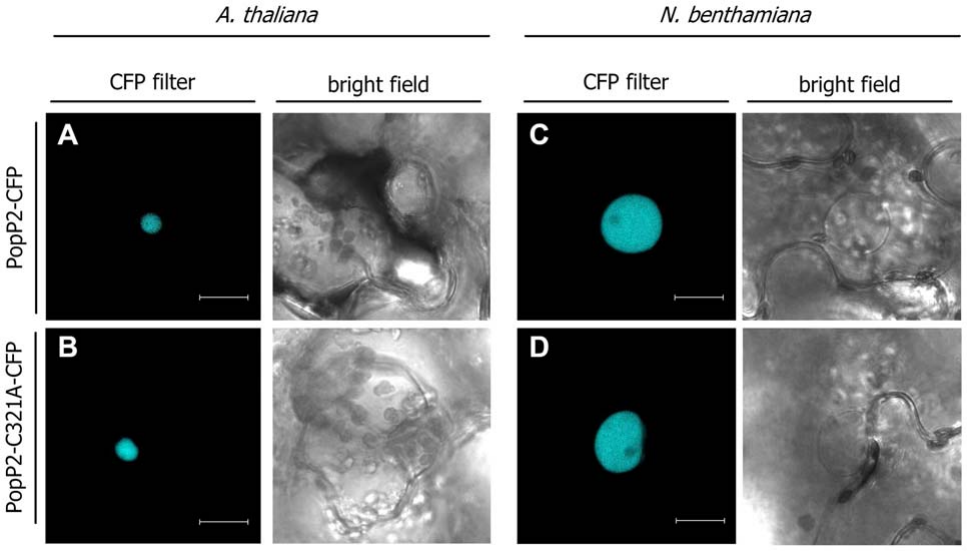
Mutation of the conserved cysteine in PopP2 catalytic triad does not affect its nuclear localization *in planta*. Confocal images of *A. thaliana* (**A,B**) and *N. benthamiana* (**C,D**) leaf epidermal cells after *Agrobacterium*-mediated transient expression of P35S:PopP2-CFP and P35S:PopP2-C321A-CFP. Pictures were taken 72 (A, B) or 48 (C, D) hours after agroinfiltration. Bar =  20 µm.

PopP2 is targeted to the plant cell nucleus where it is predicted to physically interact with RRS1-R, as indicated by previously published yeast two-hybrid data [9]. The inability of mutant PopP2-C321A to initiate the RRS1-R resistance response is consistent with the hypothesis that PopP2 functions as a cysteine protease and that its enzymatic activity is perceived by RRS1-R. Alternatively, we cannot exclude that the C321A mutation leads to a conformational change in PopP2 that may impair its nuclear targeting and/or its ability to interact with RRS1-R. *Agrobacterium*-mediated transient expression of PopP2 and PopP2-C321A fused to CFP (Cyan Fluorescent Protein), under the control of a 35S promoter, led to detection of a fluorescent signal in the nucleus of both Arabidopsis and *N. benthamiana* epidermal cells (Figure 2), demonstrating that mutation of the conserved cysteine residue in the catalytic triad of PopP2 does not affect its nuclear targeting.

**Figure 3:**
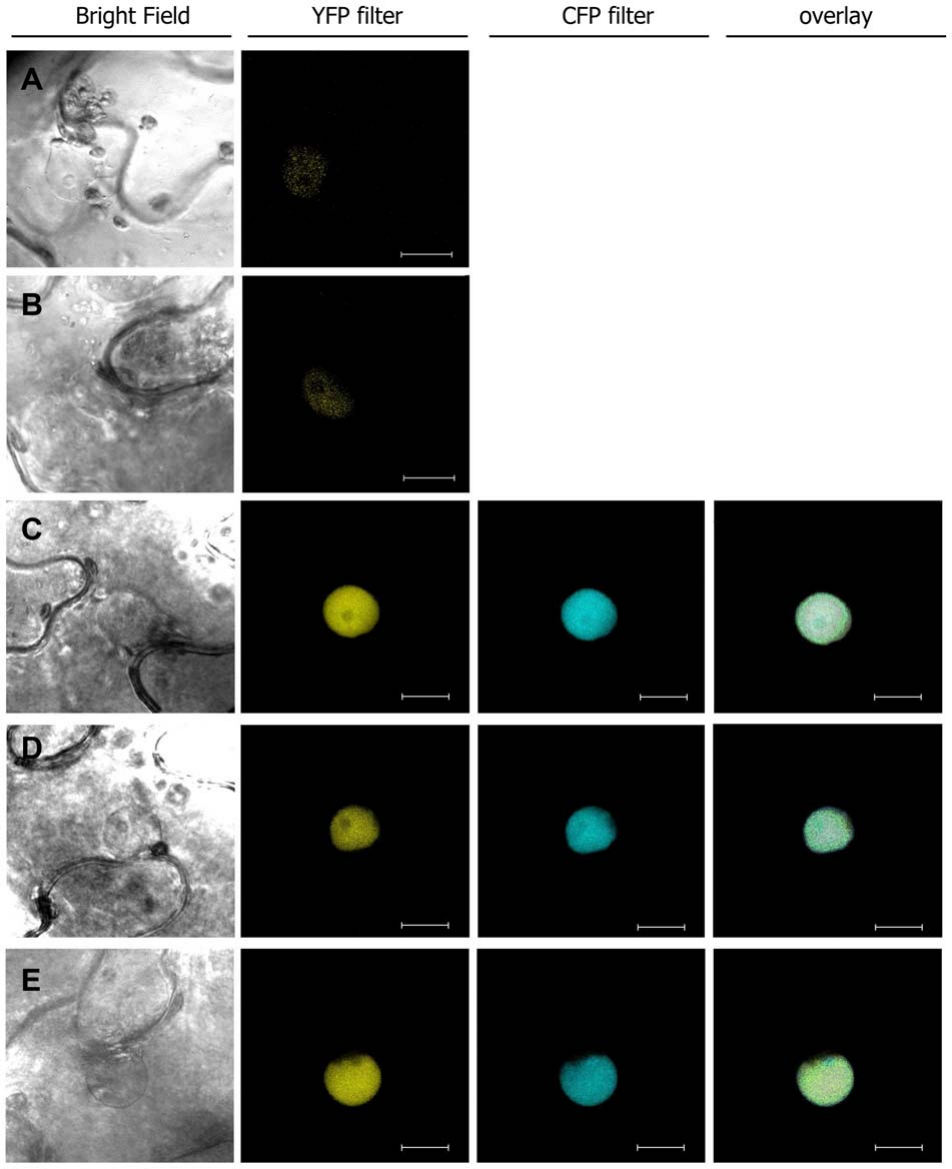
RRS1-R-YFPv and RRS1-S-YFPv co-localize with PopP2-CFP in the nucleus of *N. benthamiana* epidermal cells. Confocal images of *N. benthamiana* epidermal cells, 48 hours after *Agrobacterium-*mediated transient expression of P35S:RRS1-R-YFPv (**A**), P35S:RRS1-S-YFPv (**B**), P35S:RRS1-R-YFPv + P35S:PopP2-CFP (**C**), P35S:RRS1-R-YFPv +P35S:PopP2-C321A-CFP (**D**), and P35S:RRS1-S-YFPv + P35S:PopP2-CFP (**E**). Bar =  20 µm.

**Figure 4:**
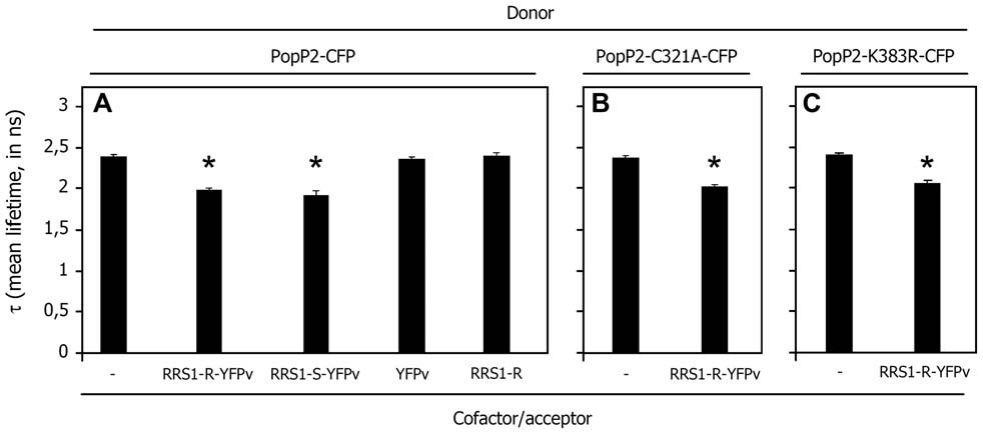
PopP2, PopP2-C321A and PopP2-K383R interact with RRS1-R in the plant cell nucleus. Mean lifetime measurements of PopP2-CFP (**A**), PopP2-C321A-CFP (**B**) and PopP2-K383R-CFP (**C**) expressed alone (-) or in the presence of RRS1-R-YFPv, RRS1-S-YFPv, YFPv or RRS1-R, as indicated. Total number of nuclei scored for each combination and corresponding standard error data of the mean lifetime are listed in Table 1.

A quantitative non-invasive fluorescence lifetime imaging (FLIM) approach was then used to monitor the physical interaction between RRS1-R and PopP2. Expression of RRS1-R fused to YFPv (Yellow Fluorescent Protein venus) was not detectable in Arabidopsis cells whereas a weak fluorescent signal was observed within the nuclei of *N. benthamiana* cells after transient expression using *Agrobacterium* (Figure 3A). Therefore, FRET-FLIM studies were conducted by co-expressing PopP2, or PopP2-C321A, fused to CFP, together with RRS1-R-YFPv in *N. benthamiana*. This transient expression system was previously used to demonstrate the physical interaction between PopP2 and RD19, a vacuolar Arabidopsis cysteine protease that is relocalized to the nucleus in the presence of PopP2 [46]. The average CFP lifetime in nuclei expressing PopP2-CFP was 2.386±0.030 ns (mean ± SEM). A significant reduction of the average CFP lifetime to 1.979±0.024 ns (p-value = 6.4×10^−21^) was measured in nuclei co-expressing the PopP2-CFP and RRS1-R-YFPv fusion proteins (Figure 3C,4A; Table 1), showing that RRS1-R is able to interact with PopP2 in the nucleus. Mutant PopP2-C321A also interacts with RRS1-R, as shown by a significant reduction of the average CFP lifetime in nuclei co-expressing PopP2-C321A-CFP and RRS1-R-YFPv, as compared to nuclei expressing PopP2-C321A-CFP alone (Figure 3D,4B; Table 1). This result indicates that the nuclear interaction between PopP2 and RRS1-R does not depend on the presence of the conserved cysteine residue in the catalytic triad of PopP2. To confirm that reduction of PopP2-CFP lifetime, in the presence of RRS1-R-YFPv, is not due to non-specific transfer of energy between the two fluorophores, PopP2-CFP was co-expressed with either untagged RRS1-R or YFPv. No physical interaction could be detected in either case, as shown by an average CFP lifetime which is not significantly different from that of PopP2-CFP alone (Figure 4A and Table 1). Importantly, the interaction between PopP2 and RRS1-R was confirmed in Arabidopsis cells after transient co-expression of both proteins (Table 1). Taken together, these data demonstrate a specific interaction between PopP2 and RRS1-R *in planta*, and strongly suggest that this protein association is independent of the enzymatic activity of PopP2.

**Table 1:** FRET-FLIM measurements showing that RRS1-R and RRS1-S proteins interact with PopP2 in the nucleus of plant cells.

Transient assay in	Donor	Acceptor	τ.	sem^(a)^	Δ^b)^	N^(c)^	E^(d)^	p-value
*N. benthamiana*	PopP2-CFP	-	2.386	0.030	-	130	-	-
*N. benthamiana*	PopP2-CFP	RRS1-R-YFPv	1.979	0.024	407	110	19	6.4E^-21^
*N. benthamiana*	PopP2-CFP	YFPv	2.361	0.027	-	25	-	0.720
*N. benthamiana*	PopP2-CFP	RRS1-R	2.391	0.035	-	25	-	0.943
*N. benthamiana*	PopP2-C321A-CFP	-	2.375	0.019	-	30	-	-
*N. benthamiana*	PopP2-C321A-CFP	RRS1-R-YFPv	2.021	0.031	354	30	15	8.3E^−14^
*A. thaliana*	PopP2-CFP	-	2.297	0.035	-	10	-	-
*A. thaliana*	PopP2-CFP	RRS1-R-YFPv	1.872	0.031	425	8	18	1.5E^−07^
*N. benthamiana*	PopP2-CFP	RRS1-S-YFPv	1.889	0.031	497	81	21	1.8E^−22^
*N. benthamiana*	PopP2-K383R-CFP	-	2.362	0.027	-	30	-	-
*N. benthamiana*	PopP2-K383R-CFP	RRS1-R-YFPv	2.064	0.023	298	46	12.6	3.4E^−12^

*Mean lifetime, τ, in ns. ^(a)^ for each nucleus, average fluorescence decay profiles were plotted and fitted with exponential function using a non linear square estimation procedure and the mean lifetime was calculated according to τ = Σ α_i_τ_i_^2^/Σ α_i_τ_i_ with I(t)  = Σ α_i_ e^−t/τi^ , standard error of the mean, ^(b)^ Δt = τ_D_ - τ_DA_ (in ps), ^(c)^ total number of measured nuclei , and ^(d)^ % FRET efficiency: E = 1 – (τ_DA_/τ_D_). p-value of the difference between the donor lifetimes in the presence and in the absence of acceptor (Student's *t* test).

Detection of the RRS1-R/PopP2 interaction prompted us to check whether the RRS1-S protein, present in susceptible Col-0 plants, was also able to associate with PopP2. RRS1-S was previously shown to colocalize with PopP2 in the nucleus of Arabidopsis protoplasts and the interaction between the two proteins was demonstrated in yeast [9]. Lack of *in planta* association between these two proteins might explain why RRS1-S is not able to trigger the resistance response [38]. To test this idea, RRS1-S-YFPv was co-expressed with PopP2-CFP in *N. benthamiana* epidermal cells. Reduction of the average CFP lifetime to 1.889±0.031 ns (p-value = 1.8×10^−22^) was detected in nuclei co-expressing PopP2-CFP and RRS1-S-YFPv fusion proteins (Figure 3E,4A and Table 1), demonstrating that, similarly to RRS1-R, RRS1-S associates with PopP2 in the nucleus. This result confirms the previously published RRS1-S/PopP2 interaction in yeast cells and indicates that the susceptibility of Col-0 plants to the Ralstonia GMI1000 strain cannot be explained by the lack of interaction between RRS1-S and PopP2. Taken together, our data strongly suggest that activation of the RRS1-R-mediated resistance response requires not only its physical interaction with PopP2, but also perception of PopP2 enzymatic activity.

### PopP2 stabilizes RRS1-R and RRS1-S protein accumulation *in planta*

**Figure 5:**
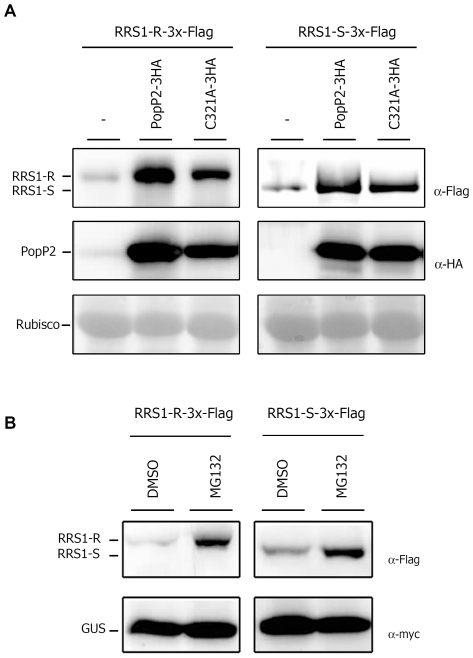
PopP2 protects RRS1-R and RRS1-S from proteasome-mediated degradation, independent of the integrity of its catalytic triad. **A**: *Agrobacterium*-mediated transient expression of RRS1-R-3x-Flag and RRS1-S-3x-Flag alone (-) or with either PopP2-3x-HA or PopP2-C321A-3x-HA in *N. benthamiana* epidermal cells. Leaf samples were harvested 48 hours after infiltration and subjected to immunoblot analysis with anti-Flag (top) or anti-HA (middle) antibodies. Rubisco is shown as loading control (bottom). **B**: Transient co-expression of RRS1-R-3x-Flag or RRS1-S-3x-Flag with GUS-myc in *N. benthamiana* epidermal cells using *Agrobacterium*. The proteasome inhibitor MG132 (or control DMSO) was applied 24 hours after agroinfiltration. Protein samples were harvested 12 h later and analyzed using anti-Flag (top) or anti-HA (bottom) antibodies.

Protein co-expression experiments *in planta* indicated that PopP2 may be able to increase the accumulation level of YFPv-tagged RRS1-R and RRS1-S (Figure 3A,C and 3B,E). This observation was further confirmed by transiently co-expressing RRS1-R or RRS1-S, tagged at their C-terminus with a triple Flag epitope (3x-Flag), and PopP2 tagged at its C-terminus with a 3x-HA epitope in *N. benthamiana*. Immunoblot analysis of total protein extracts showed higher accumulation of RRS1-R and RRS1-S in the presence of PopP2 (Figure 5A). Similar results were obtained with the PopP2 catalytic mutant C321A, suggesting that the ability of PopP2 to enhance *in planta* accumulation of RRS1 proteins is not dependent on its enzymatic activity.

To investigate the specificity of this observation, we next tested whether PopP2 may also promote stabilization of the previously described PopP2 interacting partner, RD19. No modification of the RD19 protein level was detected after co-expression with PopP2 (Figure S1), indicating that protein stabilization by PopP2 may be restricted to a subset of its interacting partners.

We next studied whether the turnover of RRS1 proteins is post-translationally regulated through proteasomal activity. In the presence of the proteasome inhibitor MG132, both RRS1-R and RRS1-S accumulated, whereas expression of a GUS control was not altered in the same conditions (Figure 5B).

Taken together, our data (i) show that PopP2 is able to specifically promote the accumulation of its cognate partners, RRS1-S and RRS1-R, regardless of the integrity of its catalytic triad, and (ii) strongly suggest that the interaction between PopP2 and RRS1-R/S may block a molecular mechanism that leads to RRS1-R/S proteasome-dependent degradation.

### PopP2 displays acetyl-transferase activity leading to autoacetylation of lysine 383

**Figure 6:**
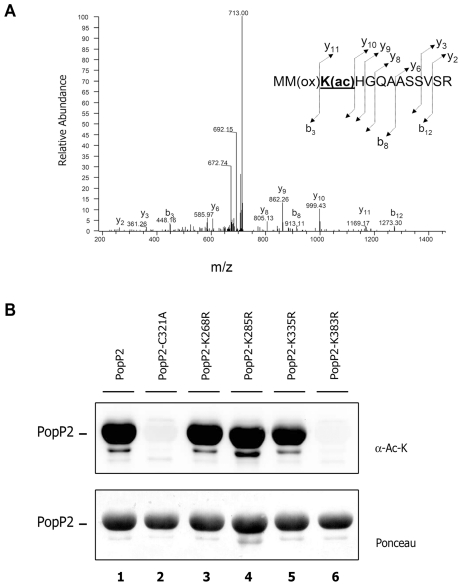
PopP2 is an active acetyl-transferase that autoacetylates on its lysine 383 residue. **A**: Mass spectrometric analysis of a PopP2 peptide spanning amino acid residues 381-393. Fragmentation of this peptide led to the identification of an acetylated lysine residue within the sequence MMK(ac)HGQAASSVSR [K(ac) indicates an acetylated lysine], in wild-type PopP2, but not PopP2-C321A. Labels b and y designate the N- and C-terminal fragments, respectively, of the peptide produced by cleavage at the peptide bond in the mass spectrometer. Numbers represent the number of N- or C-terminal residues present in the peptide fragment. **B**: Recognition of acetylated wild-type PopP2, but not PopP2-C321A or PopP2-K383R, by an anti-acetyl-lysine antibody. GST-PopP2, GST-PopP2-C321A, GST-PopP2-K268R, GST-PopP2-K285R, GST-PopP2-K335R and GST-PopP2-K383R proteins were expressed in *E. coli* and analysed by immunoblot using an antibody that recognizes acetylated lysines. GST-purified PopP2 recombinant proteins are shown after Ponceau staining (bottom).

The observation that PopP2-C321A is affected in its avirulence function strongly suggests that perception of the enzymatic activity of PopP2 is required to trigger RRS1-R-mediated resistance. YopJ and VopA are active acetyl-transferases previously described to be able to autoacetylate *in vitro* [43], [47]. We thus investigated whether PopP2, being a member of the YopJ-like family of effectors, exhibits a similar enzymatic activity. Towards this goal, GST-tagged PopP2 and PopP2-C321A were purified from overexpressing bacteria and subjected to liquid chromatography/tandem mass spectrometry (LC-MS/MS) to assess their acetylated states. Analysis of relative tryptic digestions revealed the presence of four acetylated lysine residues (at positions 268, 285, 335 and 383) in GST-PopP2 (Figure 6A, Table S1 and S2). However, acetylation of lysines 268, 285 and 335 is probably the result of endogenous *E. coli* acetyl-transferase activity, since it was also detected in mutant GST-PopP2-C321A, whereas acetylation of K383 is dependent on the presence of an integral PopP2 catalytic core. Together, these data strongly suggest that PopP2 is an active acetyl-transferase that autoacetylates at its K383 residue.

The differential acetylation status of GST-PopP2 and GST-PopP2-C321A was further investigated by immunoblot analysis using an antibody directed against acetylated lysine residues (α-Ac-K). This antibody allowed the detection of a strong signal corresponding to acetylated GST-PopP2 (Figure 6B, lane 1). In contrast, a barely detectable signal was observed in the case of GST-PopP2-C321A (Figure 6B, lane 2), perhaps due to weak acetylation at residues K268, K285, and K335 residues. Lysine to arginine substitutions were next engineered to generate GST-tagged PopP2-K268R, PopP2-K285R, PopP2-K335R and PopP2-K383R mutant versions. Importantly, PopP2-K268R, PopP2-K285R and PopP2-K335R, but not PopP2-K383R, were detected by immunoblot using the α-Ac-K antibody (Figure 6B), strongly indicating that only the K383R mutation prevents PopP2 autoacetylation (Figure 6B, lane 6). Furthermore, as in the case of the inactive GST-PopP2-C321A mutant, LC-MS/MS analysis of purified GST-PopP2-K383R led to the detection of a set of three acetylated peptides containing K268, K285 and K335 (Table S3). Together, these data confirm that (i) acetylation of K268, K285 and K335 is most likely due to an endogenous acetyl-transferase activity from *E. coli* and that (ii) K383 is the amino acid residue specifically targeted by the auto-acetylation activity of PopP2.

### K383R mutation abrogates PopP2 intermolecular autoacetylation activity

**Figure 7:**
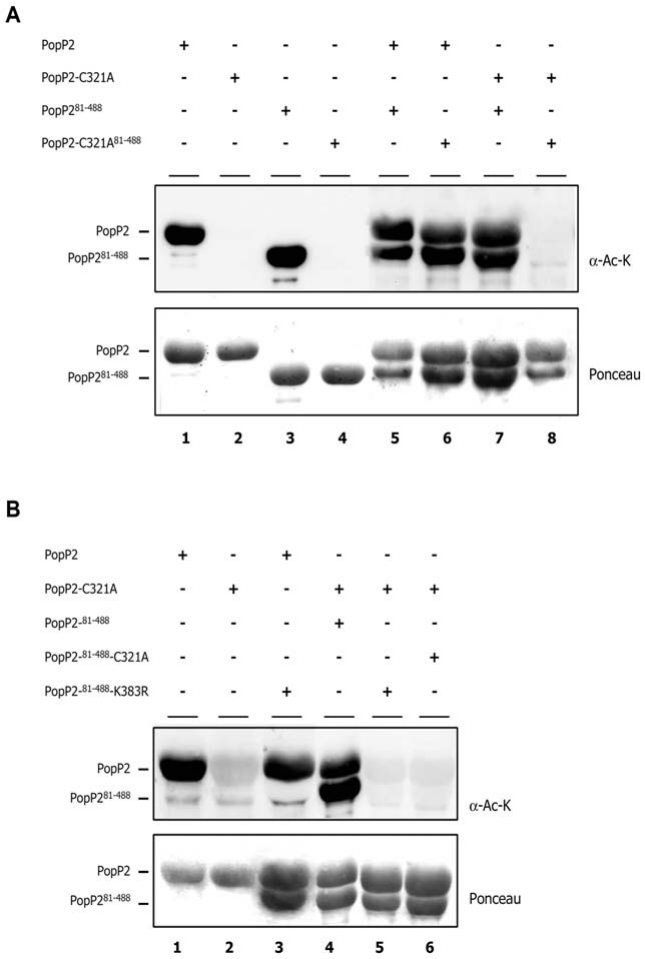
Intermolecular autoacetyl-transferase activity of PopP2 is dependent on the integrity of K383 residue. **A**: Detection of acetylated forms of GST-PopP2-^81–488^-C321A and GST-PopP2-C321A upon co-expression with GST-PopP2 and GST-PopP2-^81–488^, respectively (lane 6 and 7). **B**: K383R mutation prevents GST-PopP2 to acetylate GST-PopP2^81–488^-K383R in *trans*. The indicated protein combinations were purified from *E. coli* and their acetylation status tested by immunoblot with an α-Ac-K antibody. The position of acetylated proteins is indicated by dashes (top). GST-purified PopP2 recombinant proteins are shown after Ponceau staining (bottom).

In order to obtain further proof that acetylation of PopP2 at K383 is due to PopP2 autoacetyl-transferase activity, rather than acetylation by an endogenous *E. coli* acetyl-tranferase unable to target the PopP2-C321A or PopP2-K383 mutants, we next checked whether PopP2 is able to autoacetylate *in trans*. If this is the case, PopP2 should be able to acetylate the catalytically inactive PopP2-C321A mutant. To be able to distinguish between PopP2 and PopP2-C321A after SDS-PAGE analysis, we used a truncated form of PopP2 that lacks its first 80 amino acids (PopP2^81–488^) but retains its acetyl-transferase activity (Figure 7A, lane 3). Recombinant GST-PopP2 and GST-PopP2-C321A proteins were respectively co-expressed with active GST-PopP2^81–488^ or inactive GST-PopP2-C321A^81–488^. Purified proteins were subjected to immunoblot analysis using an α-Ac-K antibody. In the presence of active GST-PopP2^81–488^ and GST-PopP2, inactive GST-PopP2-C321A and GST-PopP2-C321A^81–488^ are respectively acetylated, demonstrating that the C321A mutation does not prevent PopP2 autoacetylation in *trans*. Although this experimental set up does not allow us to exclude the possibility that PopP2 also autoacetylates in *cis*, our data clearly demonstrate the intermolecular autoacetylation displayed by PopP2.

We next investigated whether the K383 residue, which is very likely the main acetyl-CoA acceptor site in PopP2, is required for PopP2 *trans*-autoacetylation activity. First, when active GST-PopP2 was co-expressed with a truncated form of the K383R mutant (GST-PopP2^81–488^-K383R) no GST-PopP2^81–488^-K383R acetylated form could be detected, strongly indicating that K383R mutation prevents its *trans*-acetylation by active GST-PopP2 (Figure 7B, lane 3). Second, GST-PopP2^81–488^-K383R behaves like inactive GST-PopP2^81–488^-C321A, which is not able to acetylate GST-PopP2-C321A (Figure 7B, lanes 5 and 6, respectively). Thus, despite the integrity of its catalytic triad, GST-PopP2^81–488^-K383R is impaired in its *trans*-acetylation activity. Together, our data show that K383 in PopP2 represents an acetyl-CoA binding site that is (i) targeted by autoacetylation and (ii) required for intermolecular acetylation of PopP2.

**Figure 8:**
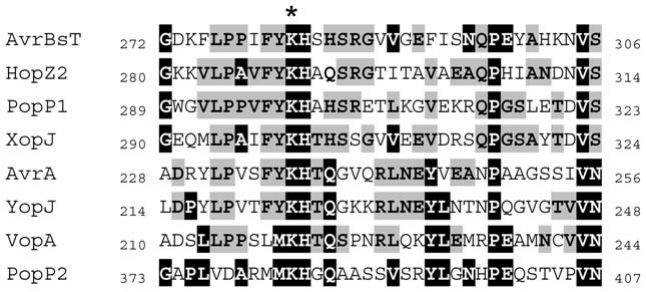
K383 residue is conserved among various members of the YopJ-like family. The region flanking the residue K383 in PopP2 is aligned with that of YopJ-like representative members from plant and animal pathogens. Common residues among all shown YopJ-like proteins, including PopP2, are shown in bold and highlighted in black. Conserved residues among shown YopJ-like proteins but not present in PopP2 are shown in bold and shadowed in grey. The position of the conserved lysine residue is indicated by a star.

Finally, the protein sequences of members of the YopJ-like family of T3Es were inspected. Figure 8 shows a sequence alignment of several YopJ-like proteins in the neighbouring region of the conserved residue K383 that is targeted by autoacetylation in PopP2. This analysis showed that the K383 residue in PopP2 is perfectly conserved among all members of the family, strongly suggesting that this residue may represent a key acetyl-CoA acceptor site essential for the *trans*-acetylation activity of PopP2 and other members of the YopJ family.

### Mutation of K383 in PopP2 abolishes RRS1-R-mediated immunity

The effect of the K383 mutation, which compromises PopP2 autoacetylation in *E. coli,* was further tested after transient expression in *N. benthamiana*. Expression of a PopP2-K383-CFP fusion was detected in the nucleus of *N. benthamiana* epidermal cells (Figure S2), confirming that this mutation does not affect nuclear targeting of the protein. In addition, co-expression of RRS1-R-YFPv and PopP2-K383-CFP led to detection of YFP fluorescence within the nucleus indicating that, as wild-type PopP2, PopP2-K383R is able to stabilize RRS1-R expression. Furthermore, significant reduction of the CFP lifetime in FRET-FLIM assays following transient co-expression of PopP2-K383-CFP and RRS1-R-YFPv, as compared to PopP2-K383-CFP expressed alone, demonstrates that PopP2-K383R also interacts with RRS1-R (Figure 4C and Table 1).

**Figure 9:**
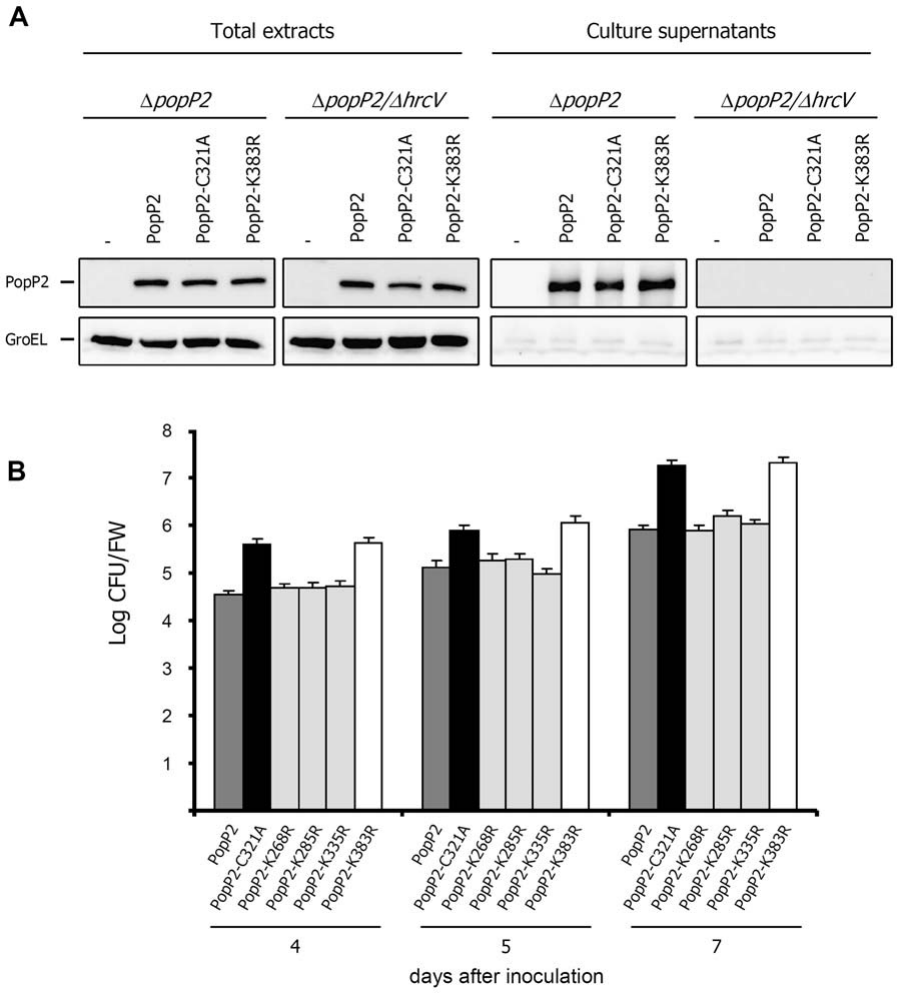
Mutation of lysine 383 in PopP2 abolishes RRS1-R-mediated immunity. **A**: PopP2, PopP2-C321A and PopP2-K383R are detected in total extracts and supernatants of *ΔpopP2* and *ΔpopP2/Δhrcv* bacterial cultures. **B**: Nd-1 plants expressing the *RRS1-R* gene were root-inoculated with the Δ*popP2* strain-expressing wild-type PopP2 (dark grey bars), PopP2-C321A (black bars), PopP2-K268R, PopP2-K285R, PopP2-K335R (all represented by clear grey bars) or PopP2-K383R (empty bars). Bacterial growth was measured 4, 5 and 7 days after inoculation. Means of colony-forming units per gram of fresh weight (cfu/gfw) were calculated from triplicates of three plants. For each time point, error bars represent standard deviation from three triplicates. Shown values are representative of two additional replicates.

In order to investigate whether PopP2 avirulence activity is compromised by mutation of K383, the PopP2-K383R mutant was next used to transform the Δ*popP2* strain of *R. solanacearum*. First, *in vitro* secretion and stability of wild-type PopP2, PopP2-C321A and PopP2-K383R were tested by immunoblot analysis. Upon incubation of the different complemented Δ*popP2* strains in secretion medium, all PopP2 variants were detected in total extracts and in culture supernatants (Figure 9A). To ensure that the signal observed from supernatant was T3SS (Type 3 Secretion System)-dependent, *popP2* constructs were also introduced into a Δ*popP2*/Δ*hrcV* strain mutated both in *popP2* and in *hrcV*, a gene coding for a conserved inner membrane component of the T3SS [48]. As expected, no signal corresponding to PopP2 was detected in culture supernatants of Δ*popP2*/Δ*hrcV* bacteria cells expressing the various PopP2 variants, demonstrating the proper secretion of PopP2, PopP2-C321A and PopP2-K383R by the T3SS. In addition, to demonstrate that no bacterial lysis had occurred, all protein samples were probed with an antibody directed against the cytoplasmic chaperonin GroEL (Figure 9A).

Growth of Δ*popP2* expressing PopP2, PopP2-C321A or PopP2-K383R was then measured in root-inoculated Nd-1 plants. Between 4 and 7 days after inoculation, Δ*popP2* strains expressing HA-tagged PopP2-C321A or PopP2-K383R reached about ten-fold higher bacterial growth rates than the Δ*popP2* strain expressing HA-tagged wild-type PopP2 (Figure 9B), showing that, as previously shown for PopP2-C321A (Figure 1B,C), PopP2-K383R is not able to trigger the RRS1-R-dependent resistance response. High bacterial growth rates observed after inoculation with the Δ*popP2* strain expressing mutant PopP2-C321A or PopP2-K383R correlated with the complete wilting of Nd-1 plants 7 days after inoculation (Figure 1B,C, Figure S3). As controls, Δ*popP2* strains expressing PopP2-K268R, PopP2-K285R or PopP2-335R were also root-inoculated in Nd-1 plants. Importantly, these PopP2 mutants were able to trigger the RRS1-R resistance response and to multiply to the same extent as wild-type PopP2 expressing strain, demonstrating that these 3 lysine residues are not required for PopP2 avirulence activity.

Together, our data show that substitution of the K383 residue in PopP2 mimics the mutation in catalytic C321, leading to inactivation of the plant immune response. These findings strongly suggest that K383 is an acetyl-CoA-binding site in PopP2, essential for RRS1-R-mediated recognition of PopP2 activity *in planta*.

## Discussion

Although the characterization of many *R-Avr* gene pairs is consistent with the gene-for-gene hypothesis [49], the underlying perception mechanism has been subject of debate for many years. Initially, the ligand-receptor model, postulating that products of *R* genes act as receptors that directly interact with the products of *Avr* genes [50], was supported by the colocalization of some Avr and R proteins, most of which encode receptor-like proteins carrying Leu-rich repeats (LRRs). However, a direct interaction between R and Avr proteins has been reported only in a few cases [8]–[10], [51], [52]. Our study represents the first report of an Avr protein (PopP2) that interacts with its matching resistance protein (RRS1-R) in living plant cells. These data are in agreement with an earlier report showing that both proteins associate in yeast cells [9]. Here, we demonstrate that both dominant *RRS1-S* and recessive *RRS1-R* gene products (i) can associate with PopP2 within the plant nucleus and (ii) are stabilized by PopP2 in this subcellular compartment. Therefore, it is tempting to propose that recessiveness of RRS1-R-mediated resistance may be due to a difference of relative RRS1 binding affinities to PopP2. Comparison of FRET-FLIM data presented in this study is not suitable to address this question since FRET efficiencies reflect only the interaction between two proteins but do not provide quantitative data. In addition, previous data showing that *RRS1-R* behaves as a dominant resistance gene in Col-0 transgenic plants are not in favour of this model. Our data strongly suggest that functionality of RRS1-R, leading to the activation of the resistance response, requires not only its interaction with PopP2 but also the perception of its enzymatic function. This recognition would be restricted to RRS1-R, the RRS1-S protein being unable to perceive PopP2 acetyl-transferase activity. This hypothesis is strengthened by the fact that PopP2 mutated in the conserved cysteine residue of its catalytic triad is unable to trigger RRS1-R-mediated immunity in Arabidopsis, despite its nuclear localization and its ability to associate with RRS1-R. Although, consistent with the gene-for-gene hypothesis, the recognition of PopP2 by RRS1-R, and not RRS1-S, is also in agreement with the guard model, according to which R proteins detect changes in host proteins after modification by pathogen-derived effectors. Alternatively, rather than a guard, RRS1-R might act as an “enabler” of PopP2 activity. In this model, targeting of host components by PopP2 with the goal of altering plant physiology would be facilitated by the RRS1-R-containing complex, allowing the effector to bind to its substrate(s). However, the outcome of this scenario would be the activation of plant defence, following perception of PopP2 enzymatic activity.

In this study, we show that PopP2 not only interacts with RRS1-R/S but is also able to specifically promote their accumulation in the nucleus. Alteration of the levels of defence-related components is a common strategy used by bacterial pathogens to suppress innate immunity in the host. For instance, AvrPphB papain-like cysteine protease from *Pseudomonas syringae* targets the Arabidopsis protein kinase PBS1 [20] whose cleavage is perceived by the resistance protein RPS5 to initiate ETI [16]. An additional T3E from *P. syringae*, AvrRpt2, is a staphopain-like cysteine protease that cleaves various host proteins including RIN4, a negative regulator of PTI, whose modification is recognized by the R protein RPS2 [21], [53], [54]. In addition to direct protein degradation resulting from protease activity, mimicking the host ubiquitination machinery represents an alternative strategy that leads to alteration of protein levels in the host. For example, the AvrPtoB E3 ligase from *P. syringae* specifically ubiquitinates the tomato protein kinase Fen and promotes its degradation in a proteasome-dependent manner [23].

To the best of our knowledge, PopP2 represents the first example of a T3E from phytopathogenic bacteria that mediates stabilization of the expression of its interacting partner(s) in the host. Previous work showed that YopJ, a well-studied bacterial T3E from the human pathogen *Y. pestis*, requires its de-ubiquitinating and acetylating activities to stabilize host components [44], [55]. In resting mammalian cells, the dimeric transcription factor NF-κB, involved in the inflammatory response to *Yersinia*, forms a complex with its inhibitor IκB. Upon its phosphorylation by the IκB kinase (IKK), IκB is ubiquitinated and subsequently degraded. YopJ promotes indirect stabilization of IκB by inhibiting IKK activity through the acetylation of a threonine residue in the activation loop of the kinase [43], thereby blocking the cell inflammatory response. In contrast, perturbation of RRS1-R proteasome-mediated degradation is unlikely to be induced by the acetyl-transferase activity of PopP2, since a catalytically inactive PopP2-C321A mutant is also able to promote accumulation of RRS1-R. Examples of eukaryotic acetyl-transferases that induce protein stabilization independent of their enzymatic activity have been previously reported. For instance, the transcriptional coactivator p300 is a histone acetyl-transferase critical for regulating gene expression in mammalian cells. The activating transcription factor 4 (ATF4) plays a crucial role in multiple stress responses and is stabilized by p300 independent of its acetyl-transferase activity [56]. The molecular mechanism involved in ATF4 stabilization by p300 is still unknown but authors hypothesized that p300, upon its interaction with ATF4, might prevent ATF4 ubiquitination or its targeting to the nuclear proteasome. Similarly, we hypothesize that PopP2, through its physical association with RRS1-R/S, may prevent RRS1-R/S ubiquitination and thus protect them from proteasome-mediated degradation. Interestingly, PopP2-mediated stabilization of RRS1-R and RRS1-S expression appears to be specific, since accumulation of the PopP2-interacting protein RD19 is not affected by PopP2. However, RRS1-R/S stabilization by PopP2 is independent of their nuclear localization. Indeed, previously published data showed that co-expression of RRS1-R/S with a PopP2 derivative deleted from its nuclear localization signal (NLS) (PopP2^95–488^), which is localized both in the nucleus and in the cytosol, leads to their detection in the same subcellular compartments [9]. Since RRS1-R has been shown to act as a negative regulator of plant resistance [57], PopP2-mediated RRS1-R stabilization may represent a bacterial strategy to promote pathogen virulence. Alternatively, RRS1-R stabilization and binding to PopP2 in the nucleus may lead to regulation of its transcriptional activity to trigger plant resistance. Anyhow, as observed in *N. benthamiana*, both C321A and K383R PopP2 mutant versions may promote RRS1-R accumulation in Arabidopsis, despite the loss of their avirulence activity. In this scenario, PopP2-mediated stabilization of RRS1-R expression would not be sufficient *per se* for activation of the immune response.

A number of T3ES exert their function by covalently modifying target proteins in the host cell. These covalent modifications are generally reversible and presumably aimed at modulating cellular functions by transiently altering the activity of pathogen cellular targets. In plants, T3Es have been previously found to modulate postranslational modifications (PTMs) in host proteins, including phosphorylation, SUMOylation and ubiquitination [19]. Here, we describe acetylation as a novel PTM displayed by a T3E from a plant pathogenic bacterium. Acetylation is a major PTM first identified on lysine residues from histones [58]. Addition of acetyl groups to the lysine residues of histone tails facilitates access of transcription factors to DNA by disrupting higher-order packaging of the chromatin [59] and also by neutralizing the positive charge of the histone proteins, which reduces the affinity of histones for DNA [60]. Acetylation also impairs the ability of the lysine side chain to form hydrogen bonds thereby enhancing specific or inhibiting non-specific DNA-binding activities of transcription factors [61]–[63]. In addition, acetylation forms docking sites for recruitment of transcriptional coactivators [64]. Several bacterial effectors have been found to affect host transcription. For example, members of the AvrBs3 family from the phytopathogenic bacterium *Xanthomonas campestris*, also referred to as TAL (Transcription Activator-Like) effectors, are targeted to the plant cell nucleus where they directly induce expression of plant genes [25], [26]. In addition, the SUMO-protease T3E XopD from *X. campestris* is also targeted to the nucleus of host cells and functions as a transcriptional repressor, resulting in suppression of host defence responses through an unknown molecular mechanism that may involve deSUMOylation of transcription factors or chromatin remodelling, for example [65]. Likewise, we propose that PopP2 autoacetylation and/or acetylation of its interacting partner(s), perhaps RRS1-R, may affect gene transcription in host cells.

Similar to phosphorylation, which is described to target kinases and phosphatases, acetyl-transferases can be acetylated *via* either intra (*cis*) or intermolecular (*trans*) mechanisms. Although it is possible that PopP2 autoacetylation additionally occurs in *cis,* our data clearly demonstrate the intermolecular autoacetylation of PopP2 on a lysine residue that is well conserved among all members of the YopJ-like family. It is thus tempting to speculate on the biological significance of the acetylation of this particular residue. Indeed, YopJ is predicted to use a two-substrate ping-pong mechanism whereby acetyl-coenzyme A (acetyl-CoA; the first substrate) interacts with the enzyme to form a high-energy acyl-enzyme intermediate, that is attacked by the second substrate resulting in a modified product [44], [45]. A previous study suggested that, in addition to the conserved catalytic core essential for acetyl-transferase activity, each member of the YopJ family, whether from a plant or animal pathogen, presents a conserved site, possibly located at the C terminus, that binds acetyl-CoA [45]. However, the identity of this acetyl-CoA-binding site and the reason(s) behind its conservation within the YopJ protein family are still unknown. The observations that (i) PopP2 autoacetylates in K383, a key residue that is required for its intermolecular acetylation activity, (ii) K383 in PopP2 is conserved among all members of the YopJ family of T3Es, and (iii) despite the presence of an integral catalytic triad, a PopP2-K383R mutant is no longer recognized by RRS1-R-expressing Arabidopsis plants, strongly support the idea that this lysine residue represents a good candidate to be the unknown acetyl-CoA binding site within the YopJ family. As a consequence of the mutation of K383 that prevents PopP2 to bind acetyl-CoA, PopP2 would be impaired not only in its autoacetyl-transferase activity *in planta* but also in its ability to acetylate its putative host substrate(s).

At present, it is difficult to distinguish between the two following hypotheses: is PopP2 autoacetylation and/or acetylation of its host substrate(s) required to trigger the RRS1-R resistance response? Indeed, both possibilities, which are not mutually exclusive, are consistent with the loss of avirulence activity of the PopP2-K383R mutant that behaves like the catalytic (acetyl-transferase defective) mutant PopP2-C321A, after inoculation of RRS1-R-expressing plants. According to the guard model [12], RRS1-R may survey the acetylation state of host components targeted by PopP2. The identified PopP2-interacting proteins RRS1-R and the vacuolar RD19 cysteine protease [46] are potential substrates that may be targeted by the acetyl-transferase activity of PopP2. However, despite several attempts, immunoprecipitation experiments, followed by Western blot analysis with α-Ac-K antibody, did not allow detection of any acetylated form of RRS1-S, RRS1-R or RD19 upon co-expression with PopP2. Indeed, additional plant or bacterial components may be required for PopP2 acetylation of its substrate(s). Alternatively, as previously described for YopJ, which is able to acetylate target proteins on serine and threonine residues [45], PopP2 acetyl-transferase activity may target additional amino acid residues other than lysine, which would be undetectable under our experimental conditions.

Future work will address these questions as well as the acetylated state of PopP2 *in planta* and whether its activity may be modulated by the presence of putative plant inhibiting and/or activating co-factors. Indeed, rather than acting as substrates, PopP2-interacting proteins, through their physical association with PopP2, might also modulate its enzymatic activity. According to this hypothesis, it is tempting to hypothesize that RRS1-R, unlike RRS1-S, might potentiate PopP2 enzymatic function, thereby leading to its recognition and activation of the resistance response. Identification of host proteins targeted by PopP2 activity and/or involved in its regulation will significantly contribute to the understanding of the molecular role(s) played by protein acetylation during plant innate immunity.

## Materials and Methods

All experiments reported in this article were performed at least three times with similar results.

### Plasmids, strains and DNA

Plasmids used in this study were constructed by Gateway technology (GW; Invitrogen) following the instructions of the manufacturer. PopP2 mutants were generated from pENTR-PopP2 [9] by a two-step PCR-based site-directed mutagenesis using PrimeStar HS DNA polymerase from Takara Bio Inc. (Otsu, Japan) to introduce the following nucleotide substitutions: K268R: codon 268 AAG to *CGG* (K268R-F: AT ATT CGC *CGG* GAC GCC TCT GGT ACG AGC GTG ATC, *K268R-R: GA GGC GTC CCG GCG AAT ATC TGC GGC TCT GGT);* K285R: codon 285 AAA to *AGA* (K285R-F: C CTC CGA AAG GAA *AGA* GAT GAA AGC GCG TAC GTC GA, *K285R-R: C ATC TCT TTC CTT TCG GAG GGG ATC GAC AAC G);* K335R: codon 335 AAG to *CGG* (K335R-F: C AAG ATG CAT GAC *CGG* GAC GAC GCG TTT GC, *K335R-R: C GTC CCG GTC ATG CAT CTT GAG TGC AAG TGA);* C321A: codon 321 TGC to *GCC* (C321A-F: C TTC TTC GAT *GCC* CGG ATA CTC TCC CTG TCA CT, *C321A-R: GAG TAT CCG GGC ATC GAA GAA GGA CTT CTG A);* K383R: codon 383 AAA to *CGG* (K383R-F: GT ATG ATG *CGG* CAT GGT CAA GCC GCA, *K383R-R: C TTG ACC ATG CCG CAT CAT ACG GGC GT).* Second step PCR was performed using *popP2*-specific primers (AttB1-popP2-F: GGGG ACA AGT TTG TAC AAA AAA GCA GGC TTA ATG AAG GTC AGT AGC GCA; AttB2-popP2-R: GGGG ACC ACT TTG TAC AAG AAA GCT GGG TCG TTG GTA TCC AAT AGG GAA TCC). pENTR-PopP2 truncated clones (PopP2^81–488^, PopP2-C321A^81–488^, PopP2-K383R^81–488^) were generated by PCR performed on corresponding pENTR-PopP2 full length clones (AttB1-PopP2-81F: GGGG ACA AGT TTG TAC AAA AAA GCA GGC TTA ATG CAT GTG CCA CTC CTA GAC A and AttB2-PopP2-R). Gateway PCR products flanked by *attB* sites were recombined into pDONR207 vector (Invitrogen) *via* a BP reaction to create corresponding entry clones with *attL* sites. Relative pENTR-PopP2 mutants were recombined into appropriate destination vectors *via* an LR reaction. pENTR-RRS1-S, pENTR-RRS1-R and pENTR-RD19 (pDONR207 vector backbone) used in this study have been previously described [9], [46]. *In planta* expression of the various protein fusions described in this study was performed using Gateway compatible pAM-PAT-P35S-GW binary vectors [46] to allow C-terminal protein tagging with the following epitopes: CFP, YFPv, 3x-Flag, 3x-HA and c-myc.

### Bacterial inoculations

Plant root inoculations and bacterial internal growth curves were performed as previously described [37]. Plant phenotypic responses were scored daily, using a disease-index scale ranging from 0 to 4, according to the percentage of wilted leaves (0  =  no wilt, 1 = 1 to 25%, 2 = 26 to 50%, 3 = 51 to 75%, 4  = >75%).

### Fluorescence microscopy

The CFP and YFP fluorescence in *N. benthamiana* leaves was analyzed with a Confocal Laser Scanning Microscope (TCS SP2-SE, Leica, Germany) using a 63× water immersion objective lens (numerical aperture 1.20, PL APO). CFP fluorescence was excited with the 458-nm ray line of the argon laser and recorded in one of the confocal channels in the 465- to 520-nm emission range. YFP fluorescence was excited with the 514-nm line ray of the argon laser and detected in the range between 520 and 575 nm. Images were acquired in the sequential mode using Leica LCS software (version 2.61).

### Fluorescence lifetime microscopy and data analysis

Fluorescence lifetime of the donor was experimentally measured in the presence and absence of the acceptor. FRET efficiency (E) was calculated by comparing the lifetime of the donor in the presence (τ_DA_) or abscence (τ_D_) of the acceptor: E = 1−(τ_DA_)/(τ_D_). FRET-FLIM measurements were performed using a multiphoton FLIM system coupled to a streak camera (Krishnan et al., 2003). The light source was a mode-locked Ti:sapphire laser (Tsunami, model 3941, Spectra-Physics, USA), pumped by a 10 W diode laser (Millennia Pro, Spectra-Physics), delivering ultrafast femtosecond pulses with a fundamental frequency of 80 MHz. A pulsepicker (model 3980, Spectra-Physics) was used to reduce the repetition rate to 2 MHz. All the experiments reported in this work were carried out at λ = 820 nm, the optimal wavelength to excite CFP in multiphoton mode while minimizing the excitation of YFP (Chen and Periasamy, 2004). The power delivered at the entrance of the FLIM optics was 14 mW. All images were acquired with a 60× oil immersion lens (Plan Apo 1.4 numerical aperture, IR) mounted on an inverted microscope (Eclipse TE2000E, Nikon, Japan) coupled to the FLIM system. The fluorescence emission was directed back out into the detection unit through a short pass filter (λ<750 nm). The FLIM unit was composed of a streak camera (Streakscope C4334, Hamamatsu Photonics, Japan) coupled to a fast and high-sensitivity CCD camera (model C8800-53C, Hamamatsu). For each nucleus, average fluorescence decay profiles were plotted and lifetimes were estimated by fitting data with tri-exponential function using a non-linear least-squares estimation procedure with Origin 7.5 software (OriginLab, Northampton USA).

### Transient assays

For *Agrobacterium*-mediated *Nicotiana benthamiana* leaf transformations, the relevant GV3101 strains were grown in Luria-Bertani liquid medium containing 100 µg mL-1 rifampicin, 25 µg mL-1 gentamicin and 25 µg mL-1 carbenicillin at 28°C for 24 h before use. Bacteria were harvested and resuspended in infiltration medium (10 mM MES pH 5.6, 10 mM MgCl_2_, 150 µM acetosyringone) to an OD_600nm_ of 0.5 and incubated for 2 h at room temperature before leaf infiltration. The infiltrated plants were incubated for 36 h in growth chambers under controlled conditions [46]. *Arabidopsis* transient assays were performed as previously described [66]. Four day-old seedlings were infiltrated with appropriate *A. tumefaciens* strains and harvested 3 days after infiltration. MG132 treatment (100 µM) was performed 24 h after agroinfiltration. Leaf disks were harvested 12 h later and grounded in Laemmli buffer.

### Protein analyses

For bacterial overexpression, *popP2* wild-type and *popP2* mutant versions were recombined into a pGEX-GWY vector by LR recombination (Invitrogen). Relative GST-PopP2 constructs were expressed in *Escherichia coli* Rosetta DE3 cells (Novagen). The intermolecular acetylation assay was performed by co-expressing truncated PopP2 variants (PopP2^81–488^, PopP2-C321A^81–488^ and PopP2-K383R^81–488^) with either PopP2 or PopP2-C321A in Rosetta DE3 cells as GST fusion proteins. A fragment containing the *GST-PopP2* or *GST-PopP2-C321A* coding sequences under the control of a *tac* promoter was introduced into the pGEX-GWY vector to generate pGEX-GWY-GST-PopP2 and pGEX-GWY-GST-PopP2-C321A destination vectors, respectively. Relative pENTR-PopP2 truncated clones (PopP2^81–488^, PopP2-C321A^81–488^, PopP2-K383R^81–488^) were recombined into pGEX-GWY-GST-PopP2 or pGEX-GWY-GST-PopP2-C321A vectors by LR recombination. Cells were grown at 37°C to an OD_600nm_ of 0.6 in LB medium (50 µg mL-1 carbenicillin and 30 µg mL-1 chloramphenicol) and induced with 400 µM isopropyl-βthiogalactopyranoside (Roche Applied Science) for 3 h at 28°C. Before lysis, pelleted cells were concentrated 10 times in PBS [phosphate-buffered saline (pH 8.0)] supplemented with 1 mM phenylmethylsulfonyl fluoride (PMSF, Sigma) and 10 mM Sodium butyrate. GST purifications were performed using Glutathione Sepharose (GE Healthcare) according to the instructions of the manufacturer. For a given experiment, between 3 and 5 µg of purified proteins were used for Western blot analysis.

### Immunodetection

Equal amounts of protein were loaded onto a sodium dodecyl sulfate-polyacrylamide gel electrophoresis (SDS-PAGE), followed by electrophoresis and transfer to Protran BA85 nitrocellulose membranes (Whatman, Germany). Transferred proteins were visualized by Ponceau S red staining. Plant protein samples obtained from both *N. benthamiana* leaves (4 discs of 8 mm diameter harvested 24 to 36 hours post-infiltration) and Arabidopsis (20 seedlings), were homogenized in 250 µL of Laemmli loading buffer. Antibodies used for Western blotting were anti-Flag-HRP (M2, Sigma, dilution 1∶5000), anti-HA-HRP (3F10, Roche Applied Science, dilution 1∶5000), anti-myc-HRP (9E10, Roche Applied Science, dilution 1∶3000), monoclonal mouse anti-acetylated-Lysine antibody (Ac-K-103, Cell Signaling Technology; dilution 1∶2000), polyclonal rabbit anti-GroEL (Stressgen Biotechnologies Corporation, dilution 1∶20000), goat anti-mouse and anti-rabbit IgG-HRP antibodies (Santa Cruz, dilution 1∶10000).

### Nano-LC-MS/MS analysis

GST affinity purified proteins were migrated on an SDS-PAGE. The gel band samples were washed several times by incubation in 25 mM NH_4_HCO_3_ for 15 min and then in 50% (v/v) acetonitrile containing 25 mM NH_4_HCO_3_ for 15 min. 0.15 µg of modified trypsin (Promega, sequencing grade) in 25 mM NH_4_HCO_3_ was added to the dehydrated gel spots for an overnight incubation at 37°C. Peptides were then extracted from gel pieces in three 15 min sequential extraction steps in 30 µL of 50% acetonitrile, 30 µL of 5% formic acid and finally 30 µL of 100% acetonitrile. The pooled supernatants were then dried under vacuum. For nano-LC-MS/MS analysis, the dried extracted peptides were resuspended in 30 µl of water containing 2.5% acetonitrile and 0.1 fluoroacetic acid. A nano-LC-MS/MS analysis was then performed (Ultimate 3000, Dionex and LTQ-Orbitrap XL, Thermo Fischer Scientific). The method consisted in a 60-minute gradient at a flow rate of 300 nL/min using a gradient from two solvents: A (5% acetonitrile and 0.1% formic acid in water) and B (80% acetonitrile and 0.08% formic acid in water). The LC system includes a 300 µm×5 mm PepMap C18 precolumn and a 75 µm×150 mm C18 column (PepMap C18 phase). MS and MS/MS data were acquired using Xcalibur (Thermo Fischer Scientific). The raw data were automatically processed through Mascot Daemon software (Matrix Science) and the MS/MS spectra were searched against the SwissProt/Trembl database using trypsin as the specific enzyme. Peptide modifications allowed during the searches were: N-acetyl (N-ter protein), oxidation (M), dioxidation (M), trioxidation (C) and K-acetylation.

### Complementation analyses of the Δ*popP2* strain of *R. solanacearum*

*popP2* allelic sequences were introduced by LR recombination in pLAFR6-P2GFH (*popP2*promoter::GW::3x-HA)vector that allows the expression of PopP2 tagged in the C-terminus end with 3x-HA epitope tag. To drive the expression of epitope tagged-PopP2 variants, a fragment that encompass 384 bp of *PopP2* promoter sequence (from ATG, amplified from GMI1000 genomic DNA) was used. Δ*hrcV* mutation was introduced into *ΔpopP2* strain by natural transformation of bacteria grown in MMG medium supplemented with glycerol (20 g L-1) with 3–5 µg of Δ*hrcV* genomic DNA. The derived pLAFR-P2GFH plasmids were introduced into *R. solanacearum ΔpopP2* (GRS100, [9]) and *ΔpopP2/Δhrcv* (this study) strains by electroporation and selected on 10 µg mL-1 tetracyclin and 15 µg mL-1 gentamicin. Production of *R. solanacearum* concentrated supernatants from 2 mL of culture was performed as previously described [67]. Total extracts were obtained from the lysis of pelleted cells (2 mL) in Bugbuster protein extraction reagent (Novagen) following the instructions of the manufacturer.

### Accession numbers

Sequence data from this article can be found in the Arabidopsis Genome Initiative or GenBank/EMBL data libraries under the following accession numbers: AvrA (AAB83970), AvrBst (AAD39255), HopZ2 (ABK13722), PopP1 (CAF32331), PopP2 (CAD14570), RD19 (At4g39090), RRS1-S (At5G45260), RRS1-R (HQ170631), VopA (AAT08443), XopJ (YP_363887), YopJ (NP_395205).

## Supporting Information

Figure S1 Accumulation of RD19-3xFlag is not affected by co-expression with PopP2. *Agrobacterium*-mediated transient expression of RD19-3x-Flag alone (-) or with PopP2-3x-HA in *N. benthamiana* epidermal cells. Protein samples were harvested 36 h after infiltration and analyzed using the indicated antibodies. Rubisco is shown as loading control (bottom).
(0.18 MB TIF)

Figure S2 PopP2-K383R-CFP is targeted to the nucleus of Arabidopsis cells. Confocal images of Arabidopsis epidermal cells, 72 h after *Agrobacterium*-mediated transient expression of P35S:PopP2-K383R-CFP. Bar =  20 µm.
(0.89 MB TIF)

Figure S3 Mutation of lysine 383 in PopP2 leads to loss of PopP2 avirulence activity on RRS1-R expressing plants. Phenotypic responses of Nd-1 (RRS1-R) Arabidopsis plants 8 days after inoculation with Δ*popP2* strain expressing wild-type PopP2 or mutant PopP2-K383R.
(1.00 MB TIF)

Table S1 Liquid chromatography/tandem mass spectrometry (LC-MS/MS) analysis of GST-PopP2. Legend corresponding to Tables S1, S2 and S3: ^(a)^Mass: molecular mass of the protein of interest (kDa), ^(b)^Coverage: percentage protein sequence coverage, ^(c)^#peptides: number of identified peptides related to the protein of interest, ^(d)^emPAI: exponentially modified Protein Abundance Index, calculated with MASCOT, ^(e)^SC (relevant+duplicated): spectral count (unique and duplicate), ^(f)^query: spectral number, ^(g)^observed: mass obtained by the mass spec, ^(h)^Mr(expt): expected molecular weight of the considered peptide, ^(i)^Mr (calc): molecular weight of the considered peptide, ^(j)^delta: difference between Mr (calc) and Mr (expt) in ppm, ^(k)^miss: number of missed cleavage, ^(l)^score: Mascot score for the identified peptide, ^(m)^start/stop: position of the first (start) or the last (stop) residue of the considered peptide within the sequence of the protein of interest, ^(n)^Sequence: sequence of the considered peptide, ^(o)^modification: modification identified in the peptide, ^(p)^R.T: Retention Time (sec.). Lines corresponding to acetylated peptides are shaded in grey.
(0.99 MB RTF)

Table S2 Liquid chromatography/tandem mass spectrometry (LC-MS/MS) analysis of GST-PopP2-C321A.
(0.71 MB RTF)

Table S3 Liquid chromatography/tandem mass spectrometry (LC-MS/MS) analysis of GST-PopP2-K383R.
(0.94 MB RTF)
