# Correction: Autoacetylation of the *Ralstonia solanacearum* Effector PopP2 Targets a Lysine Residue Essential for RRS1-R-Mediated Immunity in Arabidopsis

**DOI:** 10.1371/journal.ppat.1010368

**Published:** 2022-03-02

**Authors:** Céline Tasset, Maud Bernoux, Alain Jauneau, Cécile Pouzet, Christian Brière, Sylvie Kieffer-Jacquinod, Susana Rivas, Yves Marco, Laurent Deslandes

After this article [1] was published, concerns were raised about results reported in Figures 5 and 7.

- In Figure 5, the Rubisco panels for RRS1-R-3x-Flag and RRS1-S-3x-Flag experiments appear similar. The authors also commented that errors were made in assembling Figure 5A and that the α-HA and α-Flag blots shown in Figure 5A do not report experiments using the indicated samples. They provided an updated version of Figure 5A and its legend in which the Figure 5A results were replaced with the correct data from the original experiment (S1 File, slide 1). The replacement data support the figure’s conclusion that there is an increased accumulation of RRS1-R and RRS1-S protein levels in the presence of PopP2 and its catalytic mutant. However, the α-HA blot in the updated Figure 5A does not include data for the control lanes (lanes 1, 4).
  S1 File (slides 2–5) includes the raw image data underlying the corrected figure, raw images supporting the left panel in the original version of Figure 5A, and other experimental data to support this figure’s conclusion. Information about mutants used in the additional supporting experiments are in S2 File. The data supporting the right half of the original Figure 5A are no longer available.
- In Fig 7, when levels are adjusted there appear to be vertical discontinuities in background between lanes 4 and 5 in the α-Ac-K blot of panel A, and between lanes 3 and 4 of the α-Ac-K blot of panel B. The authors clarified that Fig 7 was generated by combining results from three replicate experiments that were carried out under identical conditions as described in the Materials and Methods [1] (see S3 File). Lanes 1–4 of the α-Ac-K panel in Fig 7A were obtained in one replicate; lanes 5–8 of the α-Ac-K and Ponceau panels of Fig 7A and lanes 1–3 of α-Ac-K and Ponceau panels in Fig 7B were obtained in a second replicate; lanes 4–6 of the α-Ac-K and Ponceau panels in Fig 7B were obtained in a third replicate. In addition, the data shown in lanes 1–3 of the Fig 7B blots include data obtained on parallel blots from the same experimental replicate that were imaged at the same exposure: lane 3 data were obtained using a different blot than lanes 1 and 2. The authors apologize for combining data inappropriately in the original figure. The raw image data for the three experiments reported in Fig 7 are in S4 File.
  The original data underlying the Ponceau panel in lanes 1–4 of Fig 7A are no longer available, but the authors provided data from an independent replicate experiment (S5 File) which confirm the result that PopP2 lacking its first 80 residues (PopP2^81-488^) retains its acetyl-transferase activity, and this activity is disrupted in the presence of a C321A mutation.
  An updated Fig 7 is provided here. In the revised version, the above issues have been addressed by reporting data from different replicates in different figure panels, clearly indicating where an image was spliced in preparing the figure, and replacing the Fig 7A results with the replication results for which supporting data are in S6 File.

The second paragraph of the “K383R mutation abrogates PopP2 intermolecular autoacetylation activity” Results section is revised as follows so that the figure citations align with the updated version of Fig 7:

We next investigated whether the K383 residue, which is very likely the main acetyl-CoA acceptor site in PopP2, is required for PopP2 *trans*-autoacetylation activity. First, when active GST-PopP2 was co-expressed with a truncated form of the K383R mutant (GST-PopP2^81–488^-K383R) no GST-PopP2^81–488^-K383R acetylated form could be detected, strongly indicating that K383R mutation prevents its *trans*-acetylation by active GST-PopP2 (Fig 7B, lane 5). Second, GST-PopP2^81–488^-K383R behaves like inactive GST-PopP2^81–488^-C321A, which is not able to acetylate GST-PopP2-C321A (Fig 7C, lanes 2 and 3, respectively). Thus, despite the integrity of its catalytic triad, GST-PopP2^81–488^-K383R is impaired in its *trans*-acetylation activity. Together, our data show that K383 in PopP2 represents an acetyl-CoA binding site that is (i) targeted by autoacetylation and (ii) required for intermolecular acetylation of PopP2.

The original data supporting Figures 2D, 3D, 3E, and S1 are no longer available. The data underlying other results reported in [1] are available upon request from the authors.

The *PLOS Pathogens* Editors confirmed that the data provided for Figure 5 and Fig 7 support the results reported in the article.

**Fig 7:**
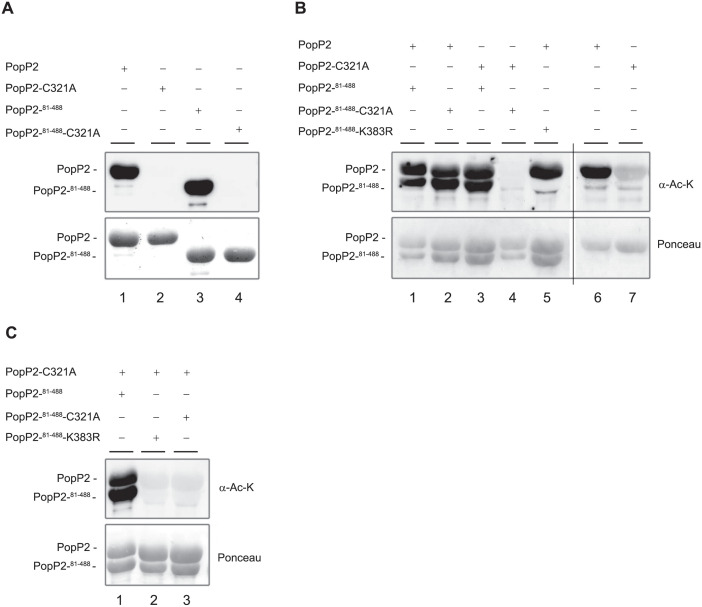
Intermolecular autoacetyl-transferase activity of PopP2 is dependent on the integrity of K383 residue. **A**: A truncated form of PopP2 that lacks its first 80 amino acids (PopP2-^81-488^) retains its acetyltransferase activity. **B**: Detection of acetylated forms of GST-PopP2-C321A and GST-PopP2-C321A-^81-488^ upon co-expression with GST-PopP2-^81-488^ and GST-PopP2, respectively (lanes 2 and 3). K383R mutation prevents GST-PopP2 to acetylate GST-PopP2-^81-488^-K383R in *trans* (lane 5). The black line indicates image splicing (lanes 1 to 5 and lanes 6–7 are from the same experiment consisting in two membranes). **C**: Detection of acetylated forms of GST-PopP2-C321A upon co-expression with GST-PopP2-^81-488^ (lane 1) but not with GST-PopP2-C321A^81-488^ (lane 2) or GST-PopP2-^81-488^-K383R (lane 3). The indicated protein combinations were purified from *E*. *coli* and their acetylation status tested by immunoblot with an α-Ac-K antibody. The position of acetylated proteins is indicated by dashes (top). GST-purified PopP2 recombinant proteins are shown after Ponceau staining (bottom). A, B and C represent three independent experiments carried out under identical conditions.

## Supporting information

S1 File Corrected Figure 5A and supporting data. (PPTX)

S2 File Legend listing reagents used in the experiment shown in Slide 4 of S1 File. (XLS)

S3 File Diagram showing how Figure 7 in [1] was compiled from the raw image data. (PPTX)

S4 File Raw image data underlying Fig 7. (ZIP)

S5 File Replicate data provided in support of Fig 7A. (TIF)
