# Supplementary figures and images for: Autoacetylation of the Ralstonia solanacearum Effector PopP2 Targets a Lysine Residue Essential for RRS1-R-Mediated Immunity in Arabidopsis
Source: PLoS Pathog. 2010 Nov 18;6(11):e1001202. doi: 10.1371/journal.ppat.1001202 (PMC2987829; doi:10.1371/journal.ppat.1001202)

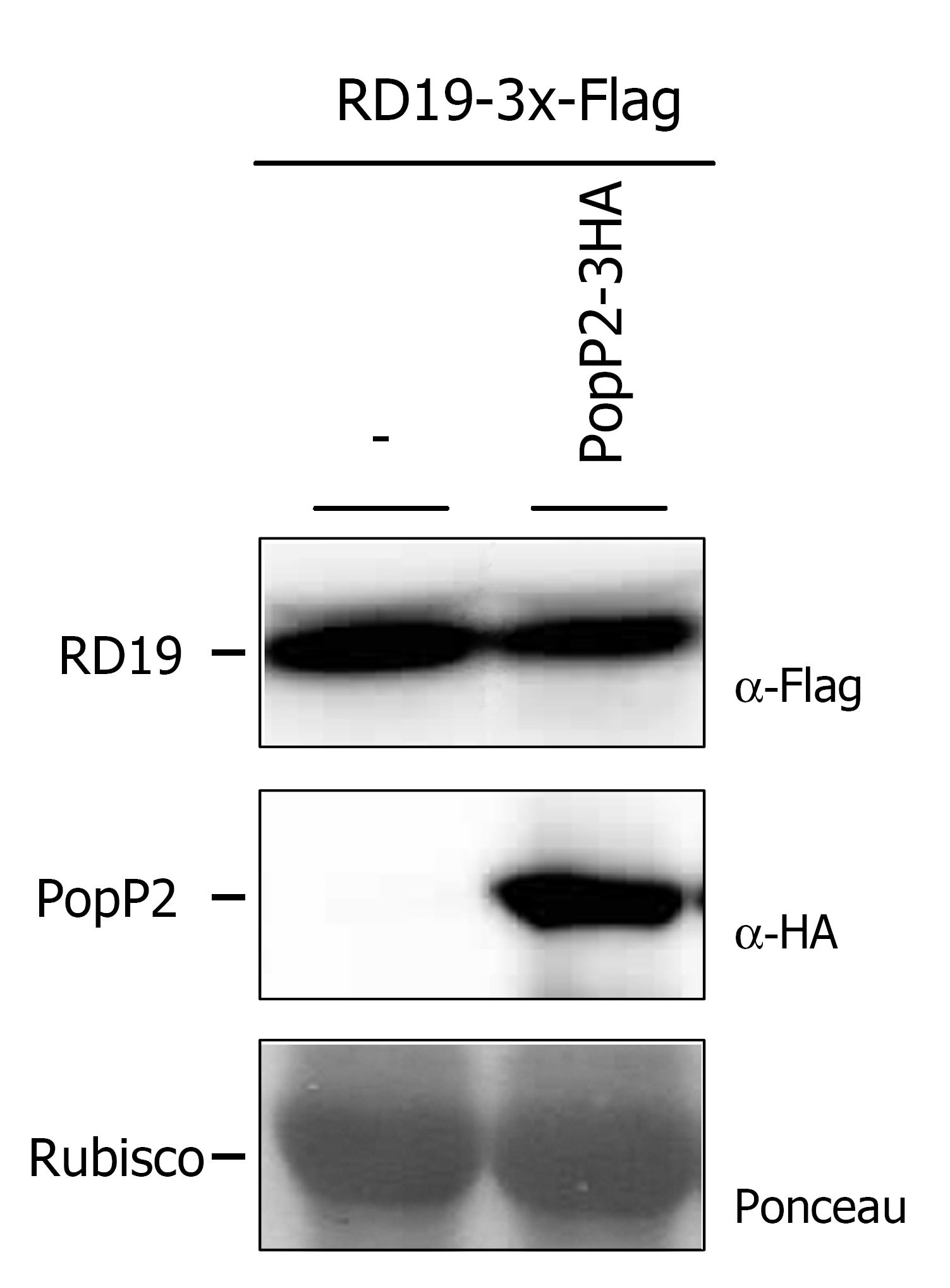

Supplement: Figure S1 — Accumulation of RD19-3xFlag is not affected by co-expression with PopP2. Agrobacterium-mediated transient expression of RD19-3x-Flag alone (-) or with PopP2-3x-HA in N. benthamiana epidermal cells. Protein samples were harvested 36 h after infiltration and analyzed using the indicated antibodies. Rubisco is shown as loading control (bottom). (0.18 MB TIF) [file ppat.1001202.s001.tif]

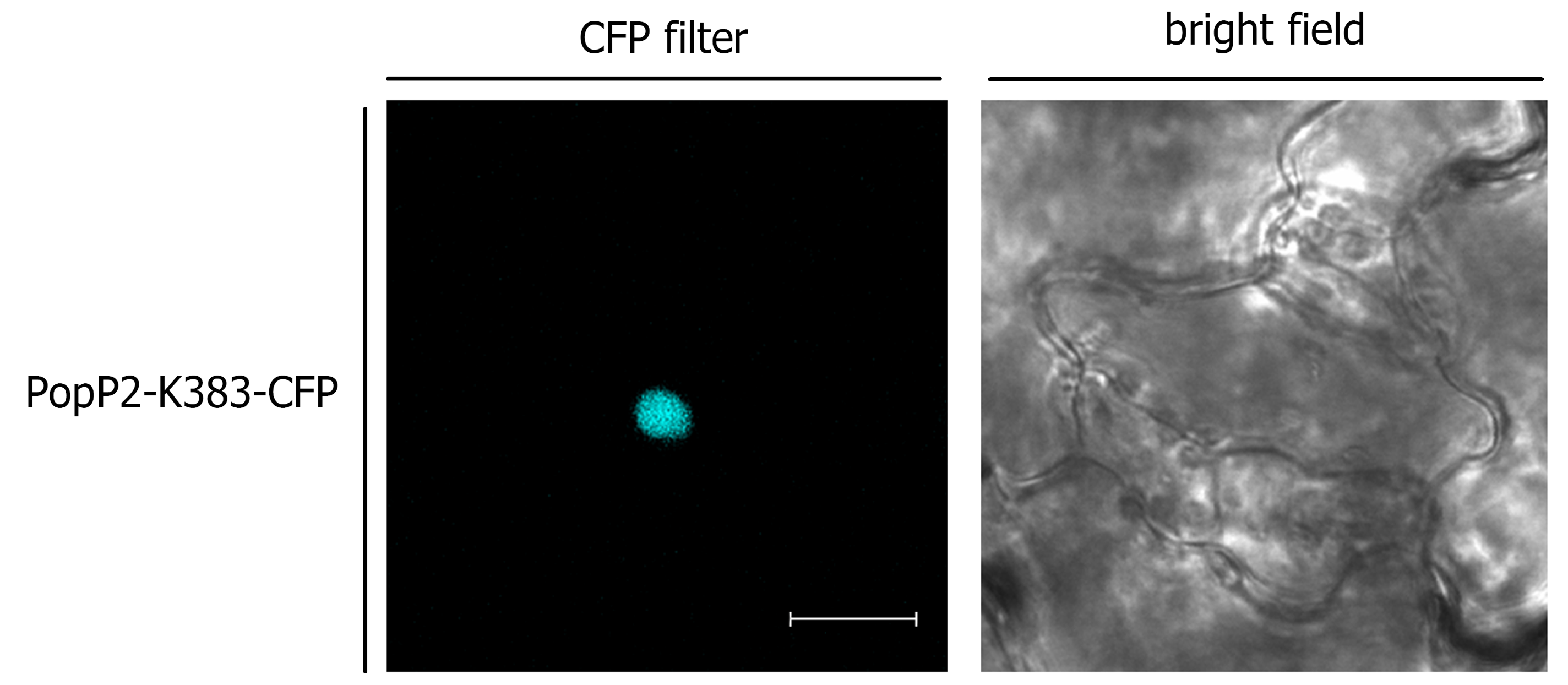

Supplement: Figure S2 — PopP2-K383R-CFP is targeted to the nucleus of Arabidopsis cells. Confocal images of Arabidopsis epidermal cells, 72 h after Agrobacterium-mediated transient expression of P35S:PopP2-K383R-CFP. Bar = 20 µm. (0.89 MB TIF) [file ppat.1001202.s002.tif]

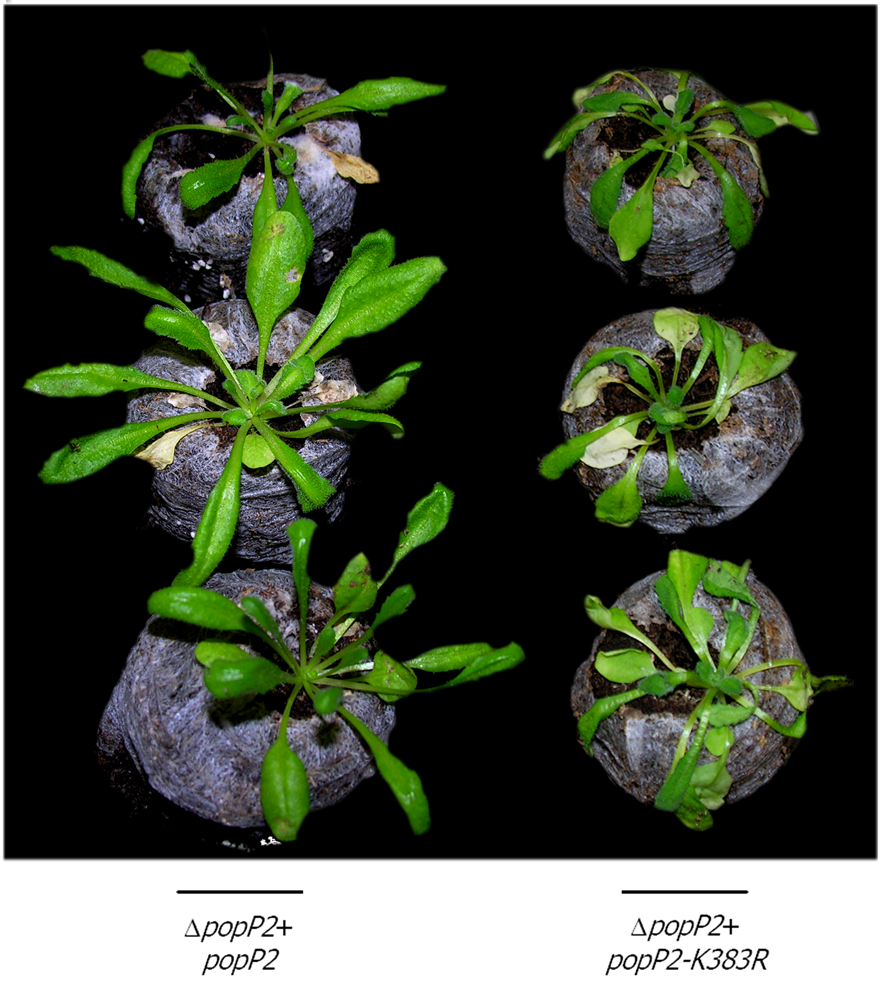

Supplement: Figure S3 — Mutation of lysine 383 in PopP2 leads to loss of PopP2 avirulence activity on RRS1-R expressing plants. Phenotypic responses of Nd-1 (RRS1-R) Arabidopsis plants 8 days after inoculation with ΔpopP2 strain expressing wild-type PopP2 or mutant PopP2-K383R. (1.00 MB TIF) [file ppat.1001202.s003.tif]
