# Supplementary material for: Autoacetylation of the Ralstonia solanacearum Effector PopP2 Targets a Lysine Residue Essential for RRS1-R-Mediated Immunity in Arabidopsis
Source: PLoS Pathog. 2010 Nov 18;6(11):e1001202. doi: 10.1371/journal.ppat.1001202 (PMC2987829; doi:10.1371/journal.ppat.1001202)
Supplement: Table S1 — Liquid chromatography/tandem mass spectrometry (LC-MS/MS) analysis of GST-PopP2. Legend corresponding to Tables S1, S2 and S3: (a)Mass: molecular mass of the protein of interest (kDa), (b)Coverage: percentage protein sequence coverage, (c)#peptides: number of identified peptides related to the protein of interest, (d)emPAI: exponentially modified Protein Abundance Index, calculated with MASCOT, (e)SC (relevant+duplicated): spectral count (unique and duplicate), (f)query: spectral number, (g)observed: mass obtained by the mass spec, (h)Mr(expt): expected molecular weight of the considered peptide, (i)Mr (calc): molecular weight of the considered peptide, (j)delta: difference between Mr (calc) and Mr (expt) in ppm, (k)miss: number of missed cleavage, (l)score: Mascot score for the identified peptide, (m)start/stop: position of the first (start) or the last (stop) residue of the considered peptide within the sequence of the protein of interest, (n)Sequence: sequence of the considered peptide, (o)modification: modification identified in the peptide, (p)R.T: Retention Time (sec.). Lines corresponding to acetylated peptides are shaded in grey. (0.99 MB RTF) [file ppat.1001202.s004.rtf]

Description	accession	(a) Mass	(b) Coverage	(c) #peptides	(d) emPAI	(e) SC (relevant+duplicated)							
PopP2	Q8Y125	52819,67	86,25	42	658,33	307							
(f) Query	(g) Observed	(h)Mr (expt)	(i) Mr (calc)	(j) delta	(k) miss	(l) score	(m) start	stop	(n) sequence		(o) modifications	(p) R.T.	
1803	787,90	1573,79	1573,79	0,07	0	78,25	461	475	AASYVNSAPPPVVMR	M	Oxidation (M) [14]	1453,99	
1758	779,90	1557,79	1557,80	-2,98	0	30,51	461	475	AASYVNSAPPPVVMR	M		1656,44	
1757	779,90	1557,79	1557,80	-3,24	0	53,74	461	475	AASYVNSAPPPVVMR	M		1720,88	
1792	787,90	1573,79	1573,79	-4,29	0	75,29	461	475	AASYVNSAPPPVVMR	M	Oxidation (M) [14]	1496,21	
1793	525,60	1573,79	1573,79	-4,28	0	33,17	461	475	AASYVNSAPPPVVMR	M	Oxidation (M) [14]	1524,25	
1794	787,90	1573,79	1573,79	-3,76	0	36,22	461	475	AASYVNSAPPPVVMR	M	Oxidation (M) [14]	1611,09	
1760	779,91	1557,80	1557,80	0,24	0	78,22	461	475	AASYVNSAPPPVVMR	M		1680,44	
1807	787,90	1573,79	1573,79	0,39	0	45,47	461	475	AASYVNSAPPPVVMR	M	Oxidation (M) [14]	1638,36	
3597	968,16	2901,47	2901,47	0,77	1	63,51	67	93	AGMTSLPPSPATSEHVPLLDNRPTLER	M	Oxidation (M) [3]	1848,96	
3574	962,83	2885,48	2885,47	3,22	1	38,78	67	93	AGMTSLPPSPATSEHVPLLDNRPTLER	M		1973,70	
1413	713,91	1425,80	1425,80	0,39	0	101,94	196	208	AIMPLLIVAENAR	N	Oxidation (M) [3]	3024,48	
1356	705,91	1409,80	1409,81	-2,97	0	87,07	196	208	AIMPLLIVAENAR	N		2688,68	
1355	705,91	1409,80	1409,81	-3,71	0	88,61	196	208	AIMPLLIVAENAR	N		2605,97	
1358	705,91	1409,81	1409,81	-0,36	0	64,89	196	208	AIMPLLIVAENAR	N		3446,16	
1359	705,91	1409,81	1409,81	-0,09	0	73,26	196	208	AIMPLLIVAENAR	N		2898,18	
1360	705,91	1409,81	1409,81	0,11	0	62,97	196	208	AIMPLLIVAENAR	N		3811,35	
1362	705,91	1409,81	1409,81	0,30	0	61,98	196	208	AIMPLLIVAENAR	N		3994,13	
1363	705,91	1409,81	1409,81	0,32	0	81,96	196	208	AIMPLLIVAENAR	N		3081,08	
1364	705,91	1409,81	1409,81	0,35	0	67,00	196	208	AIMPLLIVAENAR	N		3628,77	
1367	705,91	1409,81	1409,81	0,51	0	73,22	196	208	AIMPLLIVAENAR	N		3263,92	
1368	705,91	1409,81	1409,81	0,72	0	72,21	196	208	AIMPLLIVAENAR	N		2510,32	
1370	705,91	1409,81	1409,81	0,99	0	31,50	196	208	AIMPLLIVAENAR	N		4261,04	
1371	705,91	1409,81	1409,81	1,36	0	40,71	196	208	AIMPLLIVAENAR	N		4176,38	
1401	713,90	1425,79	1425,80	-5,14	0	60,64	196	208	AIMPLLIVAENAR	N	Oxidation (M) [3]	3325,37	
1404	713,91	1425,80	1425,80	-3,38	0	83,60	196	208	AIMPLLIVAENAR	N	Oxidation (M) [3]	2367,91	
1405	713,91	1425,80	1425,80	-0,65	0	74,07	196	208	AIMPLLIVAENAR	N	Oxidation (M) [3]	2657,61	
1406	713,91	1425,80	1425,80	-0,42	0	41,21	196	208	AIMPLLIVAENAR	N	Oxidation (M) [3]	3952,07	
1407	713,91	1425,80	1425,80	-0,18	0	54,50	196	208	AIMPLLIVAENAR	N	Oxidation (M) [3]	3391,54	
1409	713,91	1425,80	1425,80	0,31	0	61,89	196	208	AIMPLLIVAENAR	N	Oxidation (M) [3]	3757,19	
1410	713,91	1425,80	1425,80	0,31	0	81,09	196	208	AIMPLLIVAENAR	N	Oxidation (M) [3]	2842,38	
1411	713,91	1425,80	1425,80	0,33	0	56,03	196	208	AIMPLLIVAENAR	N	Oxidation (M) [3]	3960,11	
1412	713,91	1425,80	1425,80	0,39	0	67,10	196	208	AIMPLLIVAENAR	N	Oxidation (M) [3]	3206,90	
1357	705,91	1409,80	1409,81	-1,07	0	91,51	196	208	AIMPLLIVAENAR	N		2713,99	
1414	713,91	1425,80	1425,80	0,52	0	54,05	196	208	AIMPLLIVAENAR	N	Oxidation (M) [3]	3574,76	
1415	713,91	1425,80	1425,80	0,62	0	81,57	196	208	AIMPLLIVAENAR	N	Oxidation (M) [3]	2470,90	
1416	476,28	1425,80	1425,80	1,35	0	42,36	196	208	AIMPLLIVAENAR	N	Oxidation (M) [3]	2294,80	
1417	713,91	1425,80	1425,80	1,40	0	53,57	196	208	AIMPLLIVAENAR	N	Oxidation (M) [3]	4141,51	
1418	713,91	1425,81	1425,80	2,71	0	79,41	196	208	AIMPLLIVAENAR	N	Oxidation (M) [3]	2289,15	
1471	721,91	1441,80	1441,80	0,55	0	73,00	196	208	AIMPLLIVAENAR	N	Dioxidation (M) [3]	2431,49	
731	575,78	1149,54	1149,54	-0,43	0	56,16	38	49	APDDAPGSPPAR	R		1049,83	
727	575,77	1149,53	1149,54	-5,63	0	39,99	38	49	APDDAPGSPPAR	R		1213,57	
729	575,78	1149,54	1149,54	-4,43	0	41,57	38	49	APDDAPGSPPAR	R		1200,95	
728	575,78	1149,54	1149,54	-4,45	0	48,82	38	49	APDDAPGSPPAR	R		884,49	
3137	756,40	2266,18	2266,18	1,88	0	92,49	359	380	AQQTEELGATLVLDGAPLVDAR	M		2580,46	
3111	756,40	2266,18	2266,18	-0,67	0	35,60	359	380	AQQTEELGATLVLDGAPLVDAR	M		3291,86	
3113	756,40	2266,18	2266,18	-0,61	0	38,64	359	380	AQQTEELGATLVLDGAPLVDAR	M		3867,71	
3114	1134,10	2266,18	2266,18	-0,54	0	37,03	359	380	AQQTEELGATLVLDGAPLVDAR	M		3204,14	
3119	756,40	2266,18	2266,18	-0,30	0	36,96	359	380	AQQTEELGATLVLDGAPLVDAR	M		3658,18	
3121	1134,10	2266,18	2266,18	0,25	0	47,84	359	380	AQQTEELGATLVLDGAPLVDAR	M		3670,90	
3122	1134,10	2266,18	2266,18	0,25	0	39,85	359	380	AQQTEELGATLVLDGAPLVDAR	M		3717,88	
3123	1134,10	2266,18	2266,18	0,34	0	57,38	359	380	AQQTEELGATLVLDGAPLVDAR	M		3136,02	
3112	1134,10	2266,18	2266,18	-0,63	0	62,28	359	380	AQQTEELGATLVLDGAPLVDAR	M		3320,30	
3128	756,40	2266,18	2266,18	0,91	0	41,28	359	380	AQQTEELGATLVLDGAPLVDAR	M		3411,47	
3129	1134,10	2266,18	2266,18	1,13	0	59,58	359	380	AQQTEELGATLVLDGAPLVDAR	M		2950,82	
3130	1134,10	2266,18	2266,18	1,13	0	75,29	359	380	AQQTEELGATLVLDGAPLVDAR	M		2764,71	
3131	1134,10	2266,18	2266,18	1,22	0	42,57	359	380	AQQTEELGATLVLDGAPLVDAR	M		3520,95	
3133	1134,10	2266,18	2266,18	1,31	0	44,01	359	380	AQQTEELGATLVLDGAPLVDAR	M		4018,68	
3134	1134,10	2266,18	2266,18	1,49	0	33,58	359	380	AQQTEELGATLVLDGAPLVDAR	M		3482,46	
3136	1134,10	2266,18	2266,18	1,84	0	65,80	359	380	AQQTEELGATLVLDGAPLVDAR	M		2396,76	
3127	756,40	2266,18	2266,18	0,83	0	81,59	359	380	AQQTEELGATLVLDGAPLVDAR	M		2395,71	
3138	1134,10	2266,19	2266,18	2,37	0	74,33	359	380	AQQTEELGATLVLDGAPLVDAR	M		2580,80	
3140	1134,10	2266,19	2266,18	2,99	0	42,05	359	380	AQQTEELGATLVLDGAPLVDAR	M		3987,29	
444	535,76	1069,51	1069,52	-3,49	0	64,24	253	262	AVIDDGSHTR	A		763,97	
442	535,76	1069,51	1069,52	-5,85	0	58,25	253	262	AVIDDGSHTR	A		1020,64	
445	535,76	1069,51	1069,52	-2,78	0	46,39	253	262	AVIDDGSHTR	A		945,85	
446	535,76	1069,51	1069,52	-2,07	0	40,74	253	262	AVIDDGSHTR	A		1275,24	
448	535,77	1069,52	1069,52	1,93	0	31,55	253	262	AVIDDGSHTR	A		1181,59	
1424	714,89	1427,76	1427,76	-2,87	0	72,99	269	282	DASGTSVIVVDPLR	K		2141,55	
1426	714,89	1427,77	1427,76	2,76	0	60,04	269	282	DASGTSVIVVDPLR	K		2119,15	
1754	778,94	1555,86	1555,86	2,04	1	58,92	269	283	DASGTSVIVVDPLRK	E		1808,88	
1749	778,94	1555,86	1555,86	0,93	1	30,55	269	283	DASGTSVIVVDPLRK	E		2176,55	
1751	778,94	1555,86	1555,86	1,01	1	36,63	269	283	DASGTSVIVVDPLRK	E		2185,14	
1755	778,94	1555,86	1555,86	3,80	1	35,89	269	283	DASGTSVIVVDPLRK	E		1991,50	
1291	696,83	1391,65	1391,65	1,23	0	67,29	336	347	DDAFAAFHETLR	N		2006,75	
1290	464,89	1391,65	1391,65	0,73	0	47,86	336	347	DDAFAAFHETLR	N		2012,56	
1012	641,32	1280,62	1280,62	-2,94	0	64,90	443	453	EITFSNSVEQK	R		1514,53	
1016	641,32	1280,63	1280,62	0,20	0	39,64	443	453	EITFSNSVEQK	R		2468,22	
1456	719,37	1436,72	1436,73	-1,50	1	80,30	443	454	EITFSNSVEQKR	I		1312,85	
1455	719,37	1436,72	1436,73	-1,55	1	65,74	443	454	EITFSNSVEQKR	I		1345,35	
1457	479,92	1436,72	1436,73	-0,80	1	31,60	443	454	EITFSNSVEQKR	I		1314,59	
3393	887,72	2660,14	2660,13	2,22	1	140,28	284	306	EKDESAYVDYADNVNMEFGEHAK	C		1973,98	
3363	666,04	2660,13	2660,13	-2,22	1	42,72	284	306	EKDESAYVDYADNVNMEFGEHAK	C		4709,19	
3367	887,72	2660,13	2660,13	-0,58	1	44,29	284	306	EKDESAYVDYADNVNMEFGEHAK	C		3581,29	
3368	666,04	2660,13	2660,13	-0,52	1	46,46	284	306	EKDESAYVDYADNVNMEFGEHAK	C		3114,31	
3370	666,04	2660,13	2660,13	-0,35	1	52,87	284	306	EKDESAYVDYADNVNMEFGEHAK	C		3297,78	
3371	887,72	2660,13	2660,13	-0,22	1	35,71	284	306	EKDESAYVDYADNVNMEFGEHAK	C		3884,36	
3372	666,04	2660,13	2660,13	-0,19	1	35,62	284	306	EKDESAYVDYADNVNMEFGEHAK	C		2931,84	
3373	666,04	2660,13	2660,13	-0,10	1	41,97	284	306	EKDESAYVDYADNVNMEFGEHAK	C		3482,09	
3374	887,72	2660,13	2660,13	0,06	1	48,63	284	306	EKDESAYVDYADNVNMEFGEHAK	C		3331,49	
3375	887,72	2660,13	2660,13	0,23	1	76,87	284	306	EKDESAYVDYADNVNMEFGEHAK	C		4068,39	
3378	887,72	2660,13	2660,13	0,30	1	70,97	284	306	EKDESAYVDYADNVNMEFGEHAK	C		2965,84	
3381	887,72	2660,14	2660,13	0,61	1	71,16	284	306	EKDESAYVDYADNVNMEFGEHAK	C		3148,48	
3383	887,72	2660,14	2660,13	0,70	1	39,09	284	306	EKDESAYVDYADNVNMEFGEHAK	C		3513,26	
3384	887,72	2660,14	2660,13	0,76	1	75,95	284	306	EKDESAYVDYADNVNMEFGEHAK	C		2598,11	
3385	887,72	2660,14	2660,13	0,79	1	70,22	284	306	EKDESAYVDYADNVNMEFGEHAK	C		3699,17	
3386	887,72	2660,14	2660,13	0,79	1	60,70	284	306	EKDESAYVDYADNVNMEFGEHAK	C		2780,79	
3387	666,04	2660,14	2660,13	0,95	1	32,09	284	306	EKDESAYVDYADNVNMEFGEHAK	C		1805,09	
3389	887,72	2660,14	2660,13	1,52	1	54,34	284	306	EKDESAYVDYADNVNMEFGEHAK	C		2353,02	
3390	887,72	2660,14	2660,13	1,61	1	49,85	284	306	EKDESAYVDYADNVNMEFGEHAK	C		2406,09	
3391	666,04	2660,14	2660,13	1,92	1	51,44	284	306	EKDESAYVDYADNVNMEFGEHAK	C		1981,85	
3366	887,72	2660,13	2660,13	-0,67	1	82,58	284	306	EKDESAYVDYADNVNMEFGEHAK	C		2154,26	
3394	887,72	2660,14	2660,13	3,04	1	61,61	284	306	EKDESAYVDYADNVNMEFGEHAK	C		2415,69	
3425	670,04	2676,13	2676,13	-0,41	1	45,28	284	306	EKDESAYVDYADNVNMEFGEHAK	C	Oxidation (M) [16]	3061,45	
3443	893,05	2676,13	2676,13	1,29	1	121,77	284	306	EKDESAYVDYADNVNMEFGEHAK	C	Oxidation (M) [16]	1900,55	
3519	901,72	2702,15	2702,14	2,06	1	79,33	284	306	EKDESAYVDYADNVNMEFGEHAK	C	Acetyl (K) [2]	2200,44	
3528	907,05	2718,13	2718,14	-2,36	1	87,08	284	306	EKDESAYVDYADNVNMEFGEHAK	C	Acetyl (K) [2], Oxidation (M) [16]	2140,09	
225	500,25	998,49	998,49	-4,19	0	60,95	384	393	HGQAASSVSR	Y		301,69	
228	500,25	998,49	998,49	-3,47	0	57,11	384	393	HGQAASSVSR	Y		483,46	
230	500,25	998,49	998,49	-1,80	0	49,02	384	393	HGQAASSVSR	Y		666,27	
542	550,80	1099,58	1099,58	-0,47	0	65,66	238	247	HIAEFVASAR	P		1349,38	
535	550,80	1099,58	1099,58	-1,07	0	31,95	238	247	HIAEFVASAR	P		2364,82	
531	550,79	1099,57	1099,58	-5,40	0	58,91	238	247	HIAEFVASAR	P		1246,68	
545	550,80	1099,58	1099,58	-0,32	0	33,94	238	247	HIAEFVASAR	P		4032,29	
546	550,80	1099,58	1099,58	-0,23	0	30,49	238	247	HIAEFVASAR	P		2751,35	
547	550,80	1099,58	1099,58	-0,10	0	32,19	238	247	HIAEFVASAR	P		1900,02	
548	550,80	1099,58	1099,58	0,80	0	32,67	238	247	HIAEFVASAR	P		4215,78	
549	550,80	1099,58	1099,58	1,13	0	49,49	238	247	HIAEFVASAR	P		2112,99	
551	550,80	1099,58	1099,58	2,61	0	31,19	238	247	HIAEFVASAR	P		2557,47	
1340	705,88	1409,75	1409,75	-2,96	1	68,15	238	250	HIAEFVASARPGR	Y		1226,39	
1338	470,92	1409,75	1409,75	-3,41	1	33,20	238	250	HIAEFVASARPGR	Y		1215,64	
2039	842,98	1683,95	1683,95	0,78	2	81,95	268	283	KDASGTSVIVVDPLRK	E		1526,58	
2036	842,98	1683,95	1683,95	-2,85	2	58,52	268	283	KDASGTSVIVVDPLRK	E		1556,85	
2038	562,32	1683,95	1683,95	0,02	2	49,94	268	283	KDASGTSVIVVDPLRK	E		1518,28	
2037	562,32	1683,95	1683,95	-0,05	2	61,93	268	283	KDASGTSVIVVDPLRK	E		1565,39	
2128	576,33	1725,96	1725,96	-2,36	2	54,30	268	283	KDASGTSVIVVDPLRK	E	Acetyl (K) [1]	1653,82	
3547	935,75	2804,23	2804,22	0,89	2	79,47	283	306	KEKDESAYVDYADNVNMEFGEHAK	C	Oxidation (M) [17]	1790,35	
3541	698,06	2788,23	2788,23	-0,26	2	45,21	283	306	KEKDESAYVDYADNVNMEFGEHAK	C		1845,48	
3546	702,06	2804,22	2804,22	-0,35	2	49,81	283	306	KEKDESAYVDYADNVNMEFGEHAK	C	Oxidation (M) [17]	1775,53	
2679	966,49	1930,96	1930,96	2,42	0	136,02	173	189	LQALSAQNMDPELAQFR	V		2269,54	
2649	966,48	1930,95	1930,96	-2,35	0	49,31	173	189	LQALSAQNMDPELAQFR	V		2442,09	
2653	644,66	1930,95	1930,96	-1,52	0	30,01	173	189	LQALSAQNMDPELAQFR	V		2734,57	
2659	966,48	1930,96	1930,96	-0,88	0	51,98	173	189	LQALSAQNMDPELAQFR	V		2894,53	
2651	966,48	1930,95	1930,96	-1,75	0	72,02	173	189	LQALSAQNMDPELAQFR	V		2395,39	
2670	644,66	1930,96	1930,96	0,64	0	88,56	173	189	LQALSAQNMDPELAQFR	V		2273,32	
2672	966,49	1930,96	1930,96	0,99	0	52,89	173	189	LQALSAQNMDPELAQFR	V		2518,51	
2674	966,49	1930,96	1930,96	1,54	0	38,26	173	189	LQALSAQNMDPELAQFR	V		2729,86	
2675	966,49	1930,96	1930,96	1,60	0	40,16	173	189	LQALSAQNMDPELAQFR	V		3460,37	
2676	966,49	1930,96	1930,96	1,74	0	46,83	173	189	LQALSAQNMDPELAQFR	V		2812,67	
2671	966,49	1930,96	1930,96	0,98	0	91,08	173	189	LQALSAQNMDPELAQFR	V		2481,63	
2680	966,49	1930,96	1930,96	2,62	0	41,90	173	189	LQALSAQNMDPELAQFR	V		3085,59	
2681	966,49	1930,96	1930,96	2,78	0	40,75	173	189	LQALSAQNMDPELAQFR	V		2711,37	
2737	649,99	1946,94	1946,95	-6,19	0	69,64	173	189	LQALSAQNMDPELAQFR	V	Oxidation (M) [9]	2064,95	
2738	974,48	1946,95	1946,95	-2,81	0	58,12	173	189	LQALSAQNMDPELAQFR	V	Oxidation (M) [9]	2146,69	
2741	974,48	1946,95	1946,95	-1,51	0	43,74	173	189	LQALSAQNMDPELAQFR	V	Oxidation (M) [9]	2464,09	
2751	649,99	1946,95	1946,95	-0,40	0	43,35	173	189	LQALSAQNMDPELAQFR	V	Oxidation (M) [9]	2743,40	
2752	974,48	1946,95	1946,95	-0,40	0	54,39	173	189	LQALSAQNMDPELAQFR	V	Oxidation (M) [9]	3354,09	
2753	649,99	1946,95	1946,95	-0,22	0	41,35	173	189	LQALSAQNMDPELAQFR	V	Oxidation (M) [9]	3303,88	
2755	649,99	1946,95	1946,95	-0,03	0	44,87	173	189	LQALSAQNMDPELAQFR	V	Oxidation (M) [9]	2936,77	
2757	974,48	1946,95	1946,95	0,08	0	33,96	173	189	LQALSAQNMDPELAQFR	V	Oxidation (M) [9]	3702,40	
2759	974,48	1946,95	1946,95	0,21	0	51,92	173	189	LQALSAQNMDPELAQFR	V	Oxidation (M) [9]	3135,70	
2760	649,99	1946,95	1946,95	0,26	0	70,06	173	189	LQALSAQNMDPELAQFR	V	Oxidation (M) [9]	2080,71	
2762	649,99	1946,95	1946,95	0,34	0	46,59	173	189	LQALSAQNMDPELAQFR	V	Oxidation (M) [9]	2056,70	
2764	974,48	1946,95	1946,95	0,47	0	43,93	173	189	LQALSAQNMDPELAQFR	V	Oxidation (M) [9]	2814,44	
2769	974,48	1946,95	1946,95	0,90	0	45,19	173	189	LQALSAQNMDPELAQFR	V	Oxidation (M) [9]	2921,83	
2772	974,48	1946,95	1946,95	1,25	0	58,08	173	189	LQALSAQNMDPELAQFR	V	Oxidation (M) [9]	3930,63	
2773	974,48	1946,95	1946,95	1,30	0	47,55	173	189	LQALSAQNMDPELAQFR	V	Oxidation (M) [9]	3519,32	
2775	974,48	1946,95	1946,95	1,35	0	47,44	173	189	LQALSAQNMDPELAQFR	V	Oxidation (M) [9]	2723,81	
2776	974,48	1946,95	1946,95	1,50	0	31,06	173	189	LQALSAQNMDPELAQFR	V	Oxidation (M) [9]	3327,03	
2777	974,49	1946,96	1946,95	2,28	0	62,58	173	189	LQALSAQNMDPELAQFR	V	Oxidation (M) [9]	2181,73	
2778	974,49	1946,96	1946,95	2,92	0	116,67	173	189	LQALSAQNMDPELAQFR	V	Oxidation (M) [9]	1997,72	
2779	649,99	1946,96	1946,95	3,02	0	91,15	173	189	LQALSAQNMDPELAQFR	V	Oxidation (M) [9]	1995,68	
2829	982,48	1962,95	1962,95	1,54	0	72,62	173	189	LQALSAQNMDPELAQFR	V	Dioxidation (M) [9]	2092,50	
1729	774,45	1546,88	1546,88	0,54	1	105,44	158	172	LRTQVTGFLSGALGK	L		2015,54	
496	547,77	1093,53	1093,53	-2,71	0	49,00	94	103	MGVDHPLPGR	T	Oxidation (M) [1]	1076,23	
462	539,78	1077,54	1077,54	-1,68	0	33,97	94	103	MGVDHPLPGR	T		1186,34	
461	539,77	1077,53	1077,54	-5,95	0	44,43	94	103	MGVDHPLPGR	T		1228,61	
498	547,77	1093,53	1093,53	-0,52	0	38,52	94	103	MGVDHPLPGR	T	Oxidation (M) [1]	1312,59	
2541	952,44	1902,87	1902,87	0,03	1	122,03	332	347	MHDKDDAFAAFHETLR	N		1649,01	
2536	635,30	1902,87	1902,87	-0,28	1	85,37	332	347	MHDKDDAFAAFHETLR	N		1644,92	
2542	476,72	1902,87	1902,87	0,24	1	33,50	332	347	MHDKDDAFAAFHETLR	N		3532,40	
2543	476,72	1902,87	1902,87	0,37	1	58,40	332	347	MHDKDDAFAAFHETLR	N		1643,83	
2555	476,72	1902,87	1902,87	1,32	1	51,71	332	347	MHDKDDAFAAFHETLR	N		2216,02	
2604	480,72	1918,86	1918,86	-3,80	1	47,95	332	347	MHDKDDAFAAFHETLR	N	Oxidation (M) [1]	1656,68	
2606	640,63	1918,86	1918,86	-1,80	1	63,75	332	347	MHDKDDAFAAFHETLR	N	Oxidation (M) [1]	1602,24	
2611	960,44	1918,86	1918,86	-0,43	1	111,93	332	347	MHDKDDAFAAFHETLR	N	Oxidation (M) [1]	1602,71	
2619	480,72	1918,86	1918,86	0,14	1	30,90	332	347	MHDKDDAFAAFHETLR	N	Oxidation (M) [1]	3228,81	
2620	480,72	1918,86	1918,86	0,18	1	34,64	332	347	MHDKDDAFAAFHETLR	N	Oxidation (M) [1]	2490,07	
2621	480,72	1918,86	1918,86	0,49	1	41,10	332	347	MHDKDDAFAAFHETLR	N	Oxidation (M) [1]	1594,72	
2622	480,72	1918,86	1918,86	0,53	1	31,59	332	347	MHDKDDAFAAFHETLR	N	Oxidation (M) [1]	2679,77	
2625	480,72	1918,87	1918,86	1,87	1	34,07	332	347	MHDKDDAFAAFHETLR	N	Oxidation (M) [1]	1779,93	
2694	484,72	1934,85	1934,86	-2,15	1	54,95	332	347	MHDKDDAFAAFHETLR	N	Dioxidation (M) [1]	1635,03	
2695	645,96	1934,85	1934,86	-1,91	1	48,99	332	347	MHDKDDAFAAFHETLR	N	Dioxidation (M) [1]	1632,02	
2722	649,30	1944,87	1944,88	-2,07	1	54,59	332	347	MHDKDDAFAAFHETLR	N	Acetyl (K) [4]	1794,65	
2723	487,23	1944,88	1944,88	-0,73	1	49,13	332	347	MHDKDDAFAAFHETLR	N	Acetyl (K) [4]	1795,45	
2725	649,30	1944,88	1944,88	-0,32	1	41,47	332	347	MHDKDDAFAAFHETLR	N	Acetyl (K) [4]	1773,01	
2728	973,45	1944,88	1944,88	1,72	1	66,21	332	347	MHDKDDAFAAFHETLR	N	Acetyl (K) [4]	1855,52	
2820	491,23	1960,87	1960,87	-0,21	1	32,41	332	347	MHDKDDAFAAFHETLR	N	Oxidation (M) [1], Acetyl (K) [4]	1684,74	
2821	981,44	1960,88	1960,87	0,77	1	45,95	332	347	MHDKDDAFAAFHETLR	N	Oxidation (M) [1], Acetyl (K) [4]	1779,50	
1474	483,23	1446,66	1446,67	-4,04	1	31,80	381	393	MMKHGQAASSVSR	Y	Oxidation (M) [1], Acetyl (K) [3]	777,12	
767	581,77	1161,52	1161,53	-3,13	0	40,65	348	358	NGGDPSHHVSR	A		305,47	
3153	1140,05	2278,08	2278,07	2,84	0	116,17	209	228	NPGLNLVPLHMDMAEDEEVR	T		2290,77	
3149	760,36	2278,07	2278,07	-0,10	0	52,30	209	228	NPGLNLVPLHMDMAEDEEVR	T		2523,78	
3151	760,37	2278,07	2278,07	0,44	0	42,23	209	228	NPGLNLVPLHMDMAEDEEVR	T		2513,93	
3148	760,36	2278,07	2278,07	-1,99	0	89,57	209	228	NPGLNLVPLHMDMAEDEEVR	T		2326,28	
3152	760,37	2278,08	2278,07	2,31	0	109,92	209	228	NPGLNLVPLHMDMAEDEEVR	T		2285,95	
3172	765,70	2294,07	2294,07	0,84	0	78,74	209	228	NPGLNLVPLHMDMAEDEEVR	T	Oxidation (M) [11]	2052,70	
3173	1148,04	2294,07	2294,07	1,90	0	56,25	209	228	NPGLNLVPLHMDMAEDEEVR	T	Oxidation (M) [11]	2055,73	
3212	771,03	2310,06	2310,06	0,30	0	78,30	209	228	NPGLNLVPLHMDMAEDEEVR	T	Oxidation (M) [11], Oxidation (M) [13]	1898,24	
3223	771,03	2310,06	2310,06	0,84	0	42,50	209	228	NPGLNLVPLHMDMAEDEEVR	T	Oxidation (M) [11], Oxidation (M) [13]	2113,31	
3224	1156,04	2310,07	2310,06	2,18	0	39,78	209	228	NPGLNLVPLHMDMAEDEEVR	T	Oxidation (M) [11], Oxidation (M) [13]	1902,40	
3750	801,88	3203,51	3203,52	-2,25	1	74,80	209	237	NPGLNLVPLHMDMAEDEEVRTQPPMAGSR	H		2231,13	
3751	801,89	3203,52	3203,52	1,03	1	55,44	209	237	NPGLNLVPLHMDMAEDEEVRTQPPMAGSR	H		2192,83	
3752	1068,85	3203,52	3203,52	1,69	1	56,77	209	237	NPGLNLVPLHMDMAEDEEVRTQPPMAGSR	H		2199,95	
3754	805,89	3219,51	3219,51	0,60	1	41,73	209	237	NPGLNLVPLHMDMAEDEEVRTQPPMAGSR	H	Oxidation (M) [11]	2016,26	
3755	1074,18	3219,51	3219,51	1,18	1	42,64	209	237	NPGLNLVPLHMDMAEDEEVRTQPPMAGSR	H	Oxidation (M) [11]	2026,78	
3756	1074,18	3219,52	3219,51	1,64	1	55,62	209	237	NPGLNLVPLHMDMAEDEEVRTQPPMAGSR	H	Oxidation (M) [13]	2078,11	
3759	809,88	3235,51	3235,51	0,75	1	30,54	209	237	NPGLNLVPLHMDMAEDEEVRTQPPMAGSR	H	Dioxidation (M) [11]	1923,08	
3767	1084,84	3251,51	3251,50	2,85	1	47,62	209	237	NPGLNLVPLHMDMAEDEEVRTQPPMAGSR	H	Oxidation (M) [11], Oxidation (M) [13], Oxidation (M) [25]	1797,99	
62	452,71	903,41	903,42	-3,92	1	40,35	428	435	NRADSEGR	V		302,01	
1178	679,34	1356,66	1356,66	-4,80	0	103,47	22	34	PSQTNADTTPLGR	R		1228,06	
1175	679,33	1356,65	1356,66	-6,56	0	64,35	22	34	PSQTNADTTPLGR	R		1174,27	
1177	679,34	1356,66	1356,66	-4,95	0	103,33	22	34	PSQTNADTTPLGR	R		1235,48	
1182	679,34	1356,66	1356,66	-2,55	0	71,14	22	34	PSQTNADTTPLGR	R		1265,36	
1189	679,34	1356,66	1356,66	0,70	0	58,49	22	34	PSQTNADTTPLGR	R		1085,30	
1190	679,34	1356,66	1356,66	0,86	0	80,80	22	34	PSQTNADTTPLGR	R		1340,58	
1193	679,34	1356,66	1356,66	0,95	0	46,01	22	34	PSQTNADTTPLGR	R		1452,01	
1675	764,32	1526,62	1526,63	-4,03	0	69,10	53	65	QDSPEDSAQTMFR	R	Oxidation (M) [11]	1212,16	
1634	756,33	1510,64	1510,64	2,35	0	65,66	53	65	QDSPEDSAQTMFR	R		1700,08	
1677	764,32	1526,63	1526,63	-1,13	0	48,02	53	65	QDSPEDSAQTMFR	R	Oxidation (M) [11]	1446,91	
1678	764,32	1526,63	1526,63	0,81	0	30,34	53	65	QDSPEDSAQTMFR	R	Oxidation (M) [11]	1629,07	
1679	764,32	1526,64	1526,63	3,05	0	64,07	53	65	QDSPEDSAQTMFR	R	Oxidation (M) [11]	1377,90	
2304	604,60	1810,78	1810,79	-4,94	1	66,49	51	65	QRQDSPEDSAQTMFR	R	Oxidation (M) [13]	1246,97	
2267	599,27	1794,79	1794,80	-1,14	1	38,47	51	65	QRQDSPEDSAQTMFR	R		1333,13	
2268	599,27	1794,80	1794,80	0,20	1	51,46	51	65	QRQDSPEDSAQTMFR	R		1515,82	
2306	906,40	1810,79	1810,79	-2,55	1	40,29	51	65	QRQDSPEDSAQTMFR	R	Oxidation (M) [13]	1144,01	
2307	604,60	1810,79	1810,79	-1,60	1	49,09	51	65	QRQDSPEDSAQTMFR	R	Oxidation (M) [13]	1063,48	
3664	761,40	3041,57	3041,57	-0,19	2	89,59	66	93	RAGMTSLPPSPATSEHVPLLDNRPTLER	M		1890,30	
3665	1014,87	3041,58	3041,57	1,69	2	58,98	66	93	RAGMTSLPPSPATSEHVPLLDNRPTLER	M		1853,61	
3666	761,40	3041,58	3041,57	1,69	2	67,35	66	93	RAGMTSLPPSPATSEHVPLLDNRPTLER	M		1812,85	
3673	765,40	3057,57	3057,57	0,02	2	70,21	66	93	RAGMTSLPPSPATSEHVPLLDNRPTLER	M	Oxidation (M) [4]	1777,44	
3676	765,40	3057,57	3057,57	1,76	2	75,43	66	93	RAGMTSLPPSPATSEHVPLLDNRPTLER	M	Oxidation (M) [4]	1719,29	
3678	765,40	3057,58	3057,57	2,93	2	64,02	66	93	RAGMTSLPPSPATSEHVPLLDNRPTLER	M	Oxidation (M) [4]	1710,28	
1072	653,83	1305,64	1305,64	0,97	1	36,87	37	49	RAPDDAPGSPPAR	R		1003,64	
1067	653,82	1305,63	1305,64	-7,57	1	31,32	37	49	RAPDDAPGSPPAR	R		991,84	
178	487,75	973,49	973,49	-3,44	1	50,54	409	416	RNETLGER	T		696,59	
1258	460,92	1379,72	1379,73	-2,17	1	45,75	133	146	SAGPATAARPQPTR	T		960,87	
1007	639,86	1277,70	1277,70	2,66	0	85,25	160	172	TQVTGFLSGALGK	L		2399,07	
991	639,86	1277,70	1277,70	-0,56	0	59,64	160	172	TQVTGFLSGALGK	L		2985,48	
992	639,86	1277,70	1277,70	-0,47	0	70,32	160	172	TQVTGFLSGALGK	L		2801,16	
990	639,85	1277,69	1277,70	-2,35	0	72,84	160	172	TQVTGFLSGALGK	L		2248,82	
994	639,86	1277,70	1277,70	-0,22	0	80,88	160	172	TQVTGFLSGALGK	L		2066,58	
995	639,86	1277,70	1277,70	-0,14	0	42,63	160	172	TQVTGFLSGALGK	L		3168,88	
996	639,86	1277,70	1277,70	0,03	0	42,81	160	172	TQVTGFLSGALGK	L		3352,17	
997	639,86	1277,70	1277,70	0,05	0	55,22	160	172	TQVTGFLSGALGK	L		3534,97	
998	639,86	1277,70	1277,70	0,06	0	40,91	160	172	TQVTGFLSGALGK	L		3901,15	
999	639,86	1277,70	1277,70	0,14	0	44,78	160	172	TQVTGFLSGALGK	L		3718,51	
1001	639,86	1277,70	1277,70	0,34	0	58,81	160	172	TQVTGFLSGALGK	L		2617,32	
1002	639,86	1277,70	1277,70	0,77	0	35,15	160	172	TQVTGFLSGALGK	L		4265,71	
1003	639,86	1277,70	1277,70	0,81	0	34,82	160	172	TQVTGFLSGALGK	L		4083,50	
1004	639,86	1277,70	1277,70	1,10	0	30,75	160	172	TQVTGFLSGALGK	L		4136,82	
1005	639,86	1277,70	1277,70	2,05	0	48,96	160	172	TQVTGFLSGALGK	L		2300,41	
993	639,86	1277,70	1277,70	-0,28	0	82,12	160	172	TQVTGFLSGALGK	L		2433,19	
455	538,28	1074,54	1074,54	-2,74	0	74,88	147	157	TSAGQQATVGR	L		943,32	
456	538,28	1074,54	1074,54	-2,18	0	69,32	147	157	TSAGQQATVGR	L		674,56	
1117	662,33	1322,64	1322,64	-0,11	0	108,06	119	132	TSTASAAQVASSSR	S		1350,73	
1101	662,33	1322,64	1322,64	-4,08	0	91,70	119	132	TSTASAAQVASSSR	S		1256,17	
1103	662,33	1322,64	1322,64	-3,37	0	91,57	119	132	TSTASAAQVASSSR	S		892,04	
1104	662,33	1322,64	1322,64	-3,25	0	55,06	119	132	TSTASAAQVASSSR	S		1666,04	
1105	662,33	1322,64	1322,64	-2,78	0	54,47	119	132	TSTASAAQVASSSR	S		1633,50	
1107	662,33	1322,64	1322,64	-2,41	0	78,50	119	132	TSTASAAQVASSSR	S		1535,34	
1109	662,33	1322,64	1322,64	-1,44	0	90,96	119	132	TSTASAAQVASSSR	S		1074,53	
1110	662,33	1322,64	1322,64	-1,18	0	49,69	119	132	TSTASAAQVASSSR	S		2351,18	
1111	662,33	1322,64	1322,64	-0,62	0	34,90	119	132	TSTASAAQVASSSR	S		3236,12	
1112	662,33	1322,64	1322,64	-0,42	0	56,75	119	132	TSTASAAQVASSSR	S		3790,01	
1114	662,33	1322,64	1322,64	-0,27	0	51,69	119	132	TSTASAAQVASSSR	S		3606,30	
1115	662,33	1322,64	1322,64	-0,15	0	70,83	119	132	TSTASAAQVASSSR	S		1702,91	
1116	662,33	1322,64	1322,64	-0,12	0	78,15	119	132	TSTASAAQVASSSR	S		1323,42	
1100	662,33	1322,64	1322,64	-5,05	0	103,99	119	132	TSTASAAQVASSSR	S		1271,22	
1119	662,33	1322,64	1322,64	0,04	0	49,06	119	132	TSTASAAQVASSSR	S		3052,70	
1120	662,33	1322,64	1322,64	0,04	0	40,93	119	132	TSTASAAQVASSSR	S		2680,47	
1121	662,33	1322,64	1322,64	0,24	0	77,26	119	132	TSTASAAQVASSSR	S		3973,81	
1123	662,33	1322,64	1322,64	0,53	0	57,21	119	132	TSTASAAQVASSSR	S		1970,54	
1124	662,33	1322,64	1322,64	0,77	0	45,56	119	132	TSTASAAQVASSSR	S		4157,89	
1125	662,33	1322,64	1322,64	1,06	0	48,16	119	132	TSTASAAQVASSSR	S		2662,97	
1126	662,33	1322,64	1322,64	1,07	0	68,72	119	132	TSTASAAQVASSSR	S		2863,38	
1127	662,33	1322,65	1322,64	4,76	0	64,19	119	132	TSTASAAQVASSSR	S		1695,61	
2057	854,90	1707,78	1707,79	-1,73	0	108,35	104	118	TWYETGHTTASLADR	T		1559,02	
2052	570,27	1707,78	1707,79	-5,01	0	58,54	104	118	TWYETGHTTASLADR	T		1555,98	
2064	854,90	1707,78	1707,79	-0,61	0	93,00	104	118	TWYETGHTTASLADR	T		1789,91	
2067	854,90	1707,78	1707,79	-0,40	0	51,62	104	118	TWYETGHTTASLADR	T		4135,30	
2076	854,90	1707,79	1707,79	0,39	0	38,62	104	118	TWYETGHTTASLADR	T		2190,49	
2084	854,90	1707,79	1707,79	0,85	0	54,31	104	118	TWYETGHTTASLADR	T		2077,49	
2091	854,90	1707,79	1707,79	1,34	0	55,63	104	118	TWYETGHTTASLADR	T		3772,43	
2096	854,90	1707,79	1707,79	2,78	0	41,24	104	118	TWYETGHTTASLADR	T		2169,70	
3045	703,40	2107,18	2107,18	0,31	1	51,07	190	208	VLDVDRAIMPLLIVAENAR	N		2959,40	
3051	708,73	2123,18	2123,18	0,27	1	41,24	190	208	VLDVDRAIMPLLIVAENAR	N	Oxidation (M) [9]	2745,26	
2203	881,42	1760,83	1760,83	-0,94	0	88,87	3	21	VSSANAGVPASSADNTSAR	P		1045,06	
2199	881,42	1760,82	1760,83	-4,13	0	31,85	3	21	VSSANAGVPASSADNTSAR	P		1252,45	
2198	881,42	1760,82	1760,83	-5,98	0	47,49	3	21	VSSANAGVPASSADNTSAR	P		1103,56	
2202	881,42	1760,82	1760,83	-2,69	0	70,32	3	21	VSSANAGVPASSADNTSAR	P		1244,90	
2201	881,42	1760,82	1760,83	-3,05	0	84,56	3	21	VSSANAGVPASSADNTSAR	P		1028,64	
3698	1034,17	3099,48	3099,48	0,05	1	75,17	3	34	VSSANAGVPASSADNTSARPSQTNADTTPLGR	R		1413,26	
3694	1034,17	3099,48	3099,48	-0,72	1	43,37	3	34	VSSANAGVPASSADNTSARPSQTNADTTPLGR	R		1635,69	
3696	1034,17	3099,48	3099,48	-0,33	1	54,65	3	34	VSSANAGVPASSADNTSARPSQTNADTTPLGR	R		1600,01	
3701	775,88	3099,49	3099,48	1,70	1	53,92	3	34	VSSANAGVPASSADNTSARPSQTNADTTPLGR	R		1405,10	
3703	1034,17	3099,49	3099,48	2,96	1	61,49	3	34	VSSANAGVPASSADNTSARPSQTNADTTPLGR	R		1374,29	
3704	775,88	3099,49	3099,48	3,44	1	48,48	3	34	VSSANAGVPASSADNTSARPSQTNADTTPLGR	R		1388,36	
3705	1550,75	3099,49	3099,48	3,53	1	30,55	3	34	VSSANAGVPASSADNTSARPSQTNADTTPLGR	R		1390,00	
3707	1034,17	3099,49	3099,48	4,22	1	66,36	3	34	VSSANAGVPASSADNTSARPSQTNADTTPLGR	R		1731,01	
2020	841,93	1681,84	1681,84	-1,25	0	51,09	394	408	YLGNHPEQSTVPVNK	R		1125,41	
2016	561,62	1681,84	1681,84	-1,98	0	34,71	394	408	YLGNHPEQSTVPVNK	R		1122,90	
2014	841,93	1681,84	1681,84	-2,78	0	34,82	394	408	YLGNHPEQSTVPVNK	R		1239,76	
2391	919,98	1837,94	1837,94	-0,89	1	75,27	394	409	YLGNHPEQSTVPVNKR	N		1008,39	
2388	613,65	1837,94	1837,94	-1,69	1	47,20	394	409	YLGNHPEQSTVPVNKR	N		1186,88	
2387	613,65	1837,94	1837,94	-2,44	1	63,40	394	409	YLGNHPEQSTVPVNKR	N		1004,06	
2398	613,66	1837,94	1837,94	-0,14	1	46,92	394	409	YLGNHPEQSTVPVNKR	N		1370,96	
2404	613,66	1837,95	1837,94	0,88	1	43,88	394	409	YLGNHPEQSTVPVNKR	N		1407,88	
