# Supplementary material for: Autoacetylation of the Ralstonia solanacearum Effector PopP2 Targets a Lysine Residue Essential for RRS1-R-Mediated Immunity in Arabidopsis
Source: PLoS Pathog. 2010 Nov 18;6(11):e1001202. doi: 10.1371/journal.ppat.1001202 (PMC2987829; doi:10.1371/journal.ppat.1001202)
Supplement: Table S2 — Liquid chromatography/tandem mass spectrometry (LC-MS/MS) analysis of GST-PopP2-C321A. (0.71 MB RTF) [file ppat.1001202.s005.rtf]

Description	accession	(a) Mass	(b) Coverage	(c) #peptides	 (d) emPAI	(e) SC (relevant+duplicated)							
PopP2-C321A	Q8Y125	52819,67	82,79	46	506,23	200							
(f) Query	(g) Observed	(h) Mr (expt)	(i) Mr (calc)	(j) delta	(k) miss	(l) score	(m) start	stop	(n) sequence	 	(o) modifications	(p) R.T.	
1727	779,91	1557,80	1557,80	0,10	0	77,38	461	475	AASYVNSAPPPVVMR	M	 	1718,51	
1724	520,27	1557,79	1557,80	-3,09	0	32,13	461	475	AASYVNSAPPPVVMR	M	 	1784,77	
1722	779,90	1557,79	1557,80	-4,16	0	73,06	461	475	AASYVNSAPPPVVMR	M	 	1753,59	
1746	787,90	1573,79	1573,79	-4,41	0	66,23	461	475	AASYVNSAPPPVVMR	M	Oxidation (M) [14]	1532,24	
1748	787,90	1573,79	1573,79	-3,52	0	44,21	461	475	AASYVNSAPPPVVMR	M	Oxidation (M) [14]	1644,58	
1749	525,60	1573,79	1573,79	-2,27	0	30,27	461	475	AASYVNSAPPPVVMR	M	Oxidation (M) [14]	1540,85	
1750	787,90	1573,79	1573,79	-1,48	0	55,20	461	475	AASYVNSAPPPVVMR	M	Oxidation (M) [14]	1611,70	
1752	787,90	1573,79	1573,79	-1,13	0	42,07	461	475	AASYVNSAPPPVVMR	M	Oxidation (M) [14]	1667,25	
1754	525,60	1573,79	1573,79	-0,64	0	32,44	461	475	AASYVNSAPPPVVMR	M	Oxidation (M) [14]	1490,46	
1755	787,90	1573,79	1573,79	-0,31	0	49,27	461	475	AASYVNSAPPPVVMR	M	Oxidation (M) [14]	1485,76	
1756	787,90	1573,79	1573,79	-0,18	0	32,29	461	475	AASYVNSAPPPVVMR	M	Oxidation (M) [14]	2087,69	
1758	787,90	1573,79	1573,79	0,71	0	34,14	461	475	AASYVNSAPPPVVMR	M	Oxidation (M) [14]	1886,88	
3250	968,16	2901,47	2901,47	0,95	1	45,69	67	93	AGMTSLPPSPATSEHVPLLDNRPTLER	M	Oxidation (M) [3]	1883,54	
1413	705,91	1409,81	1409,81	2,66	0	86,88	196	208	AIMPLLIVAENAR	N	 	2740,22	
1410	705,91	1409,81	1409,81	-0,07	0	76,92	196	208	AIMPLLIVAENAR	N	 	2729,29	
1415	705,92	1409,82	1409,81	8,31	0	32,24	196	208	AIMPLLIVAENAR	N	 	2949,95	
1451	713,91	1425,80	1425,80	-3,73	0	86,87	196	208	AIMPLLIVAENAR	N	Oxidation (M) [3]	2407,06	
1452	713,91	1425,80	1425,80	-1,18	0	62,10	196	208	AIMPLLIVAENAR	N	Oxidation (M) [3]	2685,20	
1454	713,91	1425,80	1425,80	0,32	0	68,16	196	208	AIMPLLIVAENAR	N	Oxidation (M) [3]	2320,34	
1455	713,91	1425,80	1425,80	0,73	0	74,30	196	208	AIMPLLIVAENAR	N	Oxidation (M) [3]	2501,89	
1456	713,91	1425,80	1425,80	1,04	0	33,11	196	208	AIMPLLIVAENAR	N	Oxidation (M) [3]	3290,97	
1457	713,91	1425,80	1425,80	1,60	0	35,75	196	208	AIMPLLIVAENAR	N	Oxidation (M) [3]	3329,82	
1460	713,91	1425,80	1425,80	2,09	0	56,71	196	208	AIMPLLIVAENAR	N	Oxidation (M) [3]	2720,83	
1462	713,91	1425,81	1425,80	2,69	0	32,61	196	208	AIMPLLIVAENAR	N	Oxidation (M) [3]	2731,18	
1506	721,91	1441,80	1441,80	0,47	0	71,34	196	208	AIMPLLIVAENAR	N	Dioxidation (M) [3]	2462,91	
737	575,78	1149,54	1149,54	-4,07	0	63,50	38	49	APDDAPGSPPAR	R	 	915,11	
736	575,78	1149,54	1149,54	-4,76	0	32,77	38	49	APDDAPGSPPAR	R	 	1286,31	
735	575,78	1149,54	1149,54	-5,01	0	38,48	38	49	APDDAPGSPPAR	R	 	1269,66	
738	575,78	1149,54	1149,54	-2,41	0	47,43	38	49	APDDAPGSPPAR	R	 	1099,73	
2883	756,40	2266,18	2266,18	1,69	0	67,52	359	380	AQQTEELGATLVLDGAPLVDAR	M	 	2614,01	
2882	1134,10	2266,18	2266,18	0,87	0	45,04	359	380	AQQTEELGATLVLDGAPLVDAR	M	 	2435,28	
2881	756,40	2266,18	2266,18	0,72	0	54,52	359	380	AQQTEELGATLVLDGAPLVDAR	M	 	2429,11	
2884	1134,10	2266,18	2266,18	1,75	0	60,05	359	380	AQQTEELGATLVLDGAPLVDAR	M	 	2618,72	
2885	756,40	2266,19	2266,18	2,20	0	32,26	359	380	AQQTEELGATLVLDGAPLVDAR	M	 	2528,03	
442	535,76	1069,51	1069,52	-3,68	0	67,08	253	262	AVIDDGSHTR	A	 	793,34	
443	535,76	1069,51	1069,52	-2,89	0	61,88	253	262	AVIDDGSHTR	A	 	978,29	
445	535,76	1069,51	1069,52	-1,31	0	41,33	253	262	AVIDDGSHTR	A	 	1181,50	
1469	714,89	1427,76	1427,76	0,87	0	51,63	269	282	DASGTSVIVVDPLR	K	 	2146,54	
1720	778,94	1555,86	1555,86	1,87	1	49,55	269	283	DASGTSVIVVDPLRK	E	 	1841,24	
1347	696,83	1391,65	1391,65	-0,19	0	64,65	336	347	DDAFAAFHETLR	N	 	2039,47	
1348	464,89	1391,65	1391,65	0,54	0	45,05	336	347	DDAFAAFHETLR	N	 	2037,60	
3003	807,34	2418,99	2418,99	1,02	0	64,79	286	306	DESAYVDYADNVNMEFGEHAK	C	Oxidation (M) [14]	2154,05	
3002	807,34	2418,99	2418,99	-2,17	0	36,42	286	306	DESAYVDYADNVNMEFGEHAK	C	Oxidation (M) [14]	2173,22	
1079	641,32	1280,62	1280,62	-3,17	0	59,04	443	453	EITFSNSVEQK	R	 	1550,38	
1080	641,32	1280,62	1280,62	-1,28	0	49,60	443	453	EITFSNSVEQK	R	 	1481,36	
1081	641,32	1280,62	1280,62	0,09	0	31,51	443	453	EITFSNSVEQK	R	 	2494,64	
1099	641,32	1280,63	1280,62	4,59	0	32,65	443	453	EITFSNSVEQK	R		2012,90	
1496	719,37	1436,72	1436,73	-1,44	1	70,99	443	454	EITFSNSVEQKR	I	 	1346,94	
1497	479,92	1436,72	1436,73	-1,42	1	31,09	443	454	EITFSNSVEQKR	I	 	1346,26	
3091	887,72	2660,14	2660,13	0,85	1	137,74	284	306	EKDESAYVDYADNVNMEFGEHAK	C	 	2004,31	
3090	666,04	2660,13	2660,13	0,10	1	51,40	284	306	EKDESAYVDYADNVNMEFGEHAK	C	 	2001,85	
3105	670,04	2676,13	2676,13	0,83	1	39,96	284	306	EKDESAYVDYADNVNMEFGEHAK	C	Oxidation (M) [16]	1931,57	
3107	893,05	2676,13	2676,13	1,02	1	84,66	284	306	EKDESAYVDYADNVNMEFGEHAK	C	Oxidation (M) [16]	1681,12	
3108	893,05	2676,13	2676,13	1,26	1	111,95	284	306	EKDESAYVDYADNVNMEFGEHAK	C	Oxidation (M) [16]	1933,13	
3110	893,05	2676,13	2676,13	1,80	1	38,50	284	306	EKDESAYVDYADNVNMEFGEHAK	C	Oxidation (M) [16]	2145,00	
3169	680,54	2718,14	2718,14	-0,90	1	61,14	284	306	EKDESAYVDYADNVNMEFGEHAK	C	Acetyl (K) [2], Oxidation (M) [16]	2060,83	
3170	907,05	2718,14	2718,14	0,23	1	89,24	284	306	EKDESAYVDYADNVNMEFGEHAK	C	Acetyl (K) [2], Oxidation (M) [16]	2059,07	
228	500,25	998,49	998,49	-3,93	0	54,19	384	393	HGQAASSVSR	Y	 	484,74	
230	500,25	998,49	998,49	-3,53	0	44,57	384	393	HGQAASSVSR	Y	 	666,64	
541	550,79	1099,58	1099,58	-2,12	0	69,56	238	247	HIAEFVASAR	P	 	1344,95	
537	550,79	1099,57	1099,58	-5,76	0	42,03	238	247	HIAEFVASAR	P	 	1253,52	
536	550,79	1099,57	1099,58	-5,94	0	55,75	238	247	HIAEFVASAR	P	 	1282,61	
542	550,80	1099,58	1099,58	-1,60	0	65,61	238	247	HIAEFVASAR	P	 	1376,44	
543	550,80	1099,58	1099,58	-1,41	0	32,76	238	247	HIAEFVASAR	P	 	1729,80	
544	550,80	1099,58	1099,58	-0,80	0	43,45	238	247	HIAEFVASAR	P	 	1742,76	
548	550,80	1099,58	1099,58	-0,20	0	69,39	238	247	HIAEFVASAR	P	 	1560,30	
549	550,80	1099,58	1099,58	0,35	0	43,09	238	247	HIAEFVASAR	P	 	2113,36	
550	550,80	1099,58	1099,58	0,40	0	35,53	238	247	HIAEFVASAR	P	 	1929,52	
555	550,80	1099,58	1099,58	1,50	0	37,17	238	247	HIAEFVASAR	P	 	3535,82	
557	550,80	1099,58	1099,58	2,26	0	45,58	238	247	HIAEFVASAR	P	 	2085,58	
1396	705,88	1409,75	1409,75	-3,33	1	56,38	238	250	HIAEFVASARPGR	Y	 	1263,54	
1395	470,92	1409,75	1409,75	-4,20	1	35,34	238	250	HIAEFVASARPGR	Y	 	1251,07	
138	479,32	956,63	956,63	-0,27	0	46,26	323	331	ILSLSLALK	M	 	2249,76	
1713	519,63	1555,85	1555,86	-1,29	1	37,86	268	282	KDASGTSVIVVDPLR	K	 	1812,49	
1960	842,98	1683,95	1683,95	-0,12	2	78,51	268	283	KDASGTSVIVVDPLRK	E	 	1562,76	
1958	842,98	1683,95	1683,95	-3,73	2	53,12	268	283	KDASGTSVIVVDPLRK	E	 	1591,68	
1957	562,32	1683,94	1683,95	-4,93	2	54,20	268	283	KDASGTSVIVVDPLRK	E	 	1644,39	
1961	562,32	1683,95	1683,95	-0,10	2	49,35	268	283	KDASGTSVIVVDPLRK	E	 	1553,12	
2041	576,33	1725,96	1725,96	-2,76	2	53,95	268	283	KDASGTSVIVVDPLRK	E	Acetyl (K) [1]	1689,82	
3208	698,06	2788,23	2788,23	0,57	2	50,80	283	306	KEKDESAYVDYADNVNMEFGEHAK	C	 	1877,83	
3215	702,06	2804,22	2804,22	-0,55	2	41,10	283	306	KEKDESAYVDYADNVNMEFGEHAK	C	Oxidation (M) [17]	1808,50	
2579	974,48	1946,96	1946,95	1,69	0	108,02	173	189	LQALSAQNMDPELAQFR	V	Oxidation (M) [9]	2028,66	
2527	966,49	1930,96	1930,96	-0,12	0	90,28	173	189	LQALSAQNMDPELAQFR	V	 	2356,79	
2529	966,49	1930,96	1930,96	1,27	0	84,55	173	189	LQALSAQNMDPELAQFR	V	 	2302,13	
2566	649,99	1946,95	1946,95	-2,00	0	81,87	173	189	LQALSAQNMDPELAQFR	V	Oxidation (M) [9]	2088,99	
2528	644,66	1930,96	1930,96	0,42	0	90,86	173	189	LQALSAQNMDPELAQFR	V	 	2305,74	
2568	649,99	1946,95	1946,95	-1,23	0	63,45	173	189	LQALSAQNMDPELAQFR	V	Oxidation (M) [9]	2110,26	
2570	649,99	1946,95	1946,95	0,11	0	52,10	173	189	LQALSAQNMDPELAQFR	V	Oxidation (M) [9]	2221,30	
2574	974,48	1946,95	1946,95	0,64	0	91,71	173	189	LQALSAQNMDPELAQFR	V	Oxidation (M) [9]	2214,89	
2575	649,99	1946,95	1946,95	0,69	0	35,28	173	189	LQALSAQNMDPELAQFR	V	Oxidation (M) [9]	2639,83	
2577	649,99	1946,95	1946,95	1,50	0	89,40	173	189	LQALSAQNMDPELAQFR	V	Oxidation (M) [9]	2027,06	
2567	974,48	1946,95	1946,95	-1,61	0	95,48	173	189	LQALSAQNMDPELAQFR	V	Oxidation (M) [9]	2177,71	
2618	655,32	1962,95	1962,95	0,35	0	32,60	173	189	LQALSAQNMDPELAQFR	V	Dioxidation (M) [9]	2129,37	
2619	982,48	1962,95	1962,95	1,80	0	56,82	173	189	LQALSAQNMDPELAQFR	V	Dioxidation (M) [9]	2129,37	
1697	774,45	1546,88	1546,88	-0,93	1	87,26	158	172	LRTQVTGFLSGALGK	L	 	2052,72	
512	547,77	1093,53	1093,53	-1,07	0	60,53	94	103	MGVDHPLPGR	T	Oxidation (M) [1]	1192,47	
510	547,77	1093,53	1093,53	-2,55	0	34,47	94	103	MGVDHPLPGR	T	Oxidation (M) [1]	1160,02	
466	539,78	1077,54	1077,54	-2,38	0	50,15	94	103	MGVDHPLPGR	T	 	1218,50	
2463	952,44	1902,87	1902,87	-1,02	1	118,29	332	347	MHDKDDAFAAFHETLR	N	 	1689,39	
2459	476,72	1902,86	1902,87	-2,34	1	44,93	332	347	MHDKDDAFAAFHETLR	N	 	1862,39	
2462	635,30	1902,87	1902,87	-1,34	1	77,87	332	347	MHDKDDAFAAFHETLR	N	 	1682,83	
2465	476,72	1902,87	1902,87	-0,13	1	56,14	332	347	MHDKDDAFAAFHETLR	N	 	1680,43	
2496	640,63	1918,86	1918,86	-3,67	1	69,74	332	347	MHDKDDAFAAFHETLR	N	Oxidation (M) [1]	1640,68	
2497	480,72	1918,86	1918,86	-2,07	1	32,19	332	347	MHDKDDAFAAFHETLR	N	Oxidation (M) [1]	2188,12	
2498	480,72	1918,86	1918,86	-1,97	1	47,94	332	347	MHDKDDAFAAFHETLR	N	Oxidation (M) [1]	1635,32	
2499	960,44	1918,86	1918,86	-1,64	1	117,97	332	347	MHDKDDAFAAFHETLR	N	Oxidation (M) [1]	1640,58	
2500	480,72	1918,86	1918,86	-0,26	1	48,47	332	347	MHDKDDAFAAFHETLR	N	Oxidation (M) [1]	2006,37	
2502	480,72	1918,87	1918,86	1,06	1	43,00	332	347	MHDKDDAFAAFHETLR	N	Oxidation (M) [1]	1820,96	
2505	480,72	1918,87	1918,86	2,95	1	32,00	332	347	MHDKDDAFAAFHETLR	N	Oxidation (M) [1]	3028,82	
2535	645,96	1934,85	1934,86	-2,47	1	56,97	332	347	MHDKDDAFAAFHETLR	N	Dioxidation (M) [1]	1671,57	
2536	484,72	1934,85	1934,86	-2,36	1	47,04	332	347	MHDKDDAFAAFHETLR	N	Dioxidation (M) [1]	1667,60	
2555	649,30	1944,88	1944,88	-1,93	1	63,61	332	347	MHDKDDAFAAFHETLR	N	Acetyl (K) [4]	1830,86	
2556	649,30	1944,88	1944,88	-0,65	1	47,56	332	347	MHDKDDAFAAFHETLR	N	Acetyl (K) [4]	1807,79	
2557	487,23	1944,88	1944,88	-0,38	1	46,30	332	347	MHDKDDAFAAFHETLR	N	Acetyl (K) [4]	1831,76	
2559	487,23	1944,88	1944,88	0,38	1	46,86	332	347	MHDKDDAFAAFHETLR	N	Acetyl (K) [4]	1804,43	
2612	981,44	1960,87	1960,87	0,12	1	30,03	332	347	MHDKDDAFAAFHETLR	N	Oxidation (M) [1], Acetyl (K) [4]	1812,36	
2894	760,36	2278,07	2278,07	-0,09	0	89,22	209	228	NPGLNLVPLHMDMAEDEEVR	T	 	2319,65	
2892	760,36	2278,07	2278,07	-1,33	0	55,74	209	228	NPGLNLVPLHMDMAEDEEVR	T	 	2354,71	
2893	1140,04	2278,07	2278,07	-0,23	0	86,80	209	228	NPGLNLVPLHMDMAEDEEVR	T	 	2329,75	
2907	1148,04	2294,07	2294,07	-0,02	0	44,60	209	228	NPGLNLVPLHMDMAEDEEVR	T	Oxidation (M) [11]	2088,87	
2908	765,70	2294,07	2294,07	0,17	0	43,10	209	228	NPGLNLVPLHMDMAEDEEVR	T	Oxidation (M) [11]	2083,07	
2925	771,03	2310,06	2310,06	-0,24	0	51,21	209	228	NPGLNLVPLHMDMAEDEEVR	T	Oxidation (M) [11], Oxidation (M) [13]	1926,68	
2928	1156,04	2310,07	2310,06	1,75	0	37,96	209	228	NPGLNLVPLHMDMAEDEEVR	T	Oxidation (M) [11], Oxidation (M) [13]	1933,49	
3416	801,89	3203,52	3203,52	-0,18	1	43,33	209	237	NPGLNLVPLHMDMAEDEEVRTQPPMAGSR	H	 	2247,55	
3418	805,88	3219,51	3219,51	-0,72	1	38,26	209	237	NPGLNLVPLHMDMAEDEEVRTQPPMAGSR	H	Oxidation (M) [11]	2051,05	
3431	651,31	3251,50	3251,50	1,13	1	30,93	209	237	NPGLNLVPLHMDMAEDEEVRTQPPMAGSR	H	Dioxidation (M) [11], Oxidation (M) [25]	1842,67	
1254	679,34	1356,66	1356,66	-4,82	0	85,33	22	34	PSQTNADTTPLGR	R	 	1267,77	
1253	679,34	1356,66	1356,66	-5,23	0	64,49	22	34	PSQTNADTTPLGR	R	 	1206,07	
1252	679,34	1356,66	1356,66	-5,29	0	69,98	22	34	PSQTNADTTPLGR	R	 	1256,85	
1255	679,34	1356,66	1356,66	-1,45	0	56,12	22	34	PSQTNADTTPLGR	R	 	1399,36	
1256	679,34	1356,66	1356,66	-1,06	0	71,08	22	34	PSQTNADTTPLGR	R	 	1118,67	
1662	764,32	1526,63	1526,63	-0,99	0	73,32	53	65	QDSPEDSAQTMFR	R	Oxidation (M) [11]	1366,03	
1660	764,32	1526,62	1526,63	-4,66	0	48,80	53	65	QDSPEDSAQTMFR	R	Oxidation (M) [11]	1245,71	
1644	756,33	1510,64	1510,64	1,00	0	59,26	53	65	QDSPEDSAQTMFR	R	 	1738,84	
2237	604,60	1810,79	1810,79	-0,19	1	52,54	51	65	QRQDSPEDSAQTMFR	R	Oxidation (M) [13]	1340,04	
2199	898,41	1794,80	1794,80	0,95	1	41,69	51	65	QRQDSPEDSAQTMFR	R	 	1515,77	
2233	604,60	1810,78	1810,79	-5,32	1	50,21	51	65	QRQDSPEDSAQTMFR	R	Oxidation (M) [13]	1280,48	
2235	604,60	1810,79	1810,79	-2,32	1	35,40	51	65	QRQDSPEDSAQTMFR	R	Oxidation (M) [13]	1097,53	
2198	599,27	1794,79	1794,80	-0,31	1	51,42	51	65	QRQDSPEDSAQTMFR	R	 	1550,62	
3321	761,40	3041,57	3041,57	-0,40	2	82,48	66	93	RAGMTSLPPSPATSEHVPLLDNRPTLER	M	 	1870,72	
3323	761,40	3041,57	3041,57	0,13	2	31,74	66	93	RAGMTSLPPSPATSEHVPLLDNRPTLER	M	 	2008,10	
3330	765,40	3057,57	3057,57	1,08	2	56,60	66	93	RAGMTSLPPSPATSEHVPLLDNRPTLER	M	Oxidation (M) [4]	1748,68	
3332	1020,20	3057,57	3057,57	1,25	2	55,42	66	93	RAGMTSLPPSPATSEHVPLLDNRPTLER	M	Oxidation (M) [4]	1797,02	
3333	765,40	3057,57	3057,57	2,05	2	40,21	66	93	RAGMTSLPPSPATSEHVPLLDNRPTLER	M	Oxidation (M) [4]	1988,58	
1149	653,83	1305,64	1305,64	-3,65	1	39,12	37	49	RAPDDAPGSPPAR	R	 	913,48	
1152	653,83	1305,64	1305,64	-2,50	1	38,91	37	49	RAPDDAPGSPPAR	R	 	998,54	
1547	488,25	1461,74	1461,74	-3,67	2	39,25	37	50	RAPDDAPGSPPARR	Q	 	750,87	
1313	460,92	1379,72	1379,73	-2,58	1	43,70	133	146	SAGPATAARPQPTR	T	 	994,95	
1311	460,91	1379,72	1379,73	-4,10	1	42,45	133	146	SAGPATAARPQPTR	T	 	811,93	
1067	639,85	1277,69	1277,70	-2,99	0	63,33	160	172	TQVTGFLSGALGK	L	 	2097,41	
1068	639,85	1277,70	1277,70	-2,11	0	62,03	160	172	TQVTGFLSGALGK	L	 	2281,14	
1069	639,86	1277,70	1277,70	-0,30	0	62,97	160	172	TQVTGFLSGALGK	L	 	2465,93	
1071	639,86	1277,70	1277,70	1,33	0	34,46	160	172	TQVTGFLSGALGK	L	 	2650,81	
1073	639,86	1277,70	1277,70	1,97	0	34,92	160	172	TQVTGFLSGALGK	L	 	3022,21	
453	538,28	1074,54	1074,54	-4,25	0	67,88	147	157	TSAGQQATVGR	L	 	719,19	
454	538,28	1074,54	1074,54	-4,14	0	63,25	147	157	TSAGQQATVGR	L	 	1115,24	
455	538,28	1074,54	1074,54	-3,47	0	61,97	147	157	TSAGQQATVGR	L	 	902,72	
456	538,28	1074,54	1074,54	-3,19	0	66,33	147	157	TSAGQQATVGR	L	 	1086,62	
459	538,28	1074,55	1074,54	8,33	0	49,20	147	157	TSAGQQATVGR	L	 	1441,27	
1183	662,33	1322,64	1322,64	-1,44	0	88,68	119	132	TSTASAAQVASSSR	S	 	1002,58	
1177	662,33	1322,64	1322,64	-3,52	0	58,27	119	132	TSTASAAQVASSSR	S	 	1570,06	
1178	662,33	1322,64	1322,64	-3,46	0	31,11	119	132	TSTASAAQVASSSR	S	 	1754,57	
1179	662,33	1322,64	1322,64	-3,34	0	29,63	119	132	TSTASAAQVASSSR	S	 	3885,53	
1180	662,33	1322,64	1322,64	-2,22	0	48,40	119	132	TSTASAAQVASSSR	S	 	1669,43	
1182	662,33	1322,64	1322,64	-1,48	0	66,72	119	132	TSTASAAQVASSSR	S	 	1358,70	
1176	662,33	1322,64	1322,64	-3,83	0	76,24	119	132	TSTASAAQVASSSR	S	 	1175,05	
1188	662,33	1322,64	1322,64	0,94	0	38,66	119	132	TSTASAAQVASSSR	S	 	1985,44	
1190	662,33	1322,64	1322,64	1,19	0	40,13	119	132	TSTASAAQVASSSR	S	 	3692,20	
1191	662,33	1322,64	1322,64	1,25	0	48,23	119	132	TSTASAAQVASSSR	S	 	2000,77	
1192	662,33	1322,64	1322,64	1,36	0	82,50	119	132	TSTASAAQVASSSR	S	 	3369,44	
1194	662,33	1322,64	1322,64	1,59	0	28,70	119	132	TSTASAAQVASSSR	S	 	2610,24	
1195	662,33	1322,65	1322,64	2,00	0	30,14	119	132	TSTASAAQVASSSR	S	 	3619,86	
1197	662,33	1322,65	1322,64	2,31	0	70,18	119	132	TSTASAAQVASSSR	S	 	3378,41	
1198	662,33	1322,65	1322,64	2,31	0	62,74	119	132	TSTASAAQVASSSR	S	 	3405,08	
1201	662,33	1322,65	1322,64	3,33	0	58,29	119	132	TSTASAAQVASSSR	S	 	3184,25	
1982	854,90	1707,78	1707,79	-2,63	0	108,23	104	118	TWYETGHTTASLADR	T	 	1594,68	
1978	570,27	1707,77	1707,79	-6,10	0	30,74	104	118	TWYETGHTTASLADR	T	 	1583,75	
1983	570,27	1707,78	1707,79	-1,53	0	58,06	104	118	TWYETGHTTASLADR	T	 	1617,93	
1985	570,27	1707,78	1707,79	-0,25	0	32,90	104	118	TWYETGHTTASLADR	T	 	1765,32	
1990	854,90	1707,79	1707,79	0,45	0	74,18	104	118	TWYETGHTTASLADR	T	 	1783,56	
3315	1005,15	3012,42	3012,42	1,21	1	64,07	104	132	TWYETGHTTASLADRTSTASAAQVASSSR	S	 	1737,32	
2818	708,74	2123,18	2123,18	3,38	1	41,15	190	208	VLDVDRAIMPLLIVAENAR	N	Oxidation (M) [9]	2785,27	
2125	881,42	1760,83	1760,83	-0,80	0	88,66	3	21	VSSANAGVPASSADNTSAR	P	 	1085,56	
2118	881,42	1760,82	1760,83	-7,45	0	58,86	3	21	VSSANAGVPASSADNTSAR	P	 	1148,54	
2122	587,95	1760,82	1760,83	-3,12	0	36,88	3	21	VSSANAGVPASSADNTSAR	P	 	1070,40	
2119	881,42	1760,82	1760,83	-7,04	0	59,55	3	21	VSSANAGVPASSADNTSAR	P	 	1136,42	
2123	881,42	1760,82	1760,83	-3,11	0	73,32	3	21	VSSANAGVPASSADNTSAR	P	 	1063,36	
3359	1034,17	3099,49	3099,48	2,18	1	77,59	3	34	VSSANAGVPASSADNTSARPSQTNADTTPLGR	R	 	1411,01	
3357	775,88	3099,48	3099,48	0,95	1	44,82	3	34	VSSANAGVPASSADNTSARPSQTNADTTPLGR	R	 	1408,94	
3355	1034,17	3099,48	3099,48	-0,53	1	53,89	3	34	VSSANAGVPASSADNTSARPSQTNADTTPLGR	R	 	1447,40	
2661	662,00	1982,98	1982,98	-0,86	1	56,80	436	453	VTSGETKEITFSNSVEQK	R	 	1454,05	
2831	714,03	2139,08	2139,08	-1,75	2	64,13	436	454	VTSGETKEITFSNSVEQKR	I	 	1337,11	
1946	841,93	1681,84	1681,84	-0,99	0	57,44	394	408	YLGNHPEQSTVPVNK	R	 	1161,76	
1938	841,92	1681,83	1681,84	-7,10	0	39,61	394	408	YLGNHPEQSTVPVNK	R	 	1297,17	
1945	841,93	1681,84	1681,84	-1,16	0	40,84	394	408	YLGNHPEQSTVPVNK	R	 	1234,00	
2313	613,65	1837,94	1837,94	-3,19	1	63,32	394	409	YLGNHPEQSTVPVNKR	N	 	1040,58	
2314	613,65	1837,94	1837,94	-2,99	1	57,17	394	409	YLGNHPEQSTVPVNKR	N	 	1223,35	
2315	919,98	1837,94	1837,94	-1,87	1	58,07	394	409	YLGNHPEQSTVPVNKR	N	 	1047,17	
2317	613,65	1837,94	1837,94	-0,80	1	33,73	394	409	YLGNHPEQSTVPVNKR	N	 	1406,13	
1338	463,90	1388,67	1388,68	-3,39	1	44,18	251	262	YRAVIDDGSHTR	A	 	956,82	
