# Supplementary material for: Autoacetylation of the Ralstonia solanacearum Effector PopP2 Targets a Lysine Residue Essential for RRS1-R-Mediated Immunity in Arabidopsis
Source: PLoS Pathog. 2010 Nov 18;6(11):e1001202. doi: 10.1371/journal.ppat.1001202 (PMC2987829; doi:10.1371/journal.ppat.1001202)
Supplement: Table S3 — Liquid chromatography/tandem mass spectrometry (LC-MS/MS) analysis of GST-PopP2-K383R. (0.94 MB RTF) [file ppat.1001202.s006.rtf]

Description	accession	(a) Mass	(b) Coverage	(c) #peptides	 (d) emPAI	(e) SC (relevant+duplicated)							
PopP2-K383R	Q8Y125	52819,67	81,67	40	506,23	294							
(f) Query	(g) Observed	(h) Mr (expt)	(i) Mr (calc)	(j) delta	(k) miss	(l) score	(m) start	stop	(n) sequence		(o) modifications	(p) R.T.	
1828	787,90	1573,79	1573,79	0,73	0	68,76	461	475	AASYVNSAPPPVVMR	M	Oxidation (M) [14]	1559,51	
1780	779,91	1557,80	1557,80	0,05	0	51,98	461	475	AASYVNSAPPPVVMR	M		1780,28	
1815	787,90	1573,79	1573,79	-3,36	0	56,14	461	475	AASYVNSAPPPVVMR	M	Oxidation (M) [14]	1599,76	
1818	787,90	1573,79	1573,79	-2,01	0	66,21	461	475	AASYVNSAPPPVVMR	M	Oxidation (M) [14]	1683,98	
1819	787,90	1573,79	1573,79	-1,58	0	34,71	461	475	AASYVNSAPPPVVMR	M	Oxidation (M) [14]	1713,99	
1823	787,90	1573,79	1573,79	-0,18	0	51,91	461	475	AASYVNSAPPPVVMR	M	Oxidation (M) [14]	1897,52	
1825	525,60	1573,79	1573,79	0,24	0	52,03	461	475	AASYVNSAPPPVVMR	M	Oxidation (M) [14]	1561,82	
1776	779,90	1557,79	1557,80	-2,39	0	68,03	461	475	AASYVNSAPPPVVMR	M		1832,80	
1830	787,90	1573,79	1573,79	0,90	0	37,41	461	475	AASYVNSAPPPVVMR	M	Oxidation (M) [14]	1844,26	
1835	787,90	1573,79	1573,79	1,29	0	33,32	461	475	AASYVNSAPPPVVMR	M	Oxidation (M) [14]	2510,69	
1839	787,90	1573,79	1573,79	1,55	0	31,94	461	475	AASYVNSAPPPVVMR	M	Oxidation (M) [14]	4239,15	
3716	968,16	2901,47	2901,47	1,35	1	47,58	67	93	AGMTSLPPSPATSEHVPLLDNRPTLER	M	Oxidation (M) [3]	1964,99	
1361	705,91	1409,80	1409,81	-1,01	0	95,88	196	208	AIMPLLIVAENAR	N		2836,59	
1359	705,91	1409,80	1409,81	-2,87	0	83,88	196	208	AIMPLLIVAENAR	N		2814,79	
1364	705,91	1409,81	1409,81	-0,41	0	59,02	196	208	AIMPLLIVAENAR	N		3200,66	
1365	705,91	1409,81	1409,81	-0,29	0	55,12	196	208	AIMPLLIVAENAR	N		3566,05	
1366	705,91	1409,81	1409,81	-0,23	0	60,49	196	208	AIMPLLIVAENAR	N		3748,80	
1367	705,91	1409,81	1409,81	-0,17	0	51,99	196	208	AIMPLLIVAENAR	N		3383,68	
1368	705,91	1409,81	1409,81	-0,13	0	66,39	196	208	AIMPLLIVAENAR	N		3014,56	
1369	705,91	1409,81	1409,81	-0,03	0	57,31	196	208	AIMPLLIVAENAR	N		3930,54	
1370	705,91	1409,81	1409,81	0,37	0	48,29	196	208	AIMPLLIVAENAR	N		2627,97	
1374	705,91	1409,81	1409,81	0,89	0	56,83	196	208	AIMPLLIVAENAR	N		4113,05	
1376	705,91	1409,81	1409,81	1,29	0	58,74	196	208	AIMPLLIVAENAR	N		4296,09	
1377	705,91	1409,81	1409,81	1,39	0	43,83	196	208	AIMPLLIVAENAR	N		4700,17	
1416	713,91	1425,80	1425,80	-3,51	0	76,72	196	208	AIMPLLIVAENAR	N	Oxidation (M) [3]	2489,82	
1417	713,91	1425,80	1425,80	-2,79	0	51,68	196	208	AIMPLLIVAENAR	N	Oxidation (M) [3]	3509,59	
1418	713,91	1425,80	1425,80	-2,01	0	47,38	196	208	AIMPLLIVAENAR	N	Oxidation (M) [3]	4847,53	
1419	713,91	1425,80	1425,80	-1,47	0	74,47	196	208	AIMPLLIVAENAR	N	Oxidation (M) [3]	2774,76	
1420	713,91	1425,80	1425,80	-0,59	0	67,10	196	208	AIMPLLIVAENAR	N	Oxidation (M) [3]	3326,54	
1422	713,91	1425,80	1425,80	-0,21	0	71,91	196	208	AIMPLLIVAENAR	N	Oxidation (M) [3]	3139,66	
1423	713,91	1425,80	1425,80	-0,14	0	59,68	196	208	AIMPLLIVAENAR	N	Oxidation (M) [3]	3692,02	
1424	713,91	1425,80	1425,80	0,07	0	52,69	196	208	AIMPLLIVAENAR	N	Oxidation (M) [3]	3874,95	
1425	713,91	1425,80	1425,80	0,26	0	59,16	196	208	AIMPLLIVAENAR	N	Oxidation (M) [3]	4057,63	
1426	713,91	1425,80	1425,80	0,29	0	73,93	196	208	AIMPLLIVAENAR	N	Oxidation (M) [3]	2956,92	
1427	713,91	1425,80	1425,80	0,38	0	68,48	196	208	AIMPLLIVAENAR	N	Oxidation (M) [3]	2585,95	
1428	713,91	1425,80	1425,80	0,42	0	43,97	196	208	AIMPLLIVAENAR	N	Oxidation (M) [3]	4422,66	
1429	713,91	1425,80	1425,80	0,80	0	47,36	196	208	AIMPLLIVAENAR	N	Oxidation (M) [3]	4240,45	
1430	713,91	1425,80	1425,80	0,83	0	86,87	196	208	AIMPLLIVAENAR	N	Oxidation (M) [3]	2402,75	
1432	476,27	1425,80	1425,80	1,12	0	39,52	196	208	AIMPLLIVAENAR	N	Oxidation (M) [3]	2412,36	
1482	721,91	1441,80	1441,80	1,06	0	68,52	196	208	AIMPLLIVAENAR	N	Dioxidation (M) [3]	2547,41	
735	575,78	1149,54	1149,54	-3,86	0	49,97	38	49	APDDAPGSPPAR	R		918,99	
734	575,78	1149,54	1149,54	-4,26	0	42,61	38	49	APDDAPGSPPAR	R		1286,09	
736	575,78	1149,54	1149,54	-3,35	0	45,01	38	49	APDDAPGSPPAR	R		1101,78	
3191	756,40	2266,18	2266,18	1,95	0	88,52	359	380	AQQTEELGATLVLDGAPLVDAR	M		2727,09	
3177	1134,10	2266,18	2266,18	0,25	0	32,26	359	380	AQQTEELGATLVLDGAPLVDAR	M		3430,60	
3179	1134,10	2266,18	2266,18	0,60	0	32,69	359	380	AQQTEELGATLVLDGAPLVDAR	M		3517,63	
3172	1134,10	2266,18	2266,18	-1,16	0	58,80	359	380	AQQTEELGATLVLDGAPLVDAR	M		3292,13	
3182	756,40	2266,18	2266,18	0,83	0	35,88	359	380	AQQTEELGATLVLDGAPLVDAR	M		4130,59	
3183	1134,10	2266,18	2266,18	0,87	0	37,99	359	380	AQQTEELGATLVLDGAPLVDAR	M		3106,96	
3180	1134,10	2266,18	2266,18	0,60	0	70,21	359	380	AQQTEELGATLVLDGAPLVDAR	M		2913,11	
3187	1134,10	2266,18	2266,18	1,49	0	33,23	359	380	AQQTEELGATLVLDGAPLVDAR	M		3225,25	
3189	1134,10	2266,18	2266,18	1,75	0	30,83	359	380	AQQTEELGATLVLDGAPLVDAR	M		3872,87	
3186	756,40	2266,18	2266,18	1,22	0	77,88	359	380	AQQTEELGATLVLDGAPLVDAR	M		2542,00	
3192	1134,10	2266,19	2266,18	2,19	0	66,79	359	380	AQQTEELGATLVLDGAPLVDAR	M		2542,53	
3194	1134,10	2266,19	2266,18	2,63	0	78,02	359	380	AQQTEELGATLVLDGAPLVDAR	M		2727,46	
411	535,76	1069,51	1069,52	-3,92	0	67,42	253	262	AVIDDGSHTR	A		871,39	
408	535,76	1069,51	1069,52	-7,79	0	37,46	253	262	AVIDDGSHTR	A		1375,80	
410	535,76	1069,51	1069,52	-4,63	0	38,86	253	262	AVIDDGSHTR	A		1090,37	
409	535,76	1069,51	1069,52	-5,01	0	49,63	253	262	AVIDDGSHTR	A		1082,89	
412	535,76	1069,51	1069,52	-2,75	0	33,94	253	262	AVIDDGSHTR	A		689,08	
413	535,76	1069,51	1069,52	-1,34	0	50,98	253	262	AVIDDGSHTR	A		1335,87	
415	535,76	1069,52	1069,52	0,12	0	58,83	253	262	AVIDDGSHTR	A		1053,64	
416	535,77	1069,52	1069,52	0,68	0	33,38	253	262	AVIDDGSHTR	A		4341,74	
417	535,77	1069,52	1069,52	0,92	0	64,16	253	262	AVIDDGSHTR	A		1239,88	
1438	714,89	1427,76	1427,76	1,05	0	51,07	269	282	DASGTSVIVVDPLR	K		2256,07	
1775	778,94	1555,86	1555,86	2,09	1	62,53	269	283	DASGTSVIVVDPLRK	E		1927,91	
1300	464,89	1391,65	1391,65	1,03	0	46,21	336	347	DDAFAAFHETLR	N		2143,76	
1299	696,83	1391,65	1391,65	-0,28	0	43,95	336	347	DDAFAAFHETLR	N		2137,38	
3408	1210,50	2418,99	2418,99	1,34	0	91,36	286	306	DESAYVDYADNVNMEFGEHAK	C	Oxidation (M) [14]	2287,77	
3406	807,34	2418,99	2418,99	-2,22	0	42,78	286	306	DESAYVDYADNVNMEFGEHAK	C	Oxidation (M) [14]	2272,88	
3407	807,34	2418,99	2418,99	0,69	0	49,39	286	306	DESAYVDYADNVNMEFGEHAK	C	Oxidation (M) [14]	2287,70	
1036	641,32	1280,62	1280,62	-1,64	0	70,25	443	453	EITFSNSVEQK	R		1603,22	
1035	641,32	1280,62	1280,62	-5,09	0	60,46	443	453	EITFSNSVEQK	R		1626,87	
1038	641,32	1280,62	1280,62	-0,08	0	38,70	443	453	EITFSNSVEQK	R		2623,39	
1041	641,32	1280,63	1280,62	0,87	0	30,42	443	453	EITFSNSVEQK	R		1549,25	
1045	641,32	1280,63	1280,62	1,01	0	57,91	443	453	EITFSNSVEQK	R		1586,86	
1468	719,37	1436,72	1436,73	-3,89	1	72,75	443	454	EITFSNSVEQKR	I		1429,39	
1470	719,37	1436,73	1436,73	0,20	1	65,08	443	454	EITFSNSVEQKR	I		1397,67	
3489	887,72	2660,14	2660,13	1,24	1	127,76	284	306	EKDESAYVDYADNVNMEFGEHAK	C		2098,58	
3461	887,72	2660,13	2660,13	-1,14	1	33,91	284	306	EKDESAYVDYADNVNMEFGEHAK	C		3634,43	
3463	666,04	2660,13	2660,13	-0,49	1	30,98	284	306	EKDESAYVDYADNVNMEFGEHAK	C		3362,70	
3465	887,72	2660,13	2660,13	-0,22	1	33,38	284	306	EKDESAYVDYADNVNMEFGEHAK	C		3183,14	
3467	666,04	2660,13	2660,13	-0,08	1	54,30	284	306	EKDESAYVDYADNVNMEFGEHAK	C		3178,44	
3468	887,72	2660,13	2660,13	-0,08	1	54,55	284	306	EKDESAYVDYADNVNMEFGEHAK	C		3714,56	
3471	666,04	2660,13	2660,13	0,10	1	32,92	284	306	EKDESAYVDYADNVNMEFGEHAK	C		2402,75	
3473	887,72	2660,13	2660,13	0,13	1	37,94	284	306	EKDESAYVDYADNVNMEFGEHAK	C		3576,29	
3474	887,72	2660,13	2660,13	0,17	1	31,70	284	306	EKDESAYVDYADNVNMEFGEHAK	C		3781,13	
3475	887,72	2660,13	2660,13	0,22	1	47,27	284	306	EKDESAYVDYADNVNMEFGEHAK	C		3384,02	
3476	887,72	2660,13	2660,13	0,27	1	48,12	284	306	EKDESAYVDYADNVNMEFGEHAK	C		3924,69	
3478	887,72	2660,13	2660,13	0,31	1	51,13	284	306	EKDESAYVDYADNVNMEFGEHAK	C		3370,50	
3480	887,72	2660,13	2660,13	0,32	1	46,84	284	306	EKDESAYVDYADNVNMEFGEHAK	C		2514,87	
3482	887,72	2660,13	2660,13	0,45	1	32,84	284	306	EKDESAYVDYADNVNMEFGEHAK	C		2285,63	
3462	887,72	2660,13	2660,13	-1,01	1	61,32	284	306	EKDESAYVDYADNVNMEFGEHAK	C		2298,96	
3487	887,72	2660,14	2660,13	1,07	1	37,16	284	306	EKDESAYVDYADNVNMEFGEHAK	C		4164,45	
3486	666,04	2660,14	2660,13	0,89	1	61,43	284	306	EKDESAYVDYADNVNMEFGEHAK	C		2096,09	
3490	887,72	2660,14	2660,13	1,25	1	75,33	284	306	EKDESAYVDYADNVNMEFGEHAK	C		2994,02	
3491	887,72	2660,14	2660,13	1,94	1	34,54	284	306	EKDESAYVDYADNVNMEFGEHAK	C		4712,83	
3492	887,72	2660,14	2660,13	2,20	1	40,52	284	306	EKDESAYVDYADNVNMEFGEHAK	C		2808,60	
3523	893,05	2676,13	2676,13	-0,39	1	110,71	284	306	EKDESAYVDYADNVNMEFGEHAK	C	Oxidation (M) [16]	1767,81	
3534	670,04	2676,13	2676,13	0,38	1	41,16	284	306	EKDESAYVDYADNVNMEFGEHAK	C	Oxidation (M) [16]	2796,49	
3536	893,05	2676,13	2676,13	0,59	1	37,96	284	306	EKDESAYVDYADNVNMEFGEHAK	C	Oxidation (M) [16]	2874,25	
3544	893,05	2676,13	2676,13	1,35	1	112,39	284	306	EKDESAYVDYADNVNMEFGEHAK	C	Oxidation (M) [16]	2029,18	
3546	893,05	2676,13	2676,13	1,94	1	34,28	284	306	EKDESAYVDYADNVNMEFGEHAK	C	Oxidation (M) [16]	2241,27	
3548	893,05	2676,13	2676,13	2,08	1	33,87	284	306	EKDESAYVDYADNVNMEFGEHAK	C	Oxidation (M) [16]	2517,72	
3626	680,54	2718,14	2718,14	-0,26	1	53,53	284	306	EKDESAYVDYADNVNMEFGEHAK	C	Acetyl (K) [2], Oxidation (M) [16]	2164,93	
3627	907,05	2718,14	2718,14	0,96	1	81,14	284	306	EKDESAYVDYADNVNMEFGEHAK	C	Acetyl (K) [2], Oxidation (M) [16]	2161,59	
209	500,25	998,49	998,49	-3,47	0	57,24	384	393	HGQAASSVSR	Y		484,35	
205	500,25	998,48	998,49	-4,95	0	54,19	384	393	HGQAASSVSR	Y		301,70	
211	500,25	998,49	998,49	-3,31	0	41,49	384	393	HGQAASSVSR	Y		667,01	
556	550,80	1099,58	1099,58	0,19	0	65,69	238	247	HIAEFVASAR	P		1454,26	
544	550,79	1099,57	1099,58	-4,11	0	33,34	238	247	HIAEFVASAR	P		5019,66	
548	550,80	1099,58	1099,58	-1,01	0	36,72	238	247	HIAEFVASAR	P		3250,71	
552	550,80	1099,58	1099,58	-0,27	0	32,70	238	247	HIAEFVASAR	P		3616,24	
553	550,80	1099,58	1099,58	-0,23	0	52,67	238	247	HIAEFVASAR	P		1843,61	
554	550,80	1099,58	1099,58	-0,09	0	32,51	238	247	HIAEFVASAR	P		2508,64	
543	550,79	1099,57	1099,58	-4,18	0	55,32	238	247	HIAEFVASAR	P		1321,55	
560	550,80	1099,58	1099,58	0,55	0	54,06	238	247	HIAEFVASAR	P		1639,54	
561	550,80	1099,58	1099,58	0,99	0	45,36	238	247	HIAEFVASAR	P		2237,64	
1345	705,88	1409,75	1409,75	-2,52	1	72,59	238	250	HIAEFVASARPGR	Y		1303,38	
1353	470,92	1409,75	1409,75	0,23	1	30,62	238	250	HIAEFVASARPGR	Y		1727,63	
1355	470,93	1409,75	1409,75	0,44	1	34,95	238	250	HIAEFVASARPGR	Y		2480,13	
2079	842,98	1683,95	1683,95	-0,57	2	89,47	268	283	KDASGTSVIVVDPLRK	E		1616,39	
2077	562,32	1683,95	1683,95	-3,75	2	32,88	268	283	KDASGTSVIVVDPLRK	E		1724,43	
2076	562,32	1683,94	1683,95	-4,56	2	60,86	268	283	KDASGTSVIVVDPLRK	E		1658,50	
2078	562,32	1683,95	1683,95	-1,71	2	73,74	268	283	KDASGTSVIVVDPLRK	E		1610,15	
2080	562,33	1683,96	1683,95	1,95	2	57,01	268	283	KDASGTSVIVVDPLRK	E		1685,03	
2176	576,33	1725,96	1725,96	-2,78	2	52,29	268	283	KDASGTSVIVVDPLRK	E	Acetyl (K) [1]	1757,29	
3651	935,75	2804,23	2804,22	1,01	2	74,85	283	306	KEKDESAYVDYADNVNMEFGEHAK	C	Oxidation (M) [17]	1903,81	
3647	698,06	2788,23	2788,23	0,67	2	40,87	283	306	KEKDESAYVDYADNVNMEFGEHAK	C		1959,12	
3650	702,06	2804,22	2804,22	-0,66	2	48,46	283	306	KEKDESAYVDYADNVNMEFGEHAK	C	Oxidation (M) [17]	1890,12	
2687	966,49	1930,96	1930,96	1,74	0	117,15	173	189	LQALSAQNMDPELAQFR	V		2405,34	
2681	966,48	1930,95	1930,96	-2,06	0	78,01	173	189	LQALSAQNMDPELAQFR	V		2586,79	
2684	644,66	1930,96	1930,96	0,59	0	99,69	173	189	LQALSAQNMDPELAQFR	V		2407,60	
2737	649,99	1946,95	1946,95	-3,30	0	103,45	173	189	LQALSAQNMDPELAQFR	V	Oxidation (M) [9]	2201,19	
2742	649,99	1946,95	1946,95	-1,66	0	74,01	173	189	LQALSAQNMDPELAQFR	V	Oxidation (M) [9]	2180,31	
2747	974,48	1946,95	1946,95	-0,97	0	31,35	173	189	LQALSAQNMDPELAQFR	V	Oxidation (M) [9]	3725,30	
2750	649,99	1946,95	1946,95	-0,82	0	46,22	173	189	LQALSAQNMDPELAQFR	V	Oxidation (M) [9]	3934,50	
2753	974,48	1946,95	1946,95	-0,68	0	31,22	173	189	LQALSAQNMDPELAQFR	V	Oxidation (M) [9]	4363,47	
2754	649,99	1946,95	1946,95	-0,65	0	47,25	173	189	LQALSAQNMDPELAQFR	V	Oxidation (M) [9]	3212,08	
2758	649,99	1946,95	1946,95	-0,49	0	35,20	173	189	LQALSAQNMDPELAQFR	V	Oxidation (M) [9]	4168,87	
2767	649,99	1946,95	1946,95	-0,08	0	43,04	173	189	LQALSAQNMDPELAQFR	V	Oxidation (M) [9]	3974,52	
2773	974,48	1946,95	1946,95	0,17	0	45,81	173	189	LQALSAQNMDPELAQFR	V	Oxidation (M) [9]	2487,79	
2776	974,48	1946,95	1946,95	0,55	0	31,50	173	189	LQALSAQNMDPELAQFR	V	Oxidation (M) [9]	4048,37	
2777	974,48	1946,95	1946,95	0,57	0	30,54	173	189	LQALSAQNMDPELAQFR	V	Oxidation (M) [9]	3655,86	
2780	974,48	1946,95	1946,95	0,61	0	32,09	173	189	LQALSAQNMDPELAQFR	V	Oxidation (M) [9]	3164,93	
2782	649,99	1946,95	1946,95	0,72	0	45,60	173	189	LQALSAQNMDPELAQFR	V	Oxidation (M) [9]	2313,95	
2785	974,48	1946,95	1946,95	1,01	0	87,96	173	189	LQALSAQNMDPELAQFR	V	Oxidation (M) [9]	2304,07	
2788	649,99	1946,95	1946,95	1,29	0	46,14	173	189	LQALSAQNMDPELAQFR	V	Oxidation (M) [9]	4157,60	
2789	974,48	1946,95	1946,95	1,43	0	112,43	173	189	LQALSAQNMDPELAQFR	V	Oxidation (M) [9]	2120,87	
2790	974,48	1946,95	1946,95	1,46	0	53,41	173	189	LQALSAQNMDPELAQFR	V	Oxidation (M) [9]	4302,64	
2792	974,48	1946,96	1946,95	1,67	0	58,28	173	189	LQALSAQNMDPELAQFR	V	Oxidation (M) [9]	4276,22	
2794	649,99	1946,96	1946,95	1,79	0	93,61	173	189	LQALSAQNMDPELAQFR	V	Oxidation (M) [9]	2120,34	
2797	974,49	1946,96	1946,95	2,78	0	36,88	173	189	LQALSAQNMDPELAQFR	V	Oxidation (M) [9]	4112,76	
2798	974,49	1946,96	1946,95	2,81	0	32,49	173	189	LQALSAQNMDPELAQFR	V	Oxidation (M) [9]	2975,32	
2799	974,49	1946,96	1946,95	2,90	0	49,65	173	189	LQALSAQNMDPELAQFR	V	Oxidation (M) [9]	2924,47	
2800	974,49	1946,96	1946,95	3,31	0	45,51	173	189	LQALSAQNMDPELAQFR	V	Oxidation (M) [9]	4058,77	
2803	974,49	1946,96	1946,95	5,78	0	50,57	173	189	LQALSAQNMDPELAQFR	V	Oxidation (M) [9]	4261,46	
2854	655,32	1962,95	1962,95	0,73	0	77,20	173	189	LQALSAQNMDPELAQFR	V	Dioxidation (M) [9]	2223,51	
2855	982,48	1962,95	1962,95	1,48	0	66,51	173	189	LQALSAQNMDPELAQFR	V	Dioxidation (M) [9]	2220,80	
1742	774,45	1546,88	1546,88	0,76	1	95,09	158	172	LRTQVTGFLSGALGK	L		2127,17	
1740	516,63	1546,88	1546,88	-0,56	1	68,91	158	172	LRTQVTGFLSGALGK	L		2124,64	
438	539,77	1077,53	1077,54	-4,70	0	52,75	94	103	MGVDHPLPGR	T		1279,44	
439	539,78	1077,54	1077,54	-0,32	0	34,25	94	103	MGVDHPLPGR	T		1262,87	
488	547,77	1093,53	1093,53	-1,30	0	49,81	94	103	MGVDHPLPGR	T	Oxidation (M) [1]	1133,12	
489	547,77	1093,53	1093,53	0,30	0	30,23	94	103	MGVDHPLPGR	T	Oxidation (M) [1]	1398,00	
2574	952,44	1902,87	1902,87	-0,47	1	135,77	332	347	MHDKDDAFAAFHETLR	N		1753,00	
2568	635,30	1902,87	1902,87	-1,29	1	88,85	332	347	MHDKDDAFAAFHETLR	N		1746,80	
2578	476,72	1902,87	1902,87	-0,03	1	54,66	332	347	MHDKDDAFAAFHETLR	N		1745,39	
2580	476,72	1902,87	1902,87	0,33	1	35,74	332	347	MHDKDDAFAAFHETLR	N		2159,26	
2584	476,72	1902,87	1902,87	1,27	1	34,43	332	347	MHDKDDAFAAFHETLR	N		1971,39	
2637	640,63	1918,86	1918,86	-2,02	1	73,73	332	347	MHDKDDAFAAFHETLR	N	Oxidation (M) [1]	1700,89	
2638	480,72	1918,86	1918,86	-1,95	1	46,76	332	347	MHDKDDAFAAFHETLR	N	Oxidation (M) [1]	1689,91	
2650	960,44	1918,86	1918,86	0,08	1	110,41	332	347	MHDKDDAFAAFHETLR	N	Oxidation (M) [1]	1699,20	
2655	480,72	1918,87	1918,86	1,12	1	35,94	332	347	MHDKDDAFAAFHETLR	N	Oxidation (M) [1]	2252,14	
2697	645,96	1934,85	1934,86	-3,99	1	59,07	332	347	MHDKDDAFAAFHETLR	N	Dioxidation (M) [1]	1730,25	
2698	484,72	1934,85	1934,86	-2,31	1	56,63	332	347	MHDKDDAFAAFHETLR	N	Dioxidation (M) [1]	1728,43	
2726	649,30	1944,87	1944,88	-2,13	1	52,88	332	347	MHDKDDAFAAFHETLR	N	Acetyl (K) [4]	1905,76	
2727	487,23	1944,88	1944,88	-0,32	1	62,36	332	347	MHDKDDAFAAFHETLR	N	Acetyl (K) [4]	1912,47	
2728	649,30	1944,88	1944,88	0,31	1	56,30	332	347	MHDKDDAFAAFHETLR	N	Acetyl (K) [4]	1883,63	
2849	491,22	1960,87	1960,87	-1,50	1	36,19	332	347	MHDKDDAFAAFHETLR	N	Oxidation (M) [1], Acetyl (K) [4]	1783,58	
2852	981,45	1960,88	1960,87	1,51	1	50,61	332	347	MHDKDDAFAAFHETLR	N	Oxidation (M) [1], Acetyl (K) [4]	1894,39	
3210	1140,04	2278,07	2278,07	0,30	0	111,87	209	228	NPGLNLVPLHMDMAEDEEVR	T		2423,97	
3211	760,37	2278,07	2278,07	0,78	0	92,84	209	228	NPGLNLVPLHMDMAEDEEVR	T		2413,04	
3224	765,69	2294,06	2294,07	-2,18	0	75,56	209	228	NPGLNLVPLHMDMAEDEEVR	T	Oxidation (M) [11]	2174,56	
3225	1148,04	2294,06	2294,07	-1,50	0	46,80	209	228	NPGLNLVPLHMDMAEDEEVR	T	Oxidation (M) [11]	2177,69	
3228	765,70	2294,07	2294,07	0,47	0	73,47	209	228	NPGLNLVPLHMDMAEDEEVR	T	Oxidation (M) [11]	2190,16	
3257	771,03	2310,06	2310,06	-0,36	0	44,52	209	228	NPGLNLVPLHMDMAEDEEVR	T	Oxidation (M) [11], Oxidation (M) [13]	2198,90	
3262	771,03	2310,06	2310,06	0,06	0	54,55	209	228	NPGLNLVPLHMDMAEDEEVR	T	Oxidation (M) [11], Oxidation (M) [13]	2509,14	
3264	771,03	2310,06	2310,06	0,13	0	30,83	209	228	NPGLNLVPLHMDMAEDEEVR	T	Dioxidation (M) [13]	3171,29	
3266	771,03	2310,06	2310,06	0,27	0	31,41	209	228	NPGLNLVPLHMDMAEDEEVR	T	Dioxidation (M) [13]	4014,95	
3269	771,03	2310,06	2310,06	0,29	0	56,56	209	228	NPGLNLVPLHMDMAEDEEVR	T	Oxidation (M) [11], Oxidation (M) [13]	2013,51	
3274	771,03	2310,06	2310,06	0,87	0	36,85	209	228	NPGLNLVPLHMDMAEDEEVR	T	Oxidation (M) [11], Oxidation (M) [13]	4208,57	
3858	1068,85	3203,52	3203,52	2,35	1	56,23	209	237	NPGLNLVPLHMDMAEDEEVRTQPPMAGSR	H		2330,02	
3857	801,89	3203,52	3203,52	0,70	1	49,25	209	237	NPGLNLVPLHMDMAEDEEVRTQPPMAGSR	H		2322,49	
3860	805,89	3219,51	3219,51	0,19	1	45,13	209	237	NPGLNLVPLHMDMAEDEEVRTQPPMAGSR	H	Oxidation (M) [11]	2136,03	
3861	805,89	3219,52	3219,51	1,73	1	46,77	209	237	NPGLNLVPLHMDMAEDEEVRTQPPMAGSR	H	Oxidation (M) [25]	2250,59	
3869	813,88	3251,49	3251,50	-2,68	1	30,56	209	237	NPGLNLVPLHMDMAEDEEVRTQPPMAGSR	H	Oxidation (M) [11], Oxidation (M) [13], Oxidation (M) [25]	1943,13	
1190	679,34	1356,66	1356,66	-1,14	0	79,91	22	34	PSQTNADTTPLGR	R		1399,10	
1187	679,34	1356,66	1356,66	-3,25	0	57,36	22	34	PSQTNADTTPLGR	R		1335,19	
1188	679,34	1356,66	1356,66	-1,87	0	64,69	22	34	PSQTNADTTPLGR	R		1418,80	
1189	453,23	1356,66	1356,66	-1,21	0	32,98	22	34	PSQTNADTTPLGR	R		1197,16	
1186	679,34	1356,66	1356,66	-4,43	0	76,21	22	34	PSQTNADTTPLGR	R		1311,04	
1191	679,34	1356,66	1356,66	-0,16	0	63,83	22	34	PSQTNADTTPLGR	R		1160,82	
1691	764,32	1526,63	1526,63	1,37	0	65,26	53	65	QDSPEDSAQTMFR	R	Oxidation (M) [11]	1484,61	
1688	764,32	1526,63	1526,63	-2,77	0	39,80	53	65	QDSPEDSAQTMFR	R	Oxidation (M) [11]	1299,98	
1640	756,33	1510,64	1510,64	1,46	0	60,97	53	65	QDSPEDSAQTMFR	R		1825,62	
1733	772,32	1542,63	1542,63	0,91	0	39,25	53	65	QDSPEDSAQTMFR	R	Dioxidation (M) [11]	1536,12	
2310	599,27	1794,79	1794,80	-0,74	1	64,04	51	65	QRQDSPEDSAQTMFR	R		1606,43	
2340	604,60	1810,78	1810,79	-4,18	1	55,72	51	65	QRQDSPEDSAQTMFR	R	Oxidation (M) [13]	1306,67	
2348	604,60	1810,79	1810,79	-1,10	1	49,24	51	65	QRQDSPEDSAQTMFR	R	Oxidation (M) [13]	1384,73	
2354	604,60	1810,79	1810,79	-0,34	1	51,58	51	65	QRQDSPEDSAQTMFR	R	Oxidation (M) [13]	1146,91	
3765	761,40	3041,57	3041,57	0,39	2	101,29	66	93	RAGMTSLPPSPATSEHVPLLDNRPTLER	M		1944,78	
3764	761,40	3041,57	3041,57	0,02	2	69,59	66	93	RAGMTSLPPSPATSEHVPLLDNRPTLER	M		2014,66	
3766	761,40	3041,58	3041,57	1,22	2	61,99	66	93	RAGMTSLPPSPATSEHVPLLDNRPTLER	M		1934,65	
3768	1014,87	3041,58	3041,57	2,78	2	45,48	66	93	RAGMTSLPPSPATSEHVPLLDNRPTLER	M		1970,74	
3772	765,40	3057,57	3057,57	0,27	2	55,12	66	93	RAGMTSLPPSPATSEHVPLLDNRPTLER	M	Oxidation (M) [4]	2005,13	
3776	1020,20	3057,57	3057,57	1,55	2	54,13	66	93	RAGMTSLPPSPATSEHVPLLDNRPTLER	M	Oxidation (M) [4]	1867,73	
3777	765,40	3057,57	3057,57	2,74	2	64,06	66	93	RAGMTSLPPSPATSEHVPLLDNRPTLER	M	Oxidation (M) [4]	1816,83	
1086	653,83	1305,64	1305,64	-3,46	1	55,91	37	49	RAPDDAPGSPPAR	R		972,47	
1085	653,83	1305,64	1305,64	-4,26	1	34,34	37	49	RAPDDAPGSPPAR	R		790,42	
1089	653,83	1305,64	1305,64	-0,34	1	42,61	37	49	RAPDDAPGSPPAR	R		1154,56	
161	487,75	973,49	973,49	-3,38	1	51,35	409	416	RNETLGER	T		606,45	
1265	460,92	1379,72	1379,73	-2,58	1	42,22	133	146	SAGPATAARPQPTR	T		947,23	
1020	639,86	1277,70	1277,70	-1,31	0	81,23	160	172	TQVTGFLSGALGK	L		2188,07	
1019	639,86	1277,70	1277,70	-1,57	0	74,73	160	172	TQVTGFLSGALGK	L		2372,22	
1021	639,86	1277,70	1277,70	-0,94	0	53,73	160	172	TQVTGFLSGALGK	L		3292,44	
1022	639,86	1277,70	1277,70	-0,58	0	58,07	160	172	TQVTGFLSGALGK	L		3108,77	
1023	639,86	1277,70	1277,70	-0,49	0	41,11	160	172	TQVTGFLSGALGK	L		3475,54	
1024	639,86	1277,70	1277,70	-0,45	0	41,69	160	172	TQVTGFLSGALGK	L		3658,20	
1025	639,86	1277,70	1277,70	-0,44	0	59,51	160	172	TQVTGFLSGALGK	L		2924,41	
1026	639,86	1277,70	1277,70	-0,28	0	41,50	160	172	TQVTGFLSGALGK	L		3841,63	
1027	639,86	1277,70	1277,70	0,02	0	36,13	160	172	TQVTGFLSGALGK	L		4024,39	
1028	639,86	1277,70	1277,70	0,25	0	33,44	160	172	TQVTGFLSGALGK	L		4389,87	
1030	639,86	1277,70	1277,70	0,75	0	64,07	160	172	TQVTGFLSGALGK	L		2739,97	
1031	639,86	1277,70	1277,70	0,89	0	41,29	160	172	TQVTGFLSGALGK	L		4207,49	
1032	639,86	1277,70	1277,70	1,72	0	80,40	160	172	TQVTGFLSGALGK	L		2556,76	
425	538,28	1074,54	1074,54	-4,68	0	80,90	147	157	TSAGQQATVGR	L		745,42	
426	538,28	1074,54	1074,54	-4,08	0	47,80	147	157	TSAGQQATVGR	L		563,35	
427	538,28	1074,54	1074,54	-3,64	0	62,59	147	157	TSAGQQATVGR	L		928,37	
428	538,28	1074,54	1074,54	-3,43	0	66,74	147	157	TSAGQQATVGR	L		1110,90	
429	538,28	1074,54	1074,54	-2,41	0	52,83	147	157	TSAGQQATVGR	L		1145,02	
431	538,28	1074,54	1074,54	-0,62	0	69,23	147	157	TSAGQQATVGR	L		1383,97	
432	538,28	1074,54	1074,54	-0,51	0	45,87	147	157	TSAGQQATVGR	L		4292,48	
1119	662,33	1322,64	1322,64	-3,52	0	101,25	119	132	TSTASAAQVASSSR	S		1305,95	
1118	662,33	1322,64	1322,64	-4,75	0	84,46	119	132	TSTASAAQVASSSR	S		1705,45	
1120	662,33	1322,64	1322,64	-3,19	0	53,91	119	132	TSTASAAQVASSSR	S		1732,47	
1121	662,33	1322,64	1322,64	-2,78	0	98,31	119	132	TSTASAAQVASSSR	S		961,34	
1122	662,33	1322,64	1322,64	-2,59	0	90,04	119	132	TSTASAAQVASSSR	S		1325,85	
1123	662,33	1322,64	1322,64	-1,60	0	34,98	119	132	TSTASAAQVASSSR	S		4856,67	
1124	662,33	1322,64	1322,64	-1,44	0	41,76	119	132	TSTASAAQVASSSR	S		3457,63	
1125	662,33	1322,64	1322,64	-0,86	0	41,54	119	132	TSTASAAQVASSSR	S		1989,20	
1126	662,33	1322,64	1322,64	-0,62	0	53,11	119	132	TSTASAAQVASSSR	S		3408,40	
1127	662,33	1322,64	1322,64	-0,51	0	49,91	119	132	TSTASAAQVASSSR	S		3826,27	
1128	662,33	1322,64	1322,64	-0,38	0	90,95	119	132	TSTASAAQVASSSR	S		1143,32	
1129	662,33	1322,64	1322,64	-0,32	0	41,31	119	132	TSTASAAQVASSSR	S		3642,80	
1130	662,33	1322,64	1322,64	-0,11	0	69,07	119	132	TSTASAAQVASSSR	S		3226,09	
1131	662,33	1322,64	1322,64	-0,11	0	57,22	119	132	TSTASAAQVASSSR	S		1512,67	
1132	662,33	1322,64	1322,64	0,23	0	53,66	119	132	TSTASAAQVASSSR	S		2852,37	
1133	662,33	1322,64	1322,64	0,26	0	44,70	119	132	TSTASAAQVASSSR	S		4009,54	
1135	662,33	1322,64	1322,64	0,60	0	54,44	119	132	TSTASAAQVASSSR	S		4376,83	
1138	662,33	1322,64	1322,64	0,94	0	58,59	119	132	TSTASAAQVASSSR	S		4191,74	
1139	662,33	1322,64	1322,64	0,97	0	50,00	119	132	TSTASAAQVASSSR	S		3039,09	
1140	662,33	1322,64	1322,64	1,53	0	46,83	119	132	TSTASAAQVASSSR	S		2474,07	
2089	854,90	1707,78	1707,79	-2,82	0	97,44	104	118	TWYETGHTTASLADR	T		1663,91	
2092	854,90	1707,78	1707,79	-1,33	0	59,01	104	118	TWYETGHTTASLADR	T		4300,89	
2098	854,90	1707,78	1707,79	-0,72	0	35,28	104	118	TWYETGHTTASLADR	T		3579,54	
2102	854,90	1707,78	1707,79	-0,44	0	52,81	104	118	TWYETGHTTASLADR	T		1799,44	
2127	854,90	1707,79	1707,79	0,85	0	40,84	104	118	TWYETGHTTASLADR	T		2073,72	
2128	854,90	1707,79	1707,79	0,88	0	86,62	104	118	TWYETGHTTASLADR	T		1846,89	
2138	854,90	1707,79	1707,79	1,55	0	40,03	104	118	TWYETGHTTASLADR	T		2248,05	
2140	854,90	1707,79	1707,79	1,67	0	47,49	104	118	TWYETGHTTASLADR	T		2332,00	
3100	703,40	2107,18	2107,18	0,60	1	44,23	190	208	VLDVDRAIMPLLIVAENAR	N		3093,57	
3099	703,40	2107,18	2107,18	0,44	1	32,17	190	208	VLDVDRAIMPLLIVAENAR	N		3105,81	
3108	708,73	2123,18	2123,18	0,45	1	34,72	190	208	VLDVDRAIMPLLIVAENAR	N	Oxidation (M) [9]	2888,31	
2254	881,42	1760,83	1760,83	0,38	0	76,60	3	21	VSSANAGVPASSADNTSAR	P		1134,70	
2249	587,95	1760,82	1760,83	-3,41	0	30,73	3	21	VSSANAGVPASSADNTSAR	P		1119,16	
2250	881,42	1760,82	1760,83	-2,27	0	50,21	3	21	VSSANAGVPASSADNTSAR	P		1297,91	
2251	881,42	1760,83	1760,83	-1,70	0	62,20	3	21	VSSANAGVPASSADNTSAR	P		1108,88	
2252	587,95	1760,83	1760,83	-0,04	0	56,96	3	21	VSSANAGVPASSADNTSAR	P		1133,58	
2248	881,42	1760,82	1760,83	-6,18	0	68,11	3	21	VSSANAGVPASSADNTSAR	P		1215,14	
2253	587,95	1760,83	1760,83	0,04	0	71,87	3	21	VSSANAGVPASSADNTSAR	P		1139,45	
3804	1034,17	3099,48	3099,48	0,44	1	67,80	3	34	VSSANAGVPASSADNTSARPSQTNADTTPLGR	R		1516,86	
3803	775,88	3099,48	3099,48	-1,02	1	39,23	3	34	VSSANAGVPASSADNTSARPSQTNADTTPLGR	R		1521,98	
3802	1034,17	3099,48	3099,48	-2,08	1	53,80	3	34	VSSANAGVPASSADNTSARPSQTNADTTPLGR	R		1731,36	
3807	1034,17	3099,49	3099,48	2,86	1	49,84	3	34	VSSANAGVPASSADNTSARPSQTNADTTPLGR	R		1488,42	
3808	775,88	3099,49	3099,48	2,91	1	47,48	3	34	VSSANAGVPASSADNTSARPSQTNADTTPLGR	R		1490,71	
2062	841,93	1681,84	1681,84	-0,16	0	54,93	394	408	YLGNHPEQSTVPVNK	R		1211,61	
2055	841,93	1681,84	1681,84	-1,17	0	37,18	394	408	YLGNHPEQSTVPVNK	R		1324,68	
2050	841,93	1681,84	1681,84	-3,14	0	53,31	394	408	YLGNHPEQSTVPVNK	R		1313,26	
2429	613,65	1837,94	1837,94	-1,90	1	74,51	394	409	YLGNHPEQSTVPVNKR	N		1265,66	
2427	613,65	1837,94	1837,94	-3,55	1	38,62	394	409	YLGNHPEQSTVPVNKR	N		1326,19	
2428	613,65	1837,94	1837,94	-2,21	1	55,62	394	409	YLGNHPEQSTVPVNKR	N		1070,29	
2430	919,98	1837,94	1837,94	-1,26	1	63,26	394	409	YLGNHPEQSTVPVNKR	N		1076,63	
2442	613,66	1837,94	1837,94	0,65	1	40,69	394	409	YLGNHPEQSTVPVNKR	N		1448,39	
