# Supplementary material for: Correction: Autoacetylation of the Ralstonia solanacearum Effector PopP2 Targets a Lysine Residue Essential for RRS1-R-Mediated Immunity in Arabidopsis
Source: PLoS Pathog. 2022 Mar 2;18(3):e1010368. doi: 10.1371/journal.ppat.1010368 (PMC8890644; doi:10.1371/journal.ppat.1010368)
Supplement: S1 File — (PPTX) [file ppat.1010368.s001.pptx]

## Slide 1
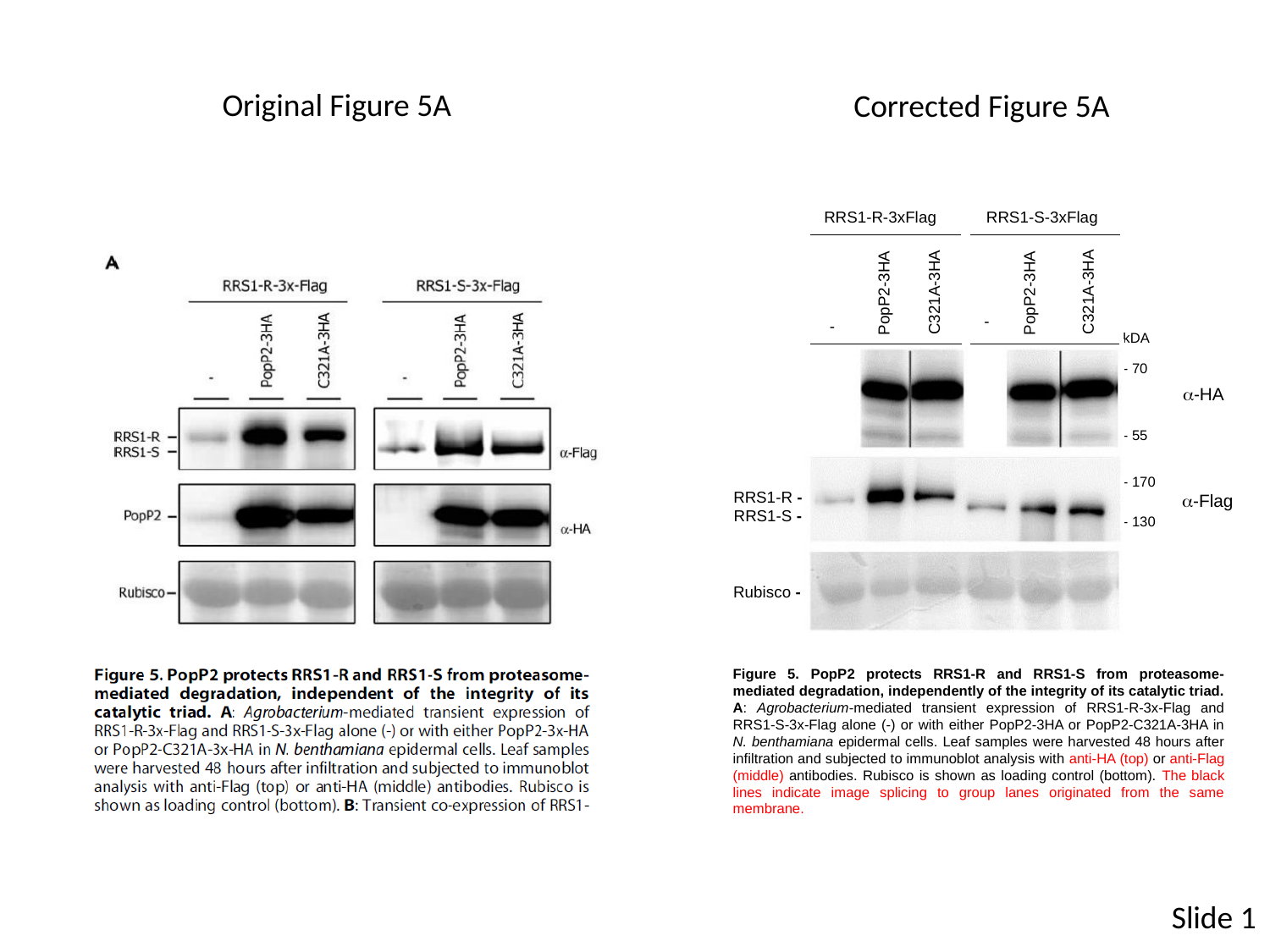

Original Figure 5A
Corrected Figure 5A
RRS1-S-3xFlag
RRS1-R-3xFlag
C321A-3HA
C321A-3HA
PopP2-3HA
PopP2-3HA
-
-
kDA
- 70
a-HA
- 55
- 170
RRS1-R -
a-Flag
RRS1-S -
- 130
Rubisco -
Figure 5. PopP2 protects RRS1-R and RRS1-S from proteasome-mediated degradation, independently of the integrity of its catalytic triad. A: Agrobacterium-mediated transient expression of RRS1-R-3x-Flag and RRS1-S-3x-Flag alone (-) or with either PopP2-3HA or PopP2-C321A-3HA in N. benthamiana epidermal cells. Leaf samples were harvested 48 hours after infiltration and subjected to immunoblot analysis with anti-HA (top) or anti-Flag (middle) antibodies. Rubisco is shown as loading control (bottom). The black lines indicate image splicing to group lanes originated from the same membrane.
Slide 1

## Slide 2
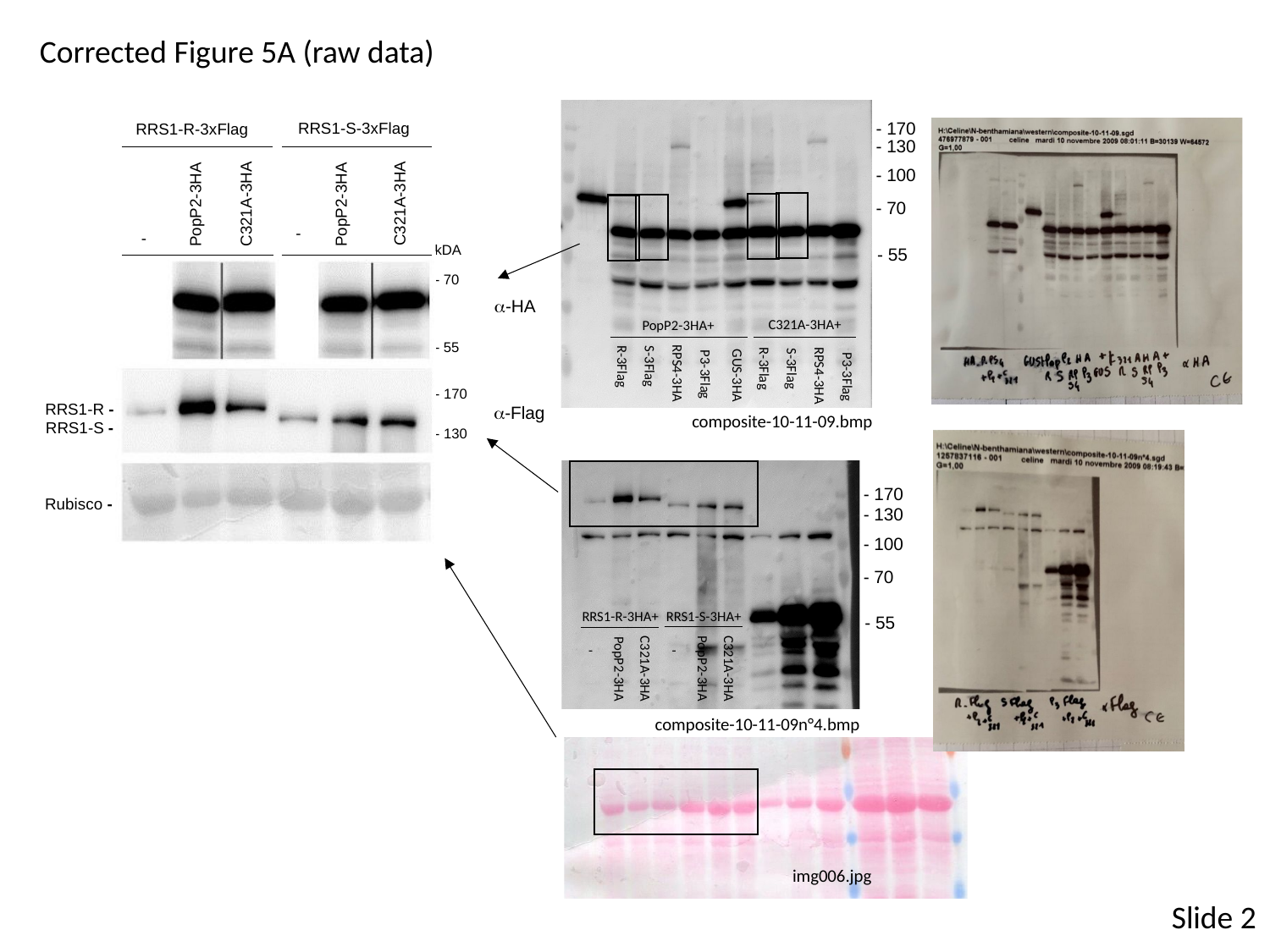

Corrected Figure 5A (raw data)
- 170
RRS1-S-3xFlag
RRS1-R-3xFlag
- 130
- 100
C321A-3HA
C321A-3HA
PopP2-3HA
PopP2-3HA
- 70
-
-
kDA
- 55
- 70
a-HA
C321A-3HA+
PopP2-3HA+
- 55
R-3Flag
S-3Flag
R-3Flag
S-3Flag
RPS4-3HA
P3-3Flag
GUS-3HA
RPS4-3HA
P3-3Flag
- 170
RRS1-R -
a-Flag
composite-10-11-09.bmp
RRS1-S -
- 130
- 170
- 130
- 100
- 70
- 55
composite-10-11-09n°4.bmp
Rubisco -
RRS1-S-3HA+
RRS1-R-3HA+
-
-
PopP2-3HA
C321A-3HA
PopP2-3HA
C321A-3HA
img006.jpg
Slide 2

## Slide 3
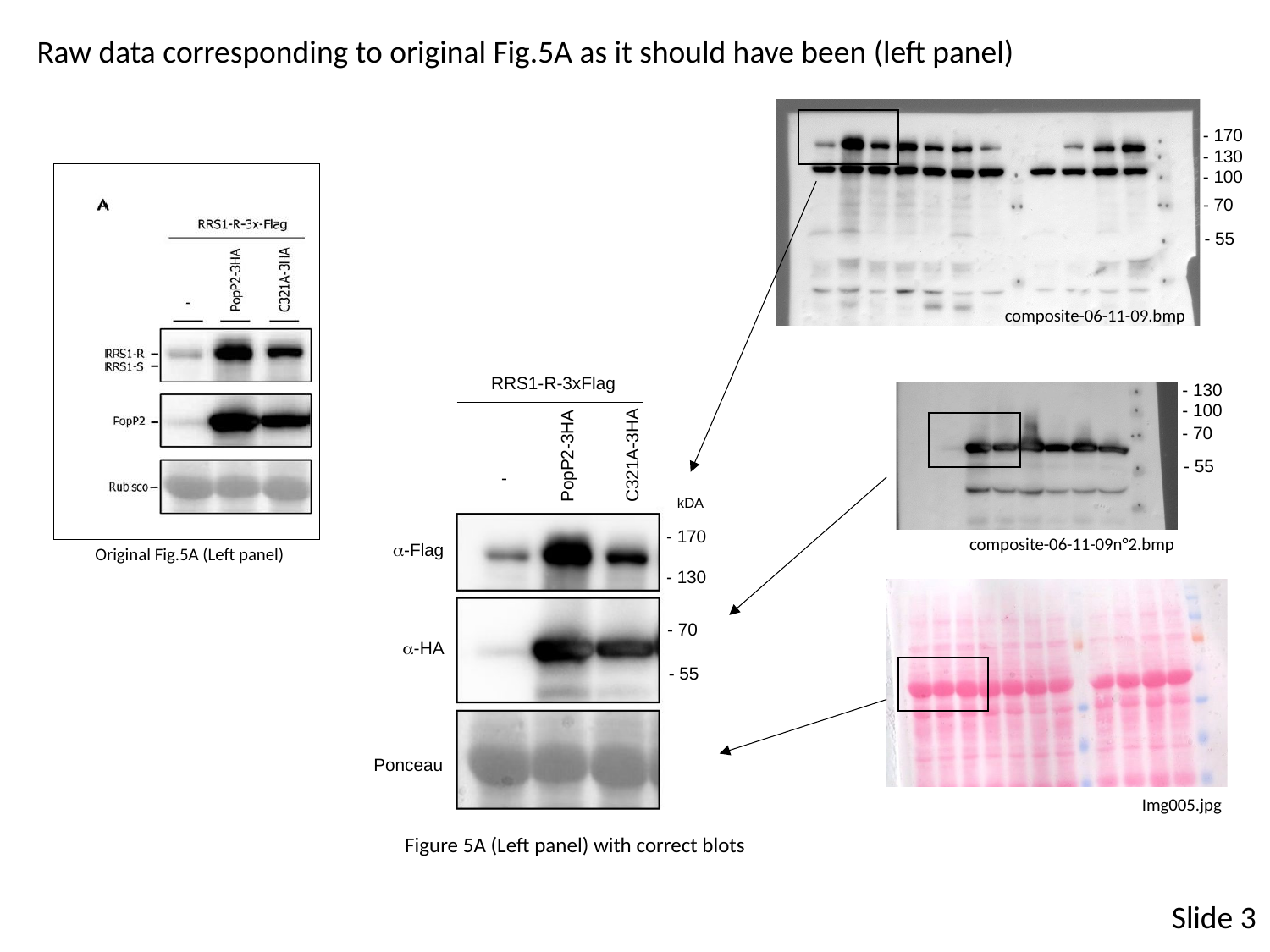

Raw data corresponding to original Fig.5A as it should have been (left panel)
- 170
- 130
- 100
- 70
- 55
composite-06-11-09.bmp
RRS1-R-3xFlag
- 130
- 100
- 70
C321A-3HA
PopP2-3HA
- 55
-
kDA
- 170
composite-06-11-09n°2.bmp
a-Flag
Original Fig.5A (Left panel)
- 130
- 70
a-HA
- 55
Ponceau
Img005.jpg
Figure 5A (Left panel) with correct blots
Slide 3

## Slide 4
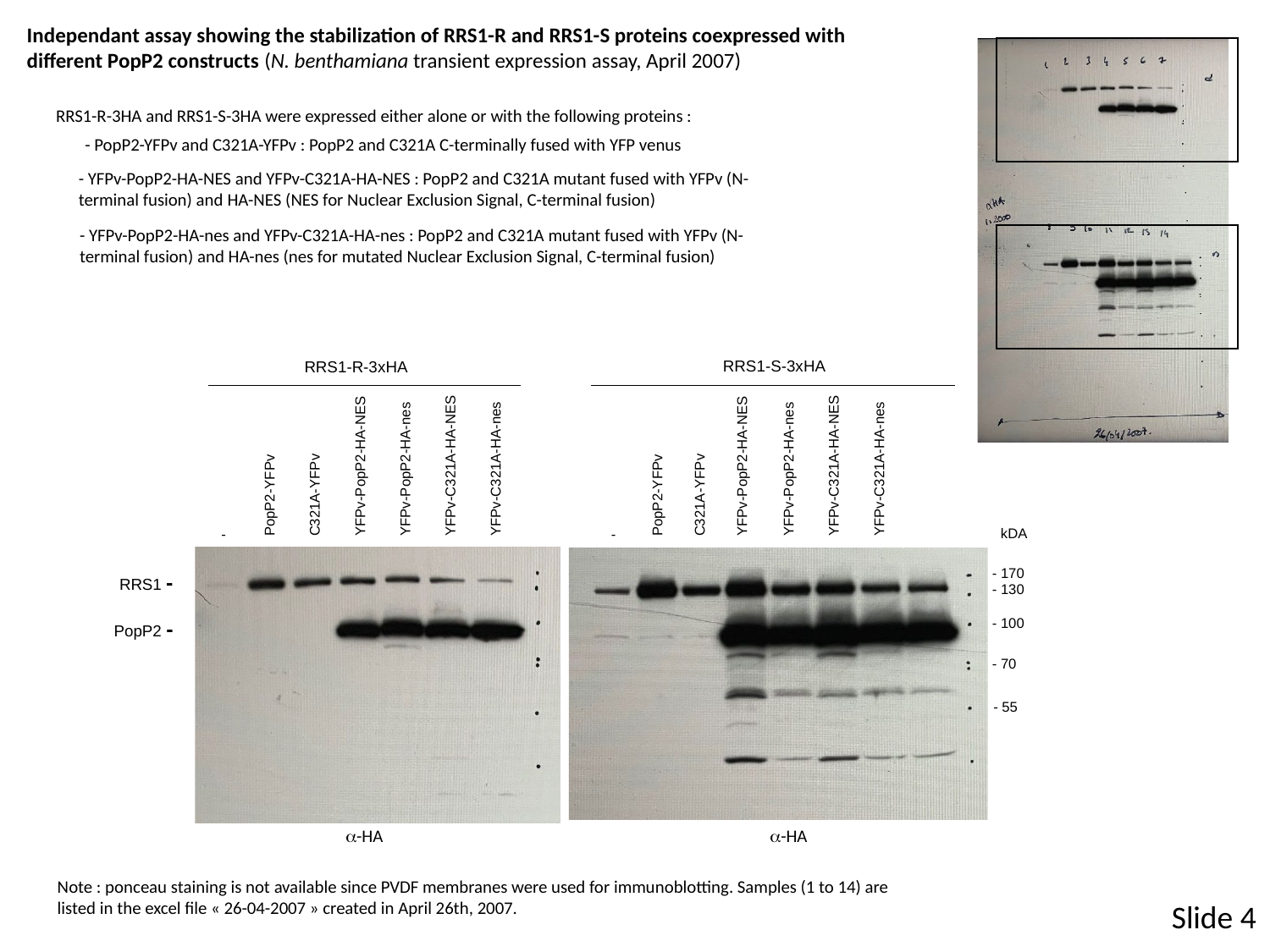

Independant assay showing the stabilization of RRS1-R and RRS1-S proteins coexpressed with different PopP2 constructs (N. benthamiana transient expression assay, April 2007)
RRS1-R-3HA and RRS1-S-3HA were expressed either alone or with the following proteins :
- PopP2-YFPv and C321A-YFPv : PopP2 and C321A C-terminally fused with YFP venus
- YFPv-PopP2-HA-NES and YFPv-C321A-HA-NES : PopP2 and C321A mutant fused with YFPv (N-terminal fusion) and HA-NES (NES for Nuclear Exclusion Signal, C-terminal fusion)
- YFPv-PopP2-HA-nes and YFPv-C321A-HA-nes : PopP2 and C321A mutant fused with YFPv (N-terminal fusion) and HA-nes (nes for mutated Nuclear Exclusion Signal, C-terminal fusion)
RRS1-S-3xHA
RRS1-R-3xHA
YFPv-C321A-HA-NES
YFPv-C321A-HA-NES
YFPv-PopP2-HA-NES
YFPv-PopP2-HA-NES
YFPv-C321A-HA-nes
YFPv-C321A-HA-nes
YFPv-PopP2-HA-nes
YFPv-PopP2-HA-nes
C321A-YFPv
C321A-YFPv
PopP2-YFPv
PopP2-YFPv
kDA
-
-
- 170
RRS1 -
- 130
- 100
PopP2 -
- 70
- 55
a-HA
a-HA
Note : ponceau staining is not available since PVDF membranes were used for immunoblotting. Samples (1 to 14) are listed in the excel file « 26-04-2007 » created in April 26th, 2007.
Slide 4

## Slide 5
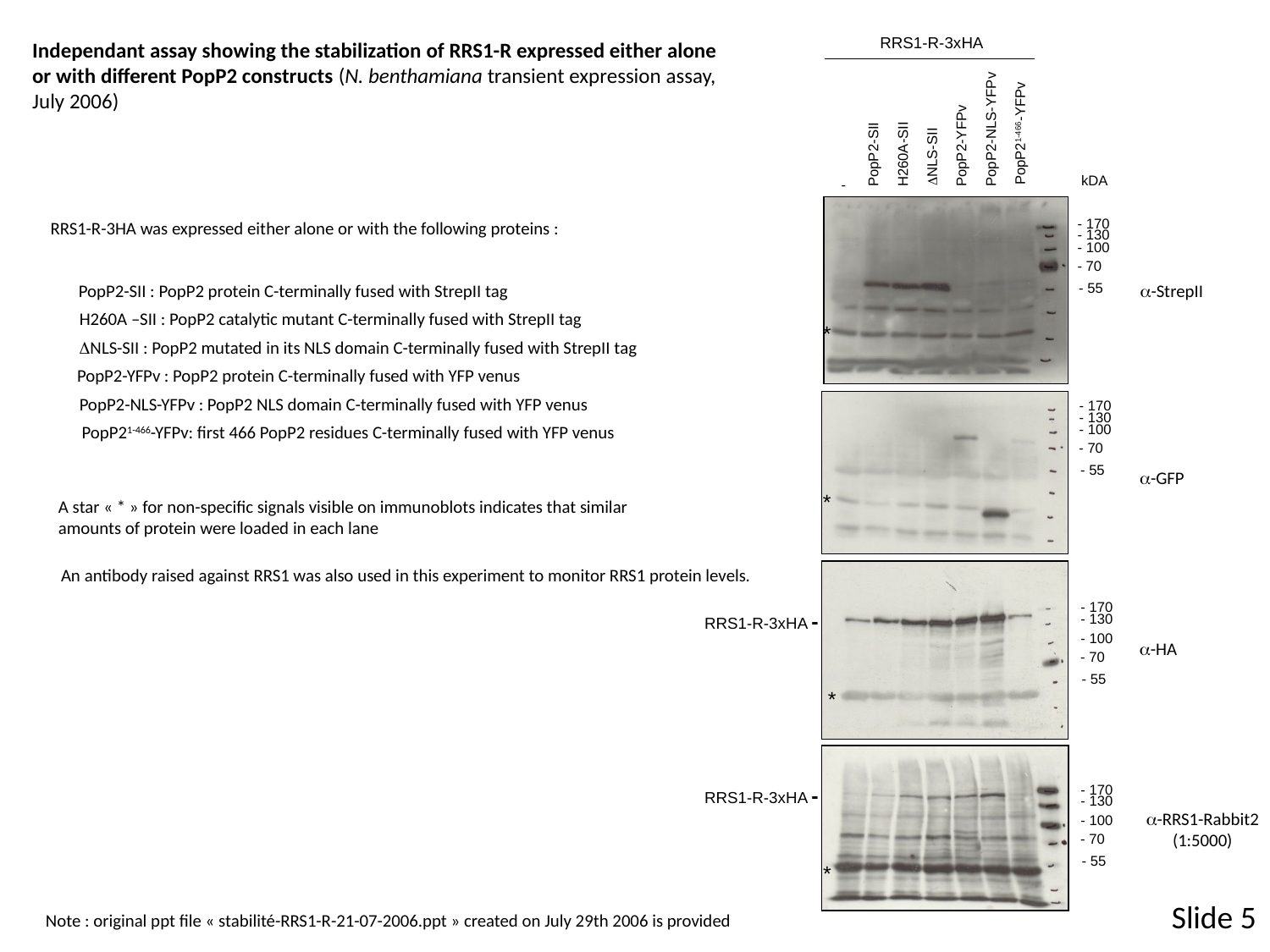

RRS1-R-3xHA
Independant assay showing the stabilization of RRS1-R expressed either alone or with different PopP2 constructs (N. benthamiana transient expression assay, July 2006)
PopP2-NLS-YFPv
PopP21-466-YFPv
PopP2-YFPv
H260A-SII
PopP2-SII
DNLS-SII
kDA
-
- 170
RRS1-R-3HA was expressed either alone or with the following proteins :
- 130
- 100
- 70
- 55
a-StrepII
PopP2-SII : PopP2 protein C-terminally fused with StrepII tag
H260A –SII : PopP2 catalytic mutant C-terminally fused with StrepII tag
*
DNLS-SII : PopP2 mutated in its NLS domain C-terminally fused with StrepII tag
PopP2-YFPv : PopP2 protein C-terminally fused with YFP venus
PopP2-NLS-YFPv : PopP2 NLS domain C-terminally fused with YFP venus
- 170
- 130
- 100
PopP21-466-YFPv: first 466 PopP2 residues C-terminally fused with YFP venus
- 70
- 55
a-GFP
*
A star « * » for non-specific signals visible on immunoblots indicates that similar amounts of protein were loaded in each lane
An antibody raised against RRS1 was also used in this experiment to monitor RRS1 protein levels.
- 170
RRS1-R-3xHA -
- 130
- 100
a-HA
- 70
- 55
*
- 170
RRS1-R-3xHA -
- 130
a-RRS1-Rabbit2 (1:5000)
- 100
- 70
- 55
*
Slide 5
Note : original ppt file « stabilité-RRS1-R-21-07-2006.ppt » created on July 29th 2006 is provided
