# Supplementary material for: Correction: Autoacetylation of the Ralstonia solanacearum Effector PopP2 Targets a Lysine Residue Essential for RRS1-R-Mediated Immunity in Arabidopsis
Source: PLoS Pathog. 2022 Mar 2;18(3):e1010368. doi: 10.1371/journal.ppat.1010368 (PMC8890644; doi:10.1371/journal.ppat.1010368)
Supplement: S3 File — (PPTX) [file ppat.1010368.s003.pptx]

## Slide 1
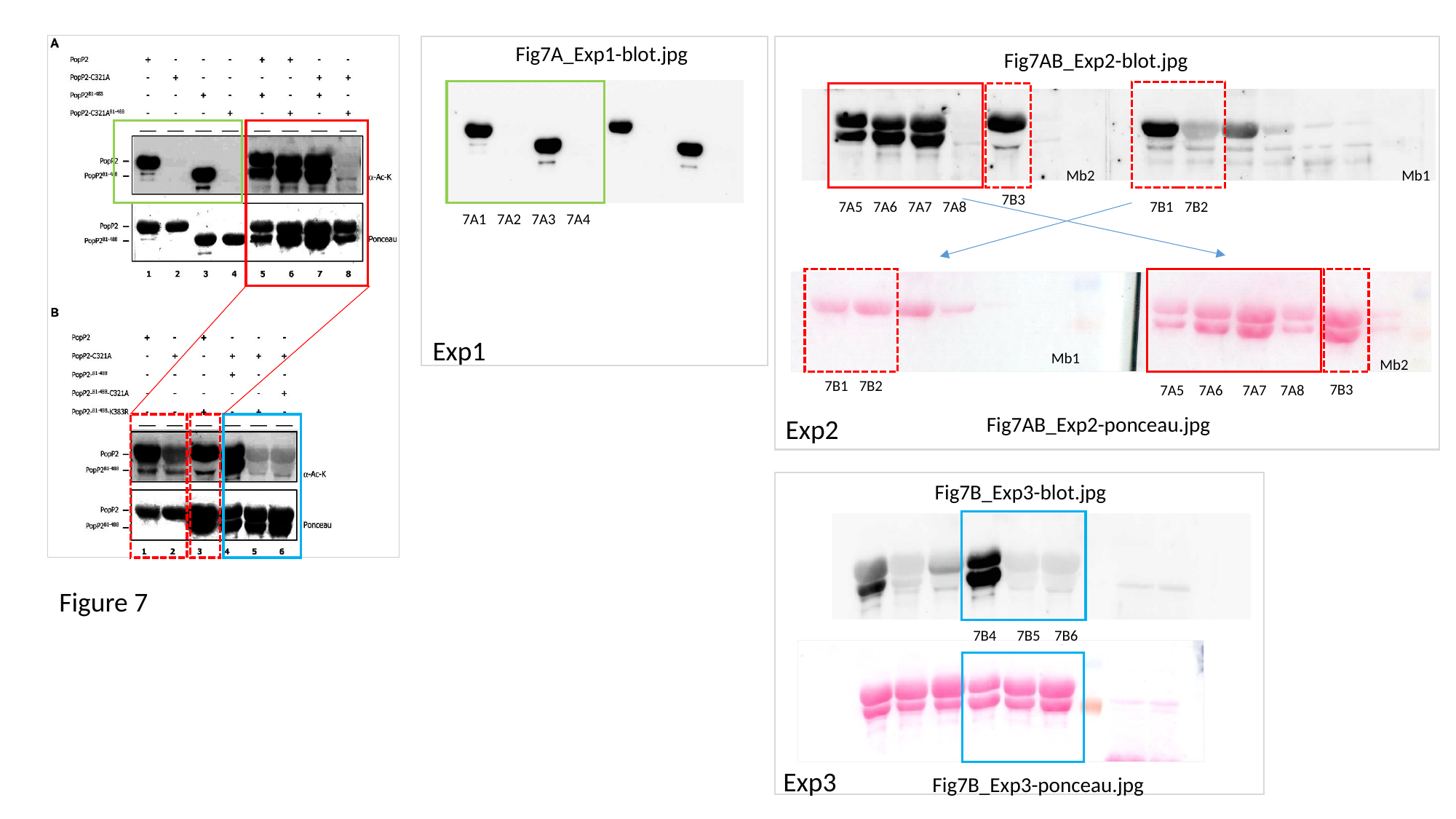

Fig7A_Exp1-blot.jpg
Fig7AB_Exp2-blot.jpg
Mb2
Mb1
7B3
7A5
7A6
7A7
7A8
7B1
7B2
7A1
7A2
7A3
7A4
Exp1
Mb1
Mb2
7B1
7B2
7B3
7A5
7A6
7A7
7A8
Fig7AB_Exp2-ponceau.jpg
Exp2
Fig7B_Exp3-blot.jpg
Figure 7
7B4
7B5
7B6
Exp3
Fig7B_Exp3-ponceau.jpg
