# Supplementary figures and images for: Correction: Autoacetylation of the Ralstonia solanacearum Effector PopP2 Targets a Lysine Residue Essential for RRS1-R-Mediated Immunity in Arabidopsis
Source: PLoS Pathog. 2022 Mar 2;18(3):e1010368. doi: 10.1371/journal.ppat.1010368 (PMC8890644; doi:10.1371/journal.ppat.1010368)

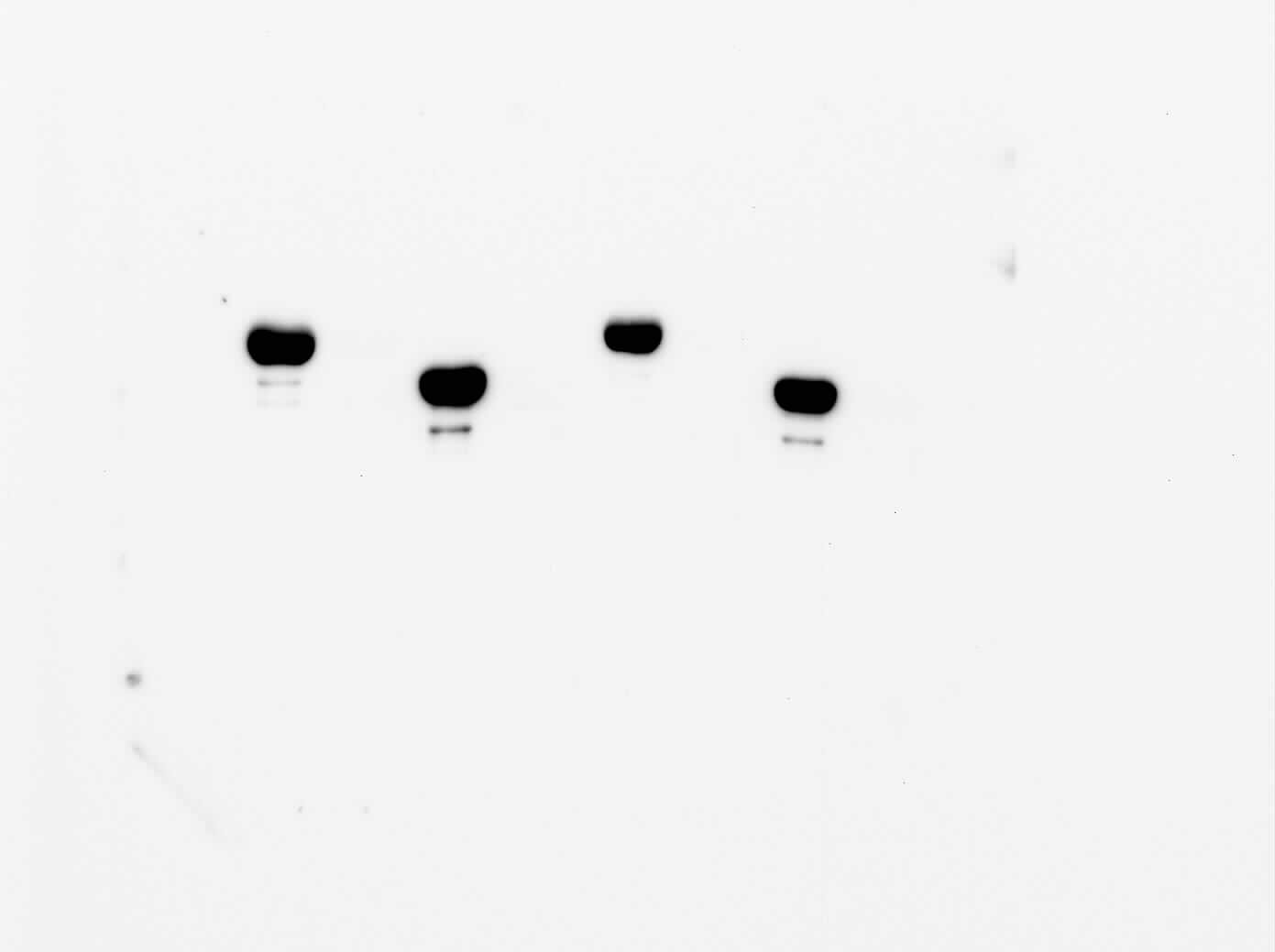

Supplement: S4 File — (ZIP) [file ppat.1010368.s004.zip › Fig7A_Exp1-blot.jpg]

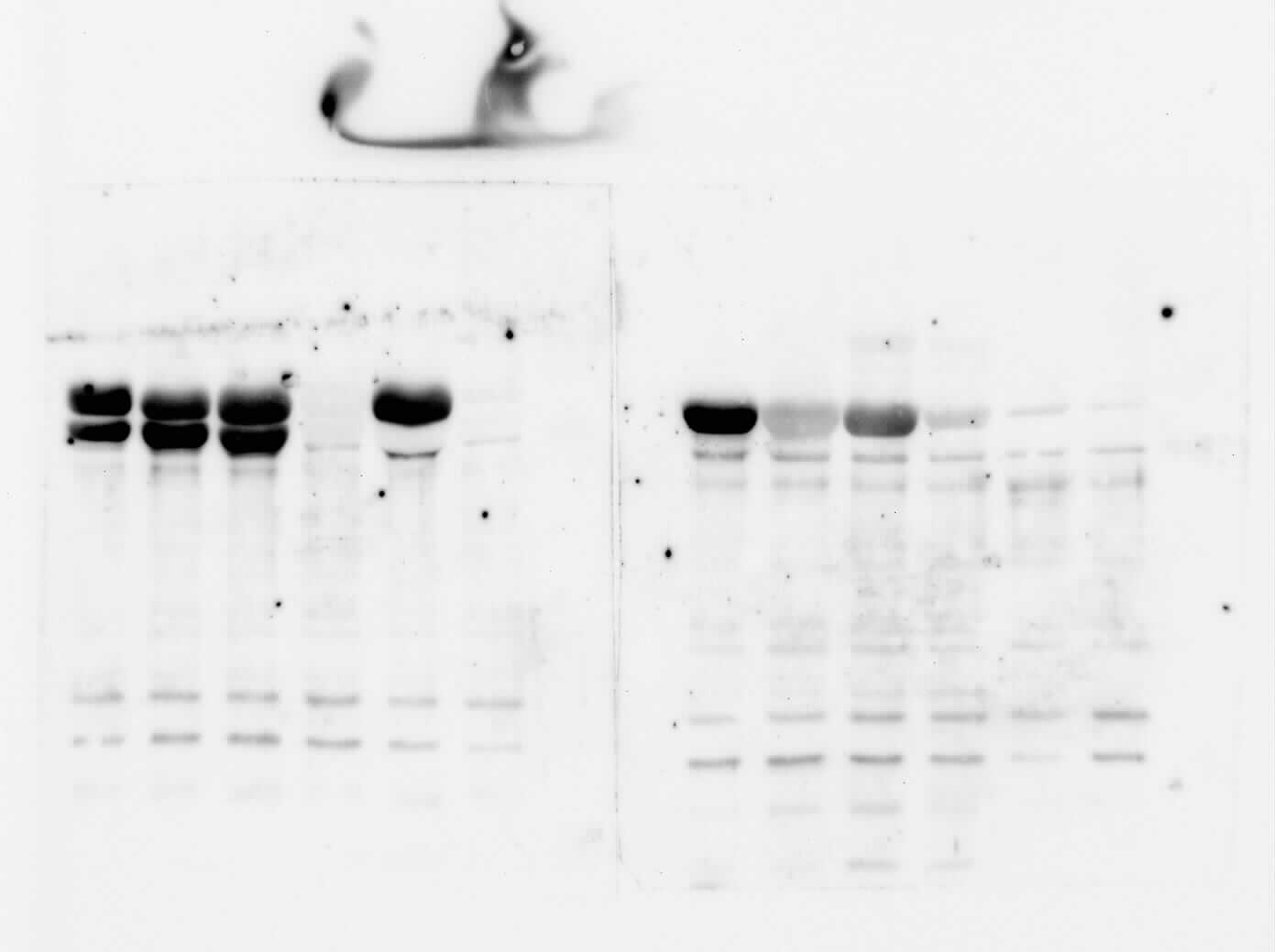

Supplement: S4 File — (ZIP) [file ppat.1010368.s004.zip › Fig7AB_Exp2-blot.jpg]

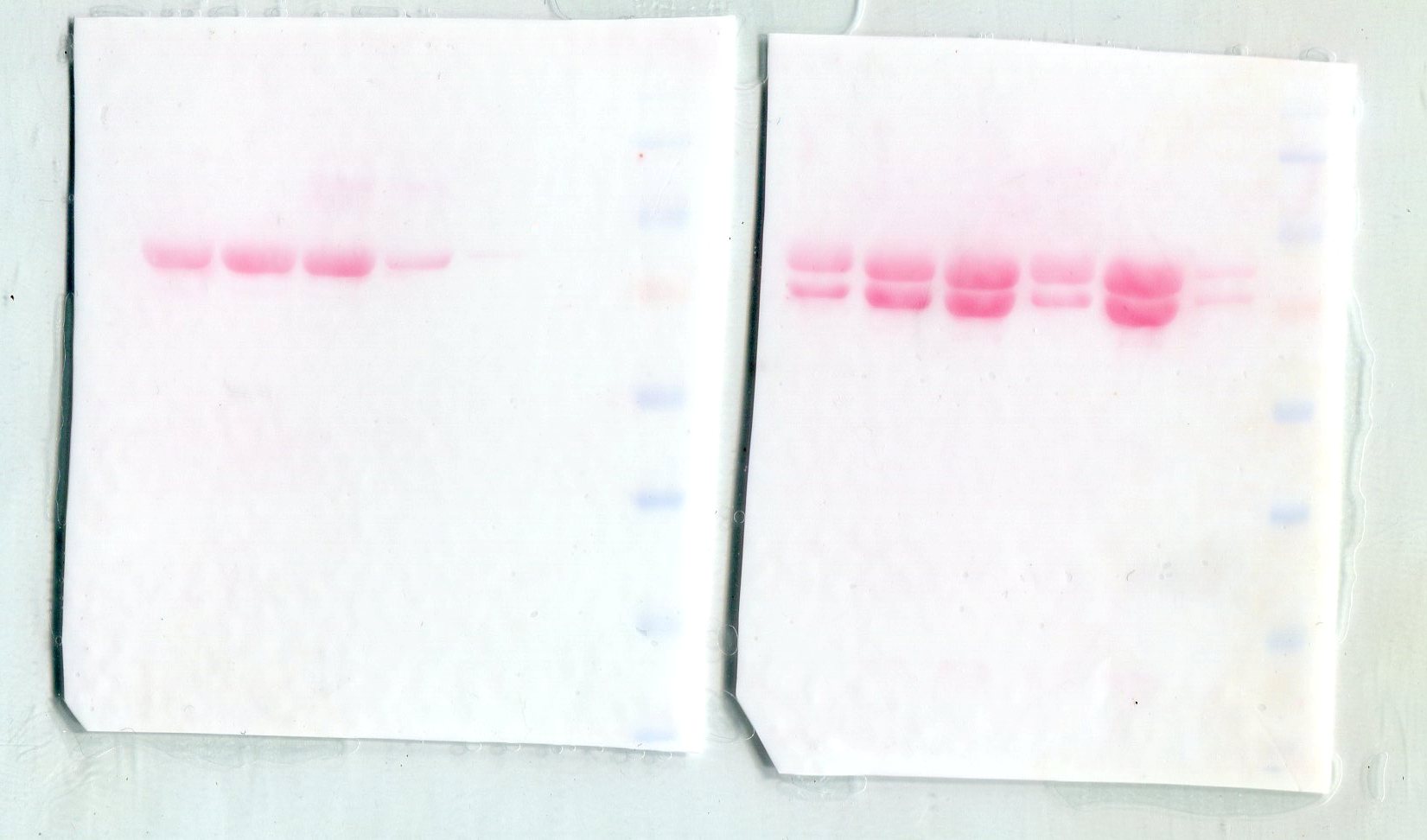

Supplement: S4 File — (ZIP) [file ppat.1010368.s004.zip › Fig7AB_Exp2-ponceau.jpg]

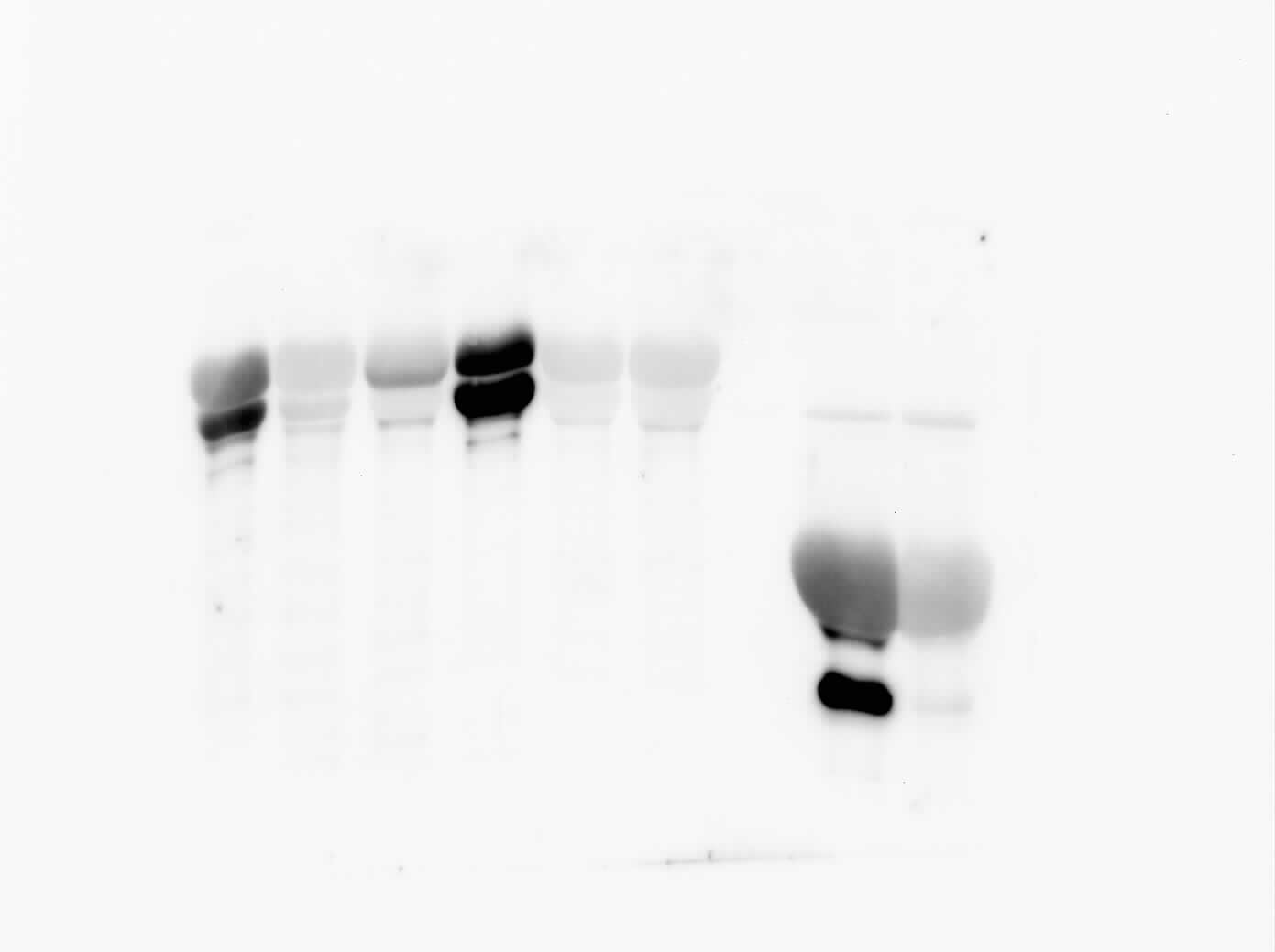

Supplement: S4 File — (ZIP) [file ppat.1010368.s004.zip › Fig7B_Exp3-blot.jpg]

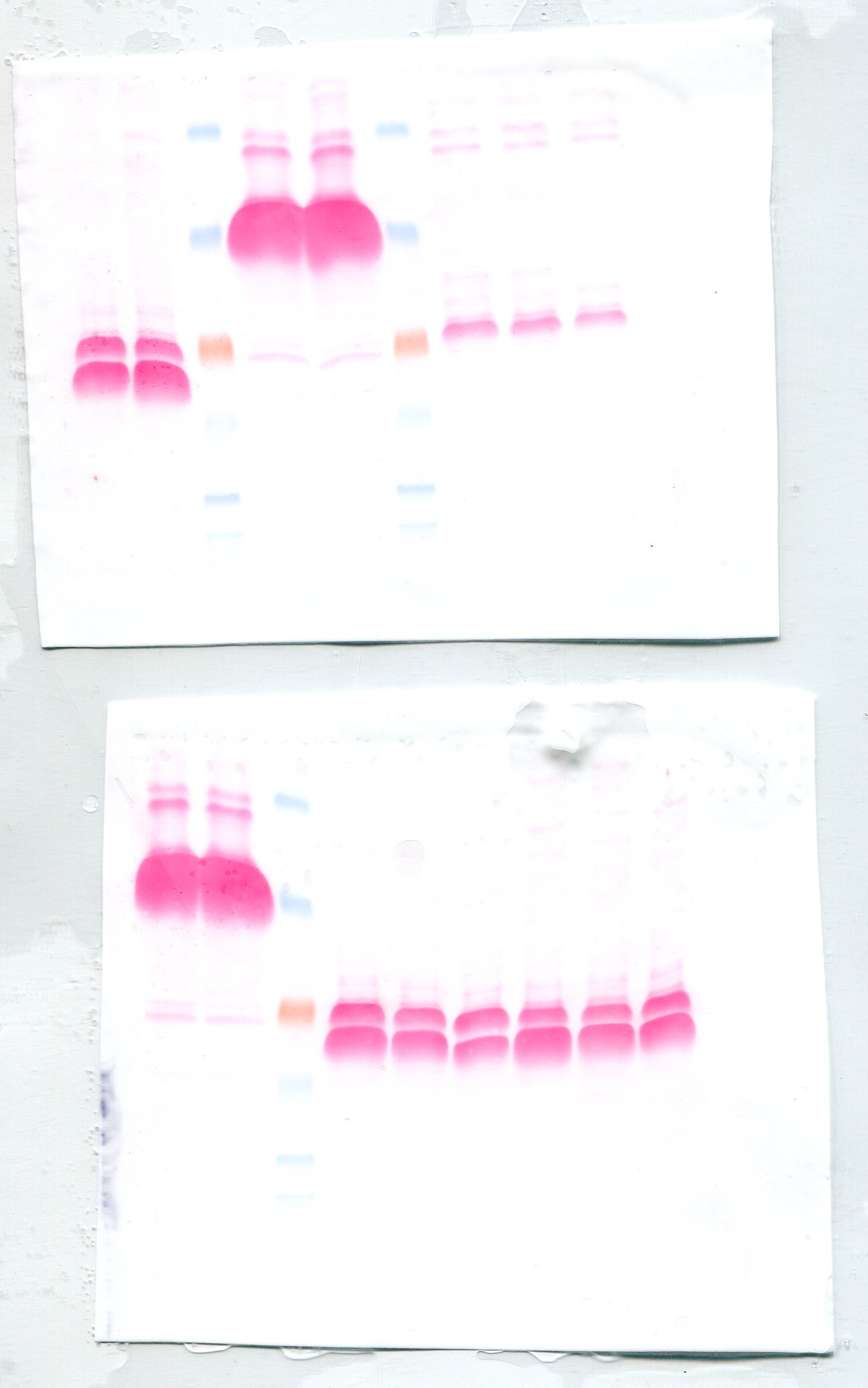

Supplement: S4 File — (ZIP) [file ppat.1010368.s004.zip › Fig7B_Exp3-ponceau.jpg]

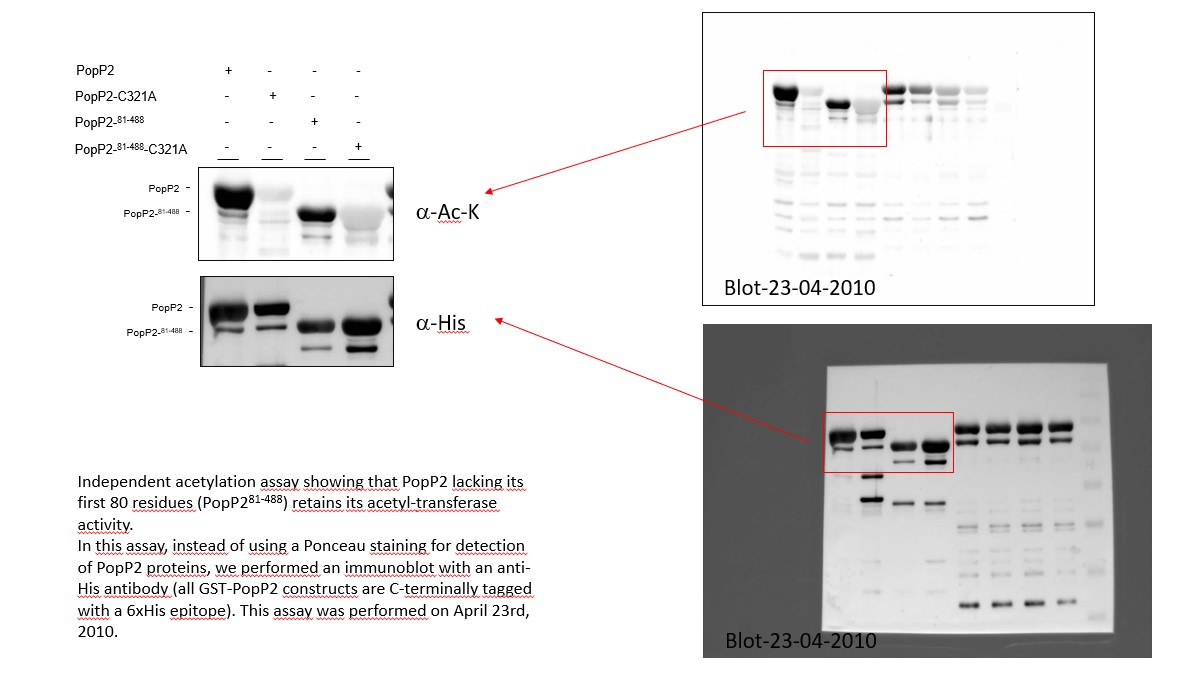

Supplement: S5 File — (TIF) [file ppat.1010368.s005.tif]
